# Tunable Wettability of a Dual-Faced Covalent Organic Framework Membrane for Enhanced Water Filtration

**DOI:** 10.1021/jacs.4c07559

**Published:** 2024-08-07

**Authors:** Farah Benyettou, Asmaa Jrad, Zineb Matouk, Thirumurugan Prakasam, Houeida Issa Hamoud, Guillaume Clet, Sabu Varghese, Gobinda Das, Mostafa Khair, Sudhir Kumar Sharma, Bikash Garai, Rasha G. AbdulHalim, Maryam Alkaabi, Jamaliah Aburabie, Sneha Thomas, James Weston, Renu Pasricha, Ramesh Jagannathan, Felipe Gándara, Mohamad El-Roz, Ali Trabolsi

**Affiliations:** † Chemistry Program, New York University Abu Dhabi (NYUAD), Abu Dhabi 129188, United Arab Emirates; ‡ NYUAD Water Research Center, New York University Abu Dhabi (NYUAD), 129188 Abu Dhabi , United Arab Emirates; § Technology Innovative Institute, Abu Dhabi 9639, United Arab Emirates; ∥ ENSICAEN, UNICAEN, CNRS, LCS, Normandie Univ, Caen 14000, France; ⊥ Core Technology Platform, New York University Abu Dhabi, 129188 Abu Dhabi, United Arab Emirates; # Instituto de Ciencia de Materiales de Madrid-CSIC, C. Sor Juana Inés de la Cruz 3, Madrid 28049, Spain; †† Engineering Division, New York University Abu Dhabi, 129188 Abu Dhabi, United Arab Emirates

## Abstract

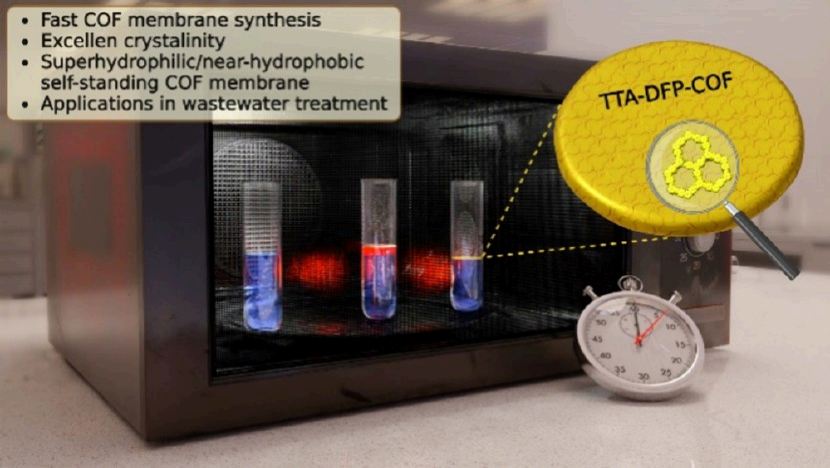

Membrane technology plays a central role in advancing separation processes, particularly in water treatment. Covalent organic frameworks (COFs) have transformative potential in this field due to their adjustable structures and robustness. However, conventional COF membrane synthesis methods are often associated with challenges, such as time-consuming processes and limited control over surface properties. Our study demonstrates a rapid, microwave-assisted method to synthesize self-standing COF membranes within minutes. This approach allows control over the wettability of the surface and achieves superhydrophilic and near-hydrophobic properties. A thorough characterization of the membrane allows a detailed analysis of the membrane properties and the difference in wettability between its two faces. Microwave activation accelerates the self-assembly of the COF nanosheets, whereby the thickness of the membrane can be controlled by adjusting the time of the reaction. The superhydrophilic vapor side of the membrane results from −NH_2_ reactions with acetic acid, while the nearly hydrophobic dioxane side has terminal aldehyde groups. Leveraging the superhydrophilic face, water filtration at high water flux, complete oil removal, increased rejection with anionic dye size, and resistance to organic fouling were achieved. The TTA-DFP-COF membrane opens new avenues for research to address the urgent need for water purification, distinguished by its synthesis speed, simplicity, and superior separation capabilities.

## Introduction

Membrane technology has gained considerable attention in the field of filtration due to its ability to efficiently replace conventional energy-intensive separation techniques.^1,2^ Developing membranes with a bottom-up approach and the flexibility to control their porous structure and surface properties could represent a breakthrough in many separation processes, particularly in water treatment.^3,4^ Reticular materials, such as metal–organic frameworks (MOFs) and covalent organic frameworks (COFs), offer unprecedented opportunities in the bottom-up approach for membrane synthesis.^5−7^ Their precisely tunable structures and self-assembling behavior enable the controlled arrangement of building blocks to create well-defined membrane structures with tailored properties.^8^ COFs have emerged as an intriguing family of porous nanomaterials composed of lightweight elements (C, N, O, B, etc.) that exhibit excellent structural diversity, tunable and permanent porosity, ordered structures, and high thermal and chemical stability.^9−11^ These properties make COFs attractive for various applications such as gas adsorption,^12,13^ water treatment,^14^ energy storage,^15,16^ sensing,^17,18^ drug delivery,^19−23^ and catalysis.^24,25^ Most COFs synthesized by the conventional solvothermal procedure are obtained as insoluble powders. Many attempts have been made to develop COF-based membranes, including mixed matrix membranes and self-standing membranes by interfacial polymerization.^26−30^ COF-based mixed matrix membranes are easy to make, versatile, and could change the properties of polymeric membranes, but the porous structure of the polymeric matrix determines the filtration efficiency.^29^ On the other hand, interfacial polymerization has been used to prepare self-standing COF membranes, but their synthesis is time-consuming and could take many days, and they are mostly grown as a thin film on a substrate (Table S1).^30^ In addition, the poor solubility of most aromatic amine building blocks makes it challenging to apply interfacial polymerization for COF membranes’ synthesis at a liquid–liquid interface.^31^ Interfacial polymerization at the liquid–air interface has also been reported for the fabrication of self-standing COF membranes.^32,33^ However, a Langmuir–Blodgett method was required to transfer the COF thin film from water to a substrate, and the process had to be repeated to obtain a robust COF filtration membrane. In addition, the COF monomers must have an amphiphilic nature to interpose between the organic phase and water interface. While synthesizing COFs as membranes is important, it is essential to ensure that the process is both rapid and straightforward, requiring as few steps as possible to enable large-scale production. Moreover, many studies on COF membranes focus on controlling their pore size,^34−36^ but reports on controlling the wettability of the membrane surface remain rare, even though it is one of the most important physicochemical properties of membranes.^37^ The organic nature of COFs and their hydrophobicity make it challenging to modify the surface wettability of a COF membrane without changing its structure’s building blocks, functionalizing it, or treating it post-synthetically, which could be time and energy-consuming. For this reason, most studies focusing on the surface wettability of COF membranes have attempted to produce superhydrophobic membranes or membrane coatings.^38,39^ However, the studies in polymeric membranes are shifting to the fabrication of hydrophilic and superhydrophilic membranes and membrane coatings to increase water flux and reduce organic fouling of membranes, which is one of the major challenges for their commercialization.^40−42^ Therefore, developing a fast and simple method to synthesize self-standing COF membranes with tunable hydrophilicity is challenging but essential for industrial membrane applications. Recently, a self-standing COF membrane was synthesized by covalently linking the building blocks at the liquid–solid interface and used for membrane distillation.^37^ The surfaces of the membranes were then modified by a reverse imine-bond formation reaction to produce a hydrophilic surface, reduce membrane fouling, and increase water flux. However, the membrane preparation process takes up to 4 days, and treating it with an alkaline solution to obtain a hydrophilic surface takes an additional 18 to 24 h, necessitating nearly 5 days to make the modified membrane. In another study, solvent-induced fragmentation was used to tune the surface wettability of a self-standing 3D COF membrane, resulting in a higher hydrophobicity of the membrane surface.^43^ However, the membrane preparation takes 2 days, and the study focuses on hydrophobic and superhydrophobic COF membrane surfaces.

In this study, we have successfully synthesized a series of dual superhydrophilic/near-hydrophobic self-standing imine-linked TTA-DFP-COF membranes. The novelty of this study lies in the rapid and one-step membrane synthesis and the ability to control the surface properties of the membrane without subsequent modification. This was made possible by a microwave-mediated interfacial self-assembly method at the liquid-water vapor interface within a few minutes. The control of surface wettability was made possible by increasing the synthesis reaction time. While the side of the membrane in contact with water vapor is superhydrophilic, the side in contact with dioxane becomes nearly hydrophobic by extending the reaction time. Moreover, the synthesis time reported in this study is much shorter than that of the previously reported COF membranes (Table S1). Both surface chemistry and roughness contribute to this variation in wettability, with the superhydrophilic side exhibiting a roughness reduced by a factor of 10 when exposed to humid air. To investigate the molecular composition and structure of the membrane, we used Raman, ATR-FTIR, XPS, AFM, and TEM. The hydrophilic nature of the vapor face was found to be the result of the reaction between the terminal −NH_2_ groups of the triamine precursor and aqueous acetic acid. In contrast, the dioxane face was characterized by dominant terminal aldehyde groups, which gave it a near-hydrophobic character. Comprehensive characterizations of the COF membrane demonstrated its crystallinity, stability, and adaptability in surface wetting. Samples were taken from the membrane at varying times during its synthesis, and their TEM/STEM analyses and morphological studies highlighted the role of microwave activation in the synthesis. It promotes the mesoscale self-assembly of COF nanosheets at the liquid–vapor interface. The influence of water condensation and focused microwave energy led to the formation of a membrane with different surface textures: a smooth, super hydrophilic vapor face and a textured near-hydrophobic dioxane face. The formation of this thick membrane begins in a few minutes and continues to grow in bulk until the building blocks are consumed, allowing control of the thickness. The detailed exploration of the formation dynamics of TTA-DFP-COF membranes demonstrated in this study, in conjunction with the comprehensive analysis of wettability differences on the surfaces, represents a significant advancement in the rapid synthesis of COF membranes with tunable wettability profiles tailored to precise applications.

To leverage the superhydrophilicity of the vapor face of the membrane, water filtration experiments were performed with salts, dyes, and mineral oil using vacuum filtration, where efficient filtration at a high water flux was observed. The results indicate a correlation between the membrane’s rejection efficiency and the pollutant’s molecular size, suggesting a possible contribution to rejection by molecular sieving, in addition to electrostatic repulsion from the negatively charged membrane surface. The efficiency of the superhydrophilic membrane face was also investigated in oil-in-water emulsion filtration and showed excellent oil rejection at high water flux. Furthermore, the membrane showed strong antimicrobial and antibiofouling properties against both Gram-negative (*E. coli*) and Gram-positive (*S. aureus*) bacteria while being biocompatible. This property is important for water filtration membranes as it increases their effectiveness in preventing the adhesion of bacteria and the development of biofilms and ensures a consistent, clean water output.

Our approach combines microwave-assisted synthesis with a novel self-assembly technique at the dioxane-water vapor interface. This study provides a method to regulate the wettability of COF membrane surfaces and a deep understanding of this phenomenon, which opens up ways to fine-tune membranes’ properties for specific applications. The superhydrophilic membrane surface enables fast water flux while increasing resistance to fouling and ensuring high rejection rates for various pollutants. Considering the global water challenges, our TTA-DFP-COF membrane offers an innovative approach with immense potential for real-world water treatment applications and represents a step forward in sustainable purification technologies.

## Results and Discussion

### Membrane Synthesis and Characterization

**Figure 1:**
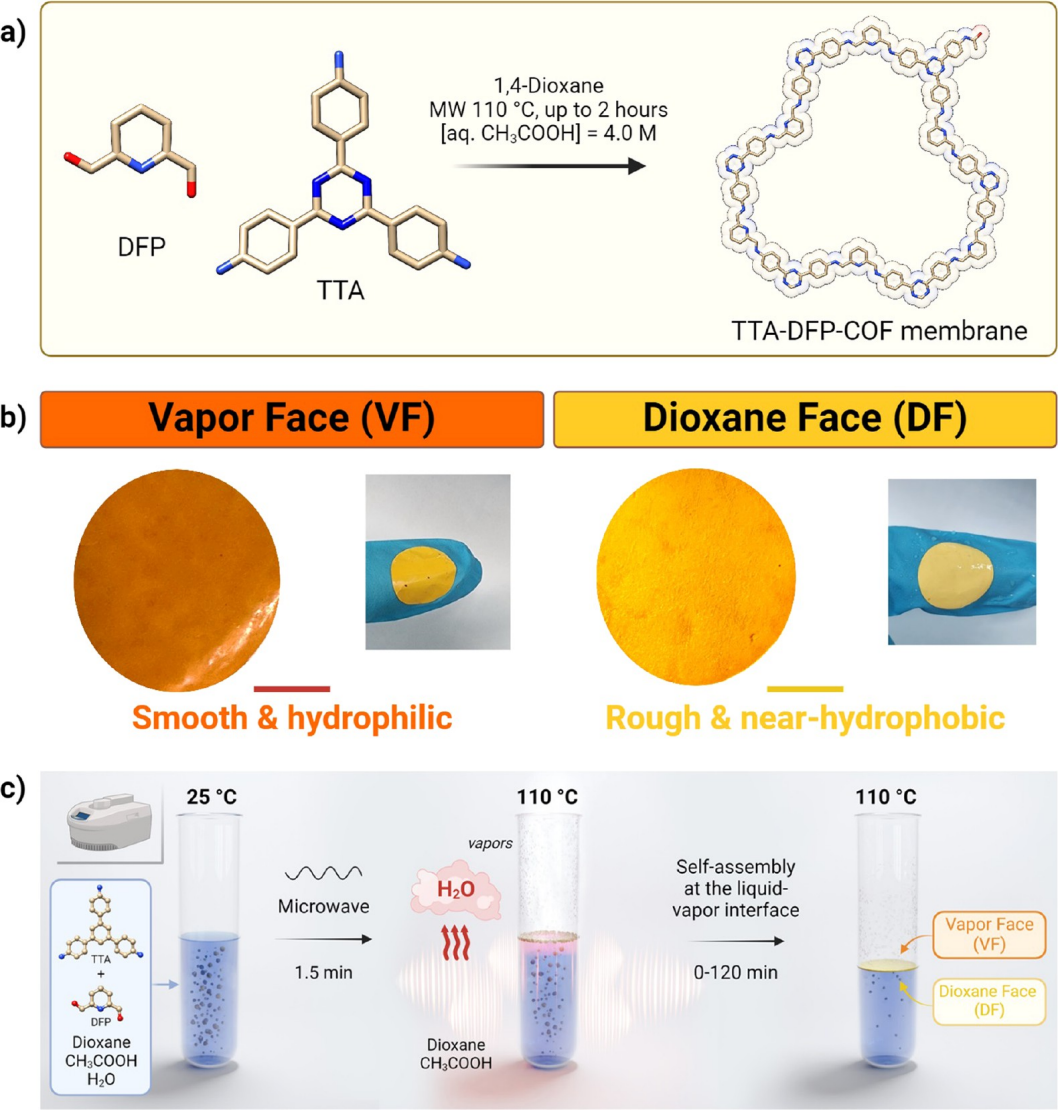
Self-assembly at the dioxane–water vapor interface of a continuous, defect-free, highly crystalline, and porous self-standing COF membrane. (a) Chemical structure and synthesis pathway of the TTA-DFP-COF membranes obtained under microwave irradiation. (b) Digital images of the TTA-DFP-COF membrane faces: the vapor face is smooth and glossy in comparison with the rough and matte texture of the dioxane face. Scale bar = 1 cm. (c) Schematic representation of the membrane formation inside the microwave vessel. The self-standing TTA-DFP-COF membrane is formed due to the confinement of the polymerization of the aldehyde and amine monomers at the interface between dioxane and water vapors under microwave irradiation (300W).

Self-standing COF membranes (denoted as TTA-DFP-COF membrane) with different thicknesses—25, 55, and 85 μm—were prepared by covalently linking 2,6-diformylpyridine (DFP, 21 mg, 0.15 mmol, 5 equiv) and 4,4′,4″-(1,3,5-triazine-2,4,6-triyl)trianiline (TTA, 12 mg, 0.03 mmol, 1 equiv), in 3 mL of anhydrous 1,4-dioxane and in the presence of 0.5 mL of aqueous acetic acid (13 M, [acetic acid]_final_ = 4.0 M) at 110 °C under microwave irradiations (300 W, Figure 1a and Figure S1). The membrane formed at the liquid–vapor interface as observed in Movie S1. By adjusting the reaction time during microwave irradiation to 5, 45, and 120 min intervals, we could control the thickness of the membrane. The membranes are denoted TTA-DFP-COF-**5**, TTA-DFP-COF-**45**, and TTA-DFP-COF-**120**, where **5**, **45**, and **120** represent the reaction time in minutes. After the specified reaction time, a free-standing membrane was obtained, showing no apparent cracks. The membrane displayed a smooth and glossy face on its upper side (contact with water vapors), referred to as the vapor face (VF), whereas the dioxane face (DF, contact with the solution) showed a rough and matte appearance (Figure 1b, Movie S2). Retrieving the membrane was easily accomplished using tweezers, and it could be cleaned multiple times with dioxane and ethanol (Movie S3).

In contrast to alternative methods for preparing COF membranes, our strategy stands out for its simplicity and speed. The preparation process of TTA-DFP-COF membranes can be completed in a few minutes, making it one of the fastest approaches for COF membrane preparation to date (Table S1). The linkers (DFP and TTA) are mixed in dioxane and sonicated for a few seconds to dissolve. Then, aqueous acetic acid was added rapidly, and the mixture was placed immediately in the microwave oven (300 W) to initiate heating (Figure 1c). The temperature quickly increases to 110 °C in 1.5 min. During the ramp, the 125 μL of water (from the aqueous acetic acid) starts boiling, and the first bubbles are observed around 80 °C after 40 s of ramping and keep boiling for 30 s until 110 °C is reached (Movie S1). Water condensation can be observed on the vessel walls. The emergence of a film can be observed at the interface between the vapor and liquid phases. We previously demonstrated that at room temperature, a small amount of water favors the stacking of small nanosheets, leading to nanoparticle formation.^44^ The control experiment with dry acetic acid did not lead to membrane formation, demonstrating the important role of water in the mechanism.

Microwave (MW) energy revolutionizes chemical synthesis by directly energizing the sample for quicker, more even heating, making it highly effective for producing COFs with superior quality and efficiency.^45^ Despite its advantages, reports on COF membranes synthesized with MW as heating source are lacking. Microwave irradiation is essential for TTA-DFP-COF membrane synthesis; it causes water to evaporate and creates a vapor layer, enabling localized heating at the liquid–vapor interface, which is critical for the formation of the membrane. Due to their distinct polar and ionic properties, solvents exhibit varying interactions when exposed to microwaves. Polar solvents like water exhibit efficient diffusion under microwave irradiation due to their high dielectric constants, interactions with electromagnetic fields, and dipolar characteristics. As a consequence, the temperature of the solvent rises significantly during this process. Nonpolar solvents like dioxane can only be heated in the presence of other components in the reaction mixture that respond to microwave energy, such as water or acetic acid.^46^ In such cases, it is possible to achieve high temperatures. In our particular situation, as water evaporates and recondenses at the liquid–vapor interface, the conduction of microwave heating becomes locally significantly elevated. As a result, the focused application of heat at the dioxane-water vapor interface precisely moderates the diffusion of acetic acid, which in turn initiates the selective polymerization of unreacted DFP aldehyde groups, terminal aldehyde groups, and free amine groups on the nanosheets. This targeted polymerization at the interface culminates in the assembly of the COF membrane, a process depicted in Figure 1c and further elucidated in Movie S4A–C. Control experiments using an oven and an oil bath as heating sources, as opposed to microwave irradiation, failed to produce membranes, yielding only powder, underscoring the essential nature of MW energy in this process (Figure S3). This process takes place without stirring, which ensures the undisturbed distribution of the various solvents. As a result, the heating energy is not uniformly distributed throughout the mixture but is concentrated in the areas with the more polar solvents. The control experiment with stirring did not lead to membrane formation, which shows the importance of not disturbing the solvent post-organization (Movie S5). The control experiment, conducted with microwave power reduced to 100 W, successfully synthesized a COF membrane, but it was less robust than those produced at 300 W. This discrepancy likely results from slower solvent rearrangement and heat distribution at lower power, both crucial for early stage polymerization at the interface (Figure S4). Upon the initiation of membrane polymerization, prolonging the duration leads to the accumulation of additional thin layers of COF under the existing ones, gradually increasing the overall thickness of the membrane.

FTIR and solid-state NMR spectroscopies were used to analyze the chemical composition of the TTA-DFP-COF-**5/45/120** membranes. The findings revealed that all the membranes exhibited identical patterns regarding their characteristics independently of their thickness.

FTIR analysis provided detailed insights into the molecular architecture of the COF membranes. The monomers exhibit characteristic spectral features, with amine monomers (TTA) showing NH_2_ stretching vibrations in the 3460–3320 cm^–1^ range and aldehyde monomers (DFP) characterized by a C=O stretch at 1711 cm^–1^ (Figures S5 and S6). The transition to imine COFs was marked by the disappearance of the NH_2_ peak, the reduction of the C=O signal, and the appearance of an imine C=N stretch at 1621 cm^–1^, evidence of polycondensation. Other spectral features included a C=N stretch at 1507 cm^–1^, a broad C–N peak at 1364 cm^–1^ related to the triazine core, and a C=C stretch at 1580 cm^–1^.^47^ The spectrum also shows a weak C=O band at 1706 cm^–1^ originating from residual aldehydes and a distinct amide C=O stretch at 1680 cm^–1^, with accompanying amide C–N stretching at 1242 cm^–1^ and C–N–C stretching at 1325 cm^–1^. These bands indicate the formation of terminal *N*-phenyl acetamide groups, likely from the reaction of the TTA linker with acetic acid (Figures S5, S6 and S7).

^13^C CP-MAS solid-state NMR spectroscopy was employed to study the chemical environment at the atomic level of the obtained TTA-DFP-COF membranes (Figure S8). The ^13^C CP/MAS solid-state NMR spectrum obtained from TTA-DFP-COF membranes (Figure S8a) is well resolved and reveals mainly peaks originating from the aromatic (100 to 150 ppm) and aromatic imine carbon atoms (150 to 175 ppm). The formation of the new imine bond is confirmed by the new peak at ∼160 ppm. The spectrum is also characterized by the presence of terminal aldehyde groups appearing at ∼194 ppm. Chemical shift assignments were further confirmed by recording the solid-state NMR spectra of the starting materials (TTA and DFP) and the final TTA-DFP-COF membranes (Figure S8b).

The differences between the TTA-DFP-COF-**5/45/120** membrane properties were analyzed using SEM, PXRD, HRTEM, and N_2_ adsorption. The mechanical properties and the surface wettability of the membranes were also studied and they were found to be influenced by the variation in the membranes’ thicknesses.

**Figure 2:**
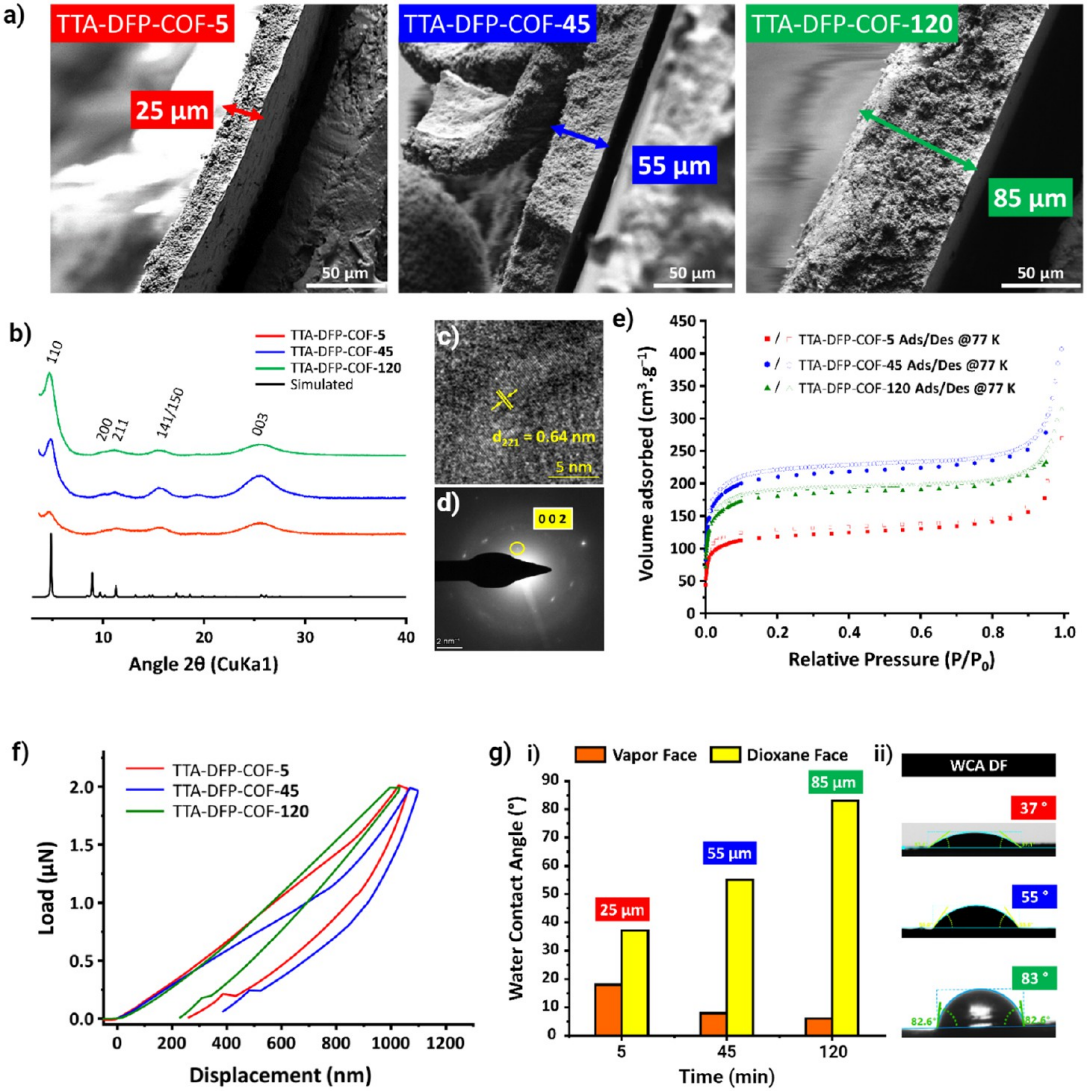
Structural characterization of the TTA-DFP-COF membranes. (a) SEM Cross-sectional images of TTA-DFP-COF-**5**, TTA-DFP-COF-**45**, and TTA-DFP-COF-**120**. (b) PXRD of TTA-DFP-COF-**5** (red), TTA-DFP-COF-**45** (blue), TTA-DFP-COF-**120** (green) and simulated (black). HRTEM analysis of TTA-DFP-COF-**120** confirming the material’s crystallinity: (c) HR-TEM image displaying lattice fringes (lattice fringe distances *d* = 0.64 nm) corresponding to the (221) plane of the COF. (d) Selective area electron diffraction (SAED) image indicates the TTA-DFP-COF membrane’s high crystallinity well matching to *d*_002_ plane with *d-*spacing of 0.51 nm. (e) N_2_ adsorption isotherms and (f) representative indentation load versus surface penetration depth curves of TTA-DFP-COF-**5** (red), TTA-DFP-COF-**45** (blue), and TTA-DFP-COF-**120** (green). (g) Surface wettability analysis: (i) water contact angle (WCA) of the vapor (VF, orange) and dioxane (DF, yellow) faces of TTA-DFP-COF-**5** (red), TTA-DFP-COF-**45** (blue), and TTA-DFP-COF-**120** (green), and (ii) corresponding WCA digital images of the DF of TTA-DFP-COF-**5/45/120**.

The inner morphology and thickness of TTA-DFP-COF membranes were investigated by SEM (Figure 2a and Figures S13–S17). The TTA-DFP-COF-**5/45/120** membranes display two distinct types of faces (Figures S15–17). The vapor face (VF), which was exposed to water vapor during synthesis, appears smooth, compact, and continuous. In contrast, the dioxane face (DF) appears rough. Notably, both faces are free from any cracks. As the reaction time increases, the contrast in characteristics between the VF and DF faces becomes more evident (Figures S15–S17). The inner structure of the TTA-DFP-COF-**5/45/120** membranes is made of a laminated arrangement consisting of multiple sheets, each measuring 5–10 μm (Figures S17 and S18). These sheets were stacked on top of one another, creating the characteristic structure shown in Figure S17. As the reaction time increased, the thickness of the membranes also increased, reaching 25, 55, and 85 μm for TTA-DFP-COF-**5**, TTA-DFP-COF-**45**, and TTA-DFP-COF-**120**, respectively, as observed using cross-section SEM (Figure 2a and Figures S13 and S14).

The crystalline structure of TTA-DFP-COF membranes was characterized using powder X-ray diffraction (PXRD), revealing clear evidence of crystallinity. Building upon reticular chemistry principles, crystal structure models of TTA-DFP-COF were constructed based on the geometries of the constituent building blocks. We generated models featuring distorted **hcb** layered structures within the trigonal *P3* space group. These models were geometrically optimized using universal force field energy minimizations, and their simulated pattern compared with the experimental one. The most accurate correlation between experimental and simulated PXRD patterns was achieved for layer-stacked structures following an ABC sequence. The unit cell parameter values determined after completing a Pawley refinement (Rwp = 1.94%, Rp = 1.55%) are *a* = *b* = 36.41 Å, *c* = 10.39 Å. Based on this structure, the distinct peak observed at 2θ = 4.8° corresponds to the (110) Bragg diffraction. Additional peaks at 2θ = 9.8, 11.2, and 15.5° correspond to the (200), (211), and combined (141) and (150) planes, respectively, alongside a higher-order peak at 2θ = 25.5° for the (003) plane (Figure 2b and Figure S20). The analysis also indicates that membrane thickness influences microstructural features like crystal packing, with thicker membranes showing stronger diffraction peak intensities, implying better crystallinity and internal order. This is demonstrated by the intensities of the (110) peak increasing with thickness, noted at 4800, 16100, and 19000 arbitrary units for membranes of varying thicknesses, with a significant rise in peak intensity observed as thickness increases from 25 to 55 μm before stabilizing between 55 and 85 μm (Figure S28).

HRTEM was then used to gain insight into the structure and the crystallinity of the COF membranes. The images show a highly ordered arrangement composed of a multilayered structure of individual COF nanosheets (Figure 2c,d and Figures S21 and S22), self-assembled due to interlayer π–π stacking with independent lattice fringes. Lattice-resolution TEM images of exfoliated TTA-DFP-COF membranes show that they are crystalline with consistent and continuous lattice fringes that extend across the entire COF (Figure 2c and Figure S23). The lattice spacings were 3.4 and 6.4 Å, as measured by fast Fourier transform, which match the expected *d*_003_ and *d*_221_ spacings, respectively. Furthermore, the selected area electron diffraction (SAED, Figure 2d and Figure S24) demonstrates the well-crystallized feature of the TTA-DFP-COF membrane, which exhibited distinct electron diffraction spots, well matching to *d*_002_ plane with a *d-*spacing of 5.1 Å.

The TTA-DFP-COF**-5/45/120** membranes exhibited permanent porosity as shown by N_2_ adsorption experiments, which yielded fully reversible type-I isotherms, indicative of nitrogen condensation within the membranes’ interstitial voids (Figure 2e, Table 1). The Brunauer–Emmett–Teller (BET) surface areas were calculated to be 448, 809, and 690 m^2^/g, with corresponding total pore volumes of 0.24, 0.39, and 0.33 cm^3^/g for the TTA-DFP-COF-**5/45/120** membranes, respectively (Figure S27). These data are consistent with the PXRD results that showed an increase in (110) peak intensity with membrane thickness up to 55 μm, after which it plateaus (Figure S28). This trend indicates the effect of microstructural differences on the surface area despite a consistent crystalline structure (Figure S28). Pore size analysis showed that TTA-DFP-COF**-5** has the largest average pore size (12.3 Å), in contrast to the smaller, uniform pore sizes (10.2 Å) of TTA-DFP-COF**-45** and TTA-DFP-COF**-120** (Figure S27). These physical properties, including BET surface area and pore distributions, reflect the degree of crystallinity of the membranes, which is influenced by the synthesis conditions such as reaction time and affect the thickness and packing of the membrane. Following on the research of Ma et al., who established a link between COF crystallinity and sorption properties,^48^ our results highlight the importance of synthesis precision in optimizing COF membranes for specific applications and improve our understanding of COF membrane fabrication.

**Table 1:** Physicochemical Properties of TTA-DFP-COF-5/45/120 Membranes^a^

	TTA-DFP-COF-**5**	TTA-DFP-COF-**45**	TTA-DFP-COF-**120**
thickness (μm)	24.8 ± 1.6	55.1 ± 1.8	85.7 ± 2.6
BET (m^2^ g^–1^), (TPV*, cm^3^ g^–1^)	448, (0.24)	809, (0.39)	690, (0.33)
pore width (Å)	12.3/14.8	10.2	10.2
Young modulus (MPa)	300 ± 100	500 ± 100	2100 ± 50
hardness (MPa)	250 ± 50	350 ± 50	520 ± 100
WCA, VF/DF (deg)	18/37	8/55	6/83

a TPV: total pore volume, WCA: water contact angle, VF: vapor face, DF: dioxane face.

Using our technique of self-assembly at the interface, we have successfully produced flexible and continuous TTA-DFP-COF membranes with a diameter of 2.5 cm. These membranes exhibit good mechanical strength, which facilitates their extraction from the mother solution and their transfer to diverse substrates (Movie S3). Therefore, the quantitative nanomechanical properties, Young’s modulus (*Er*), and hardness (*H*) were calculated based on the as-obtained load/depth curves (Figure 2f and Figure S29, Table 1). The TTA-DFP-COF-**120** showed the highest values for both parameters (*Er* = 2100 MPa and *H* = 520 MPa) among the three membranes (*Er* = 300 MPa and *H* = 250 MPa for TTA-DFP-COF-**5**; *Er* = 500 MPa and *H* = 350 MPa for TTA-DFP-COF-**45**). The TTA-DFP-COF-**120** membrane shows excellent mechanical properties (Table S2 for comparison with other reported membranes). Even after drying, the self-standing COF membranes could maintain their flexibility and integrity, which confirms their mechanical robustness (Movie S6).

To investigate the surface properties and wettability of the TTA-DFP-COF-**5/45/120** membranes, we conducted water contact angle (WCA) measurements on both the VF (exposed to water vapor) and DF (exposed to dioxane) faces, taking into account the variations in membrane thickness (Figure 2g and Figure S30). Significant differences in behavior were observed between the VF and DF faces of the membranes and as a function of the thickness. The vapor face of the TTA-DFP-COF-**5/45/120** membranes experienced almost complete wetting by water droplets, particularly in the case of TTA-DFP-COF-**120** with a WCA of 5.9°, as indicated in Table 1 and shown in Figure 2g and Figure S30. In contrast, the hydrophilicity of the dioxane face decreased relative to the membrane’s thickness and roughness. The contact angle exhibited an increase, reaching values of 37.1°, 55.0°, and 82.6° (almost hydrophobic) for TTA-DFP-COF**-5**, TTA-DFP-COF**-45**, and TTA-DFP-COF**-120**, respectively. Thus, the TTA-DFP-COF-**5/45/120** membranes displayed divergent face characteristics based on thickness. While one face demonstrated a markedly superhydrophilic nature, the other exhibited near-hydrophobic properties. Significantly, the differences in morphology were less evident in the case of TTA-DFP-COF-**5** aligning with the trend of fewer disparities between the two faces, as indicated by WCA measurements.

This duality in the behavior of the faces offers distinct advantages: the superhydrophilic face promotes high water permeability, increased resistance to organic fouling compared to traditional polymeric membranes, while facilitating rapid and efficient removal of oil or organic residues from oil-in-water suspensions. The near-hydrophobic face, on the other hand, could potentially promote antifouling properties for inorganic species while ensuring good water permeation.^49^ This approach also opens the door to the development of fabrication protocols for COF membranes with different wettability behaviors on each side, with the near-hydrophobic side having a higher water contact angle. In this case, the other surface could facilitate the use of the membranes in other applications, such as oil purification from water and seawater desalination by membrane distillation, mimicking the properties of traditional polymeric Janus membranes.^50^

We focus our investigation on the dual near-hydrophobic and hydrophilic properties of membranes—their wettability—which is significantly influenced by both chemical composition and surface textures.^51,52^ The nature of functional groups on a solid surface primarily dictates this wettability: polar groups such as COOH, OH, and NH_2_ enhance hydrophilicity, whereas nonpolar groups like CH_3_ and CF_3_ promote hydrophobicity.^53^ Additionally, the micro/nanotexture textures on the membrane’s surface play a crucial role in determining its wettability. This comprehensive study of both chemical and physical factors enables us to better understand the complex interplay between chemistry and surface texture in controlling membrane wettability.

Using a combination of spectroscopic techniques (Raman, ATR-FTIR, and XPS), we investigated the TTA-DFP-COF-**120** membrane face chemical composition. Atomic force microscopy (AFM) was also employed to analyze their textures. This approach was chosen because SEM (as shown in Figure 2a and Figure S13–S17) revealed clear differences in morphology between the two surfaces, especially when exposed to prolonged reaction time .

Figure S31 displays the direct Raman spectra of the vapor and dioxane faces and their corresponding subtraction. The main difference between the VF and DF faces lies in the regions associated with the C–H vibrations of the aromatic ring, the C–H vibrations of a methyl group, and a possible C–N vibration of the imine group. However, the difference between these two faces was not easy to discern because the penetration depth of the excitation laser, even after adjusting the focal length, could limit the distinction between the two faces.

**Figure 3:**
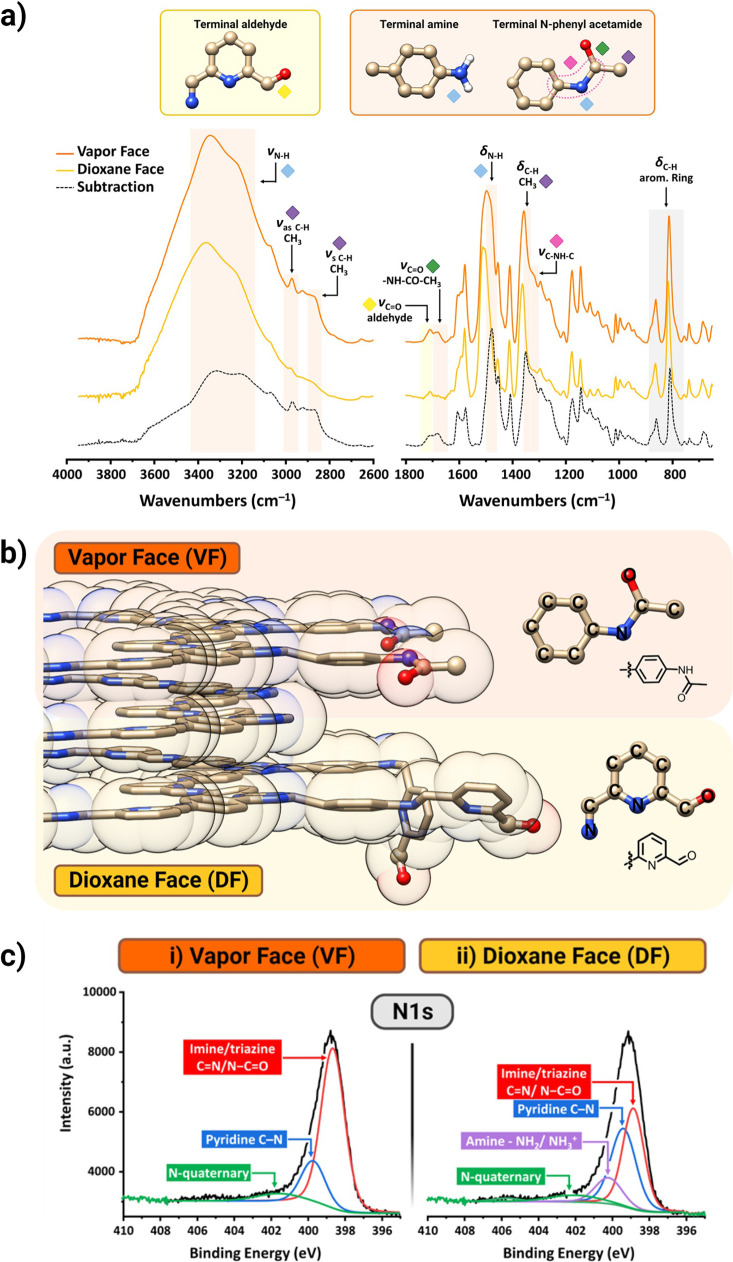
The dual hydrophobic and hydrophilic behavior of the TTA-DFP-COF membrane is mainly due to the favorable orientation of the hydrophilic NH_2_/ NH–CO–CH_3_ groups toward the vapor face and the aldehyde toward the dioxane face. (a) ATR-FTIR spectra of VF (hydrophilic, orange) and DF (nearly hydrophobic, yellow) of the TTA-DFP-COF membrane and their corresponding subtraction (dashed black). (b) Schematic representation of the species distribution within the vapor (VF) and dioxane (DF) face of the membrane. (c) XPS N 1s spectra of VF (i) and DF (ii) of the TTA-DFP-COF membrane.

Consequently, attenuated total reflection Fourier transform IR (ATR-FTIR) spectroscopy was used as an alternative as the penetration depth of the evanescent IR beam is typically on the order of a few micrometers (around 3 μm), making ATR particularly suitable for the qualitative analysis of surface layers of TTA-DFP-COF membrane. Consequently, the analysis is not exclusively confined to the external surface of both faces but part of the bulk as well. This justifies the similar positions of some bands on both sides, however, with different relative intensities (Figure 3a).

The ATR-FTIR spectra of the VF and DF and their corresponding subtraction are given in Figure 3a and the assignment of the corresponding bands in Table S3. The spectrum of the VF reveals distinct higher absorption bands at 3400–3200 cm^**–**1^ (N–H stretching), 2977 and 2868 cm^**–**1^ (C–H asymmetric and symmetric stretching of the CH_3_ group, respectively), 1680 cm^**–**1^ (C=O stretching of −NH–CO–CH_3_), 1480 cm^**–**1^ (N–H bending), 1350 cm^**–**1^ (C–H bending), and 1325 cm^**–**1^ (C–N–C stretching). These bands provide evidence of terminal *N*-phenyl acetamide (phenyl–NH–CO–CH_3_) groups. These groups are most likely formed by a reaction between certain terminal −NH_2_ groups of TTA precursor and aqueous acetic acid (CH_3_COOH). This allows us to propose that the terminal group of the VF is mainly −NH_2_ and amide TTA precursor, preferentially interacting with the aqueous phase (Figure 3b). In contrast, these groups are either absent or present with significantly low intensity in the FTIR spectrum of the DF. Instead, the predominant feature observed on the DF of the TTA-DFP-COF membrane is the presence of terminal aldehyde groups, in which a distinctive band can be identified at 1710 cm^**–**1^. The terminal aldehyde groups from DFP molecules predominantly interact with the dioxane solvent (Figure 3b). This distinction in terminal groups on the membrane’s faces explains the TTA-DFP-COF membrane’s dual hydrophobic and hydrophilic properties, with the NH_2_/ NH–CO–CH_3_ groups being notably more hydrophilic than the aldehyde groups.

To understand how the NH_2_ groups of TTA react with acetic acid, a control experiment mirroring the membrane synthesis conditions was performed, aiming to produce *N*-phenyl acetamide. The formation of this compound was verified through ^1^H NMR, ^13^C CP/MAS solid-state NMR, and FTIR spectroscopy, revealing peaks indicative of amide bond creation (Section 3.3 in the SI). These results confirm the reaction pathway where TTA’s primary amine groups bond with acetic acid to form amide linkages.

The high-resolution XPS survey spectra confirm that the TTA-DFP-COF membrane’s faces are predominantly composed of carbon, nitrogen, and oxygen elements, with the vapor face (VF) showing a greater oxygen content at 6.7%, compared to 3.6% on the Dioxane face (DF) as detailed in Figure S32 and Table S4. Analysis of the C 1s signal reveals four distinct carbon environments corresponding to C=C (approximately 285.0 eV), C–N (approximately 285.8 eV), C–N=C/N–C=O amide (approximately 286.9 eV), and C=O from terminal aldehyde (approximately 288.5 eV) (Figure S33). Notably, the aldehyde C=O is more prevalent in the DF at 5.0%, in contrast to 3.6% in the VF, indicative of terminal aldehyde presence as listed in Table S5. Moreover, the amide’s N–C=O signal is significantly stronger in the VF at 21.4%, versus 16.4% in the DF, suggesting a more substantial interaction and subsequent amide formation on the VF due to the reaction of terminal −NH_2_ groups from the TTA precursor with aqueous acetic acid. The N 1s and O 1s peaks show clear differences between the two faces. The DF’s N 1s spectrum can be deconvoluted into four peaks that correspond to imine/triazine C=N/N–C=O (∼399.1 eV), pyridine C–N (∼399.9 eV), amine −NH_2_/ NH_3_^+^ (∼400.6 eV), and N-quaternary (pronated pyridine, ∼402.5 eV),^54^ as shown in Figure 3c, Figure S33, and Table S6. In contrast, the VF lacks amine species while exhibiting an increased presence of imine/triazine and amide structures, accounting for 50.0% of the N 1s signal, which is an increase from the 37.5% observed on the DF. This discrepancy supports the formation of amide linkages on the VF, as shown in Figure S33 and Table S6.

The DF’s O 1s signal separates into two peaks related to the −C=O of −NH–CO–CH_3_ and the −C=O of the terminal aldehyde groups in the DFP molecules, accounting for 57.4 and 38.2%, respectively. On the VF, the dominance of −NH–CO–CH_3_ at 85.2% confirms the hypothesis of a condensation reaction between the TTA’s terminal −NH_2_ groups and aqueous acetic acid, while the lower presence of −C=O from DFP at 14.8% indicates a selective orientation toward the dioxane phase, as further detailed in Figure S33 and Table S7.

We then performed surface ζ-potential measurements on both faces at neutral pH since all characterization and testing of the membrane were conducted at pH ≈ 7. As expected, the VF showed a significantly more negative ζ-potential (−28 mV) than the DF (−10 mV). This difference can be attributed to the higher presence of terminal *N*-phenyl acetamide (phenyl–NH–CO–CH_3_) groups on the VF. Amides, with a high p*K*_a_ (around 15 or more), are weak bases and unlikely to be protonated under neutral conditions. Instead, their polar C=O bonds contribute to a net negative charge on the VF, amplified by the density of *N*-phenyl acetamide groups and potential hydrogen bonding. In contrast, aldehydes lack basicity and significant charge contribution, resulting in a less negative ζ-potential on the DF due to their weaker dipolar effects (Figure S34). The density of these *N*-phenyl acetamide groups as well as other possible molecular interactions, such as hydrogen bonding on the VF, could also play a role in this observed discrepancy.

The XPS and ζ-potential results agree with the data of the ATR-FTIR analysis. The terminal aldehyde groups tend to orient toward the DF, while the residual NH_2_ groups of TTA react predominantly with COOH from acetic acid the vapor face of the membrane (Figure 3b). The resulting condensation elucidates the dual hydrophobic and hydrophilic characteristics of the TTA-DFP-COF membrane from the chemical distribution aspect.

**Figure 4:**
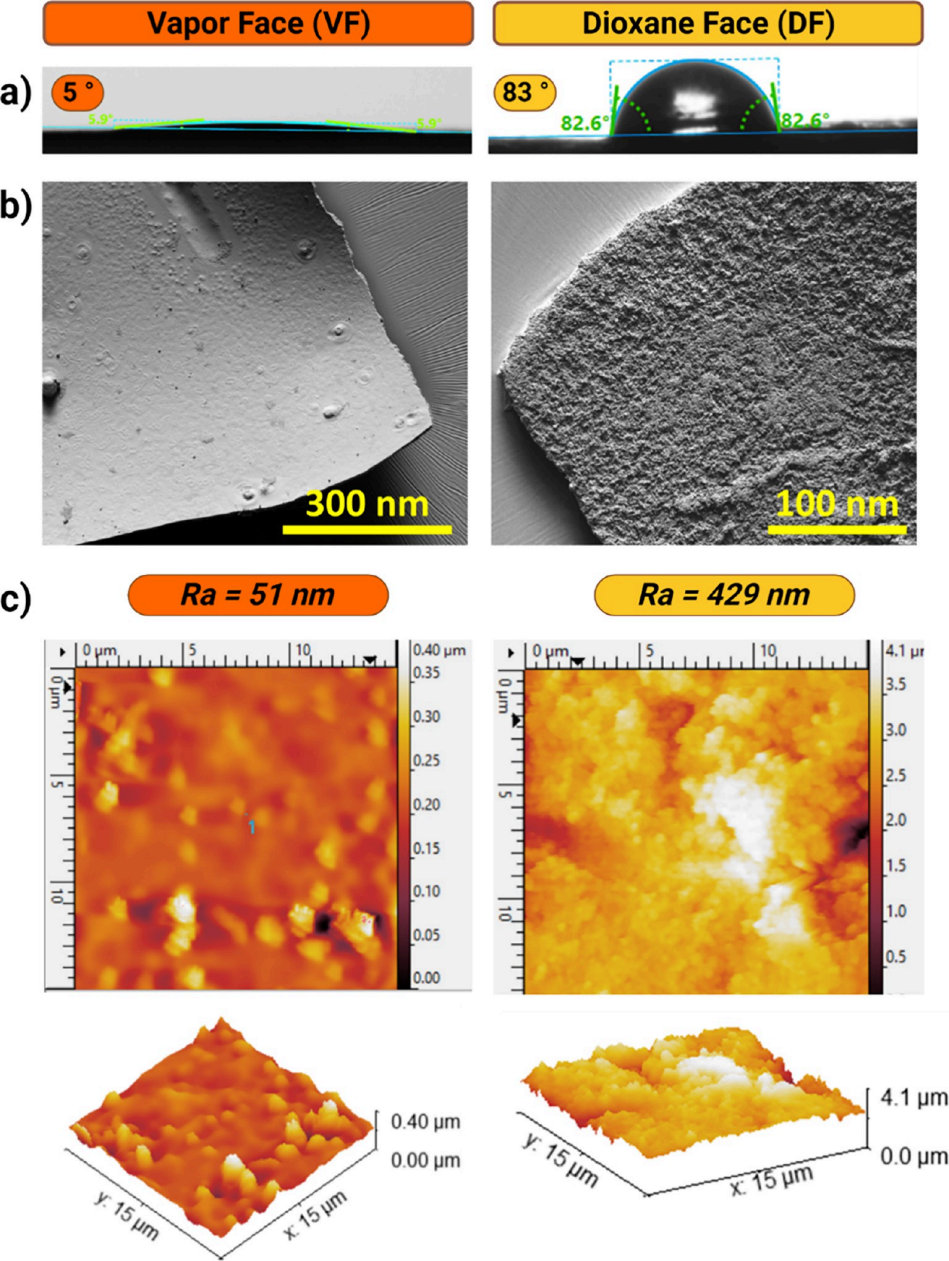
The increased roughness observed on the DF of the TTA-DFP-COF membrane amplifies the effects of its inherent surface chemistry. (a) Digital images of the water contact angle, (b) SEM, and (c) AFM images of VF (left panel, orange) and DF (right panel, yellow) of the TTA-DFP-COF membrane. *Ra* = roughness average.

In 1936, Wenzel^55^ established the correlation between roughness and wettability, stating that introducing surface roughness would amplify the wettability induced by the surface chemistry.^52,53^ The dioxane face (DF) of the membrane is nearly hydrophobic and appeared rough upon visual inspection and closer SEM examination (Figure 4 a,b, and Figures S13–S17). In contrast, as mentioned previously, the superhydrophilic VF displayed a relatively smoother surface. Therefore, the surface morphologies of the membrane’s vapor and dioxane faces were examined by AFM, enabling us to quantify their surface roughness factor, denoted to as R*a* (roughness average). The nearly hydrophilic VF exhibits a comparatively uniform surface with minimal folds and irregularities, as indicated by an average surface roughness value of R*a* = 51 nm (Figure 4c). As a result, the VF showed a mirror-like shiny surface. On the other hand, the hydrophobic DF shows significant fluctuations with an average surface roughness of R*a* = 429 nm. The roughness factor between the two sides is almost ten times higher, highlighting a significant difference in surface roughness between faces.

The increased roughness on the DF side of the TTA-DFP-COF membrane dictates its unique surface chemistry, particularly the dominant terminal aldehyde groups, which make it nearly hydrophobic and affect the wettability properties.^52,53^ This difference in wettability between the sides also depends on how the functional groups of the membrane are oriented and react. In particular, the terminal aldehyde groups are primarily located on the DF, whereas the NH_2_ groups of TTA undergo a condensation reaction with COOH from acetic acid on the VF side. Both the micro/nanotextures and the chemistry of the membrane’s surface illustrate the membrane’s ability to exhibit both near-hydrophobic and hydrophilic properties.

**Figure 5:**
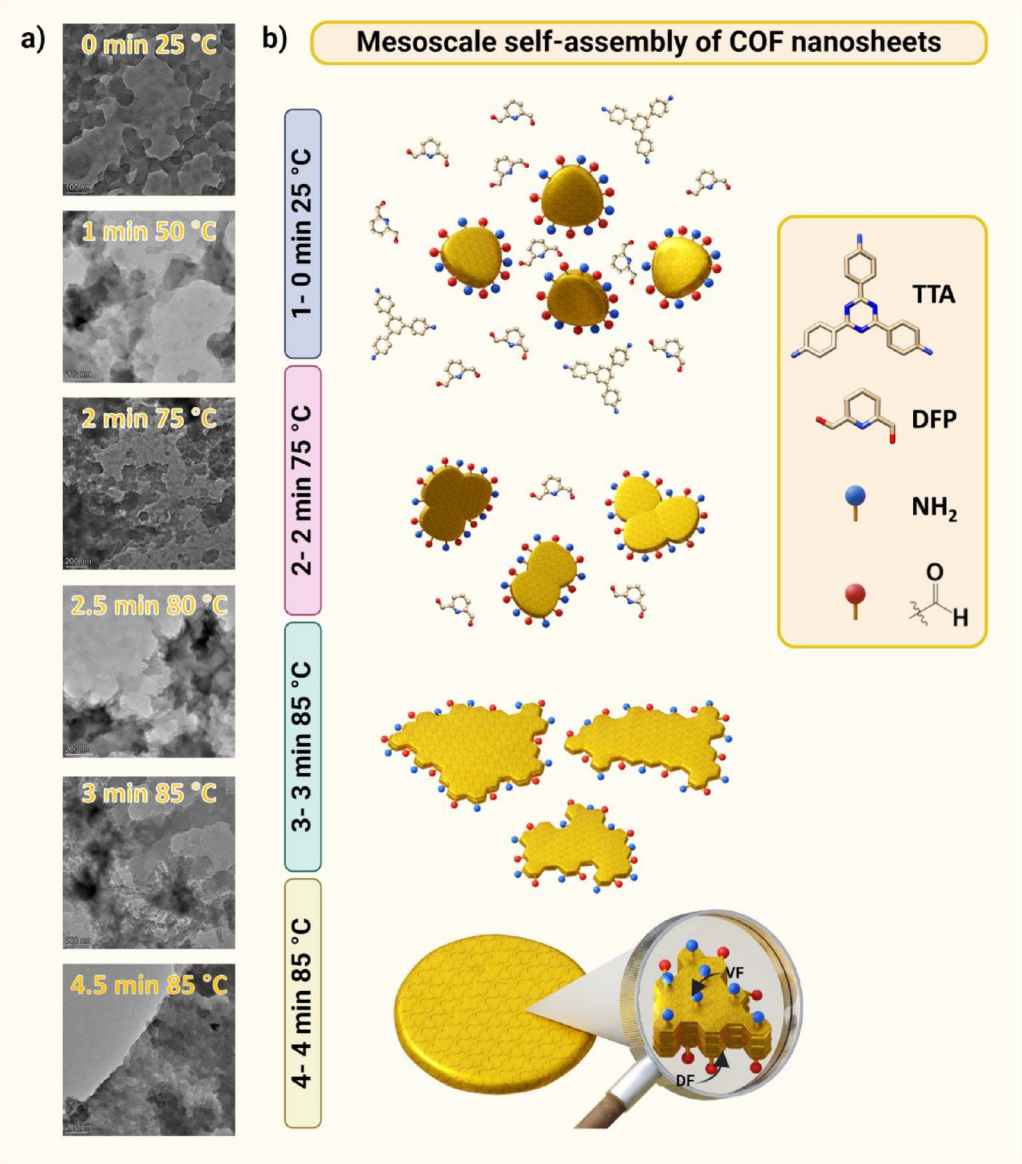
Covalent self-assembly of nanosheets at the interface between dioxane and water vapors transforms them into a self-standing COF membrane. (a) Time-dependent study of the membrane formation visualized by TEM of each step during the synthesis taken at different time intervals. Time is counted after the addition of acetic acid. (b) Illustrative depiction of the mesoscale covalent self-assembly process resulting in a crystalline, porous membrane. The unreacted DFP and terminal aldehyde groups on the nanosheet edges facilitate the assembly through reactions with free amines on the surface. As the COF nanosheets come together at the dioxane-water vapor interface, their peripheral aldehyde and amine groups link via Schiff base reactions. Step 1: 0 min, room temperature—rapid imine condensation produces crystalline COF nanosheets. Step 2: 2 min, 75 °C—mesoscale assembly commences at the interface. Step 3: 3 min, 85 °C—the nanosheets coalesce into expansive branched structures. Step 4: 4.5 min, 85 °C—a robust membrane forms, composed of layered COF sheets, exhibiting a smooth vapor-facing side (VF) and a textured dioxane-facing side (DF), exemplifying the tailored polymerization and morphological development steered by the interface-specific microwave energy application.

To understand how the membrane forms upon microwave activation and the reason for the difference in roughness between faces, we performed a time-dependent TEM/STEM study during the synthesis by immersing TEM grids at the liquid/vapor interface at various time intervals inside the microwave oven and performed a morphological analysis (Figure 5a and Figure S35–S38). The synthesis was performed in an open vessel mode and was slightly modified to slow down the reaction kinetics and simplify the experimental process. To prevent water evaporation as the system was no longer sealed, we reduced the reaction temperature to 85 °C. By decreasing the temperature, we achieved a slower reaction rate without compromising the overall result.

In the reaction, the amine (TTA) is used in default amounts, while the aldehyde (DFP) is in excess. The introduction of aqueous acetic acid ([acetic acid]_final_ = 4.0 M) triggers a rapid imine condensation reaction at room temperature in solution (Figure 5b, step 1). This reaction quickly consumes a considerable amount of TTA, and forms distinct crystalline nanosheets of COF (Figures 5a and S35–38, t = 0 min postacid addition, 25 °C).

The rapid temperature increase induces the mesoscale self-assembly of COF nanosheets, which occurs at the liquid–vapor interface due to two interrelated phenomena: water condensation at the liquid–vapor interface and concentrated microwave energy at the interface (Figures 1c and 5b). The vapor water layer is critical because it allows slow diffusion of acetic acid (demonstrated through ATR-FTIR and XPS) to the interface and the polymerization of free −CHO aldehyde (unreacted DFP) and terminal aldehyde groups and free −NH_2_ amines on the nanosheet surface at the interface between dioxane and water vapors (Figure 5b, step 2).

As a result, the nanosheets form larger structures (Figure 5a, step 3 and Figures S35–S38, *t* = 1.5 min postacid addition). Over time, branching occurs between these star-like structures, bringing them closer together. The structure becomes more ordered through a reorganization with improved stacking and increased transparency due to increased π–π stacking (Figure 5a,b and Figure S35–S38, *t* = 2–3 min postacid addition). After about 4 min, a thick membrane forms as the COF layers continue to accumulate (Figure 5b and Figures S35–S38, step 4). This layer-by-layer growth continues until the DFP linker is completely consumed and no more free aldehydes or amines are available. As a result, the VF is smooth due to the continuous reorganization of the COF nanosheets, while the DF is rough and full of aggregates, as observed by SEM attributed to the end of the process.^56^

Through time-dependent TEM/STEM studies and morphological observations, it was found that the use of microwave activation in synthesis promotes the mesoscale self-assembly of COF nanosheets at the liquid–vapor interface. This process, which is influenced by water condensation and microwave energy concentration, leads to the formation of a thick membrane with a smooth vapor face and a rough dioxane face due to different organizational structures at each interface. The smoothness of the VF membrane results from microwave-assisted interfacial self-assembly in the presence of water vapor, which ensures a controlled and uniform polymerization, and leads to a homogeneous and smooth COF layer. In contrast, the DF membrane formed in contact with dioxane undergoes a less consistent polymerization due to the variable distribution of reactants and microwave energy, leading to a rougher texture.^57^ These textural differences have a significant impact on the membrane’s functionality, particularly affecting wettability and separation performance. The change in surface roughness by a factor of 10, in addition to the changes in chemical composition, is the key to the different wettability observed.

In the rest of the study, we focused on the TTA-DFP-COF-**120** membrane (85 μm), which shows excellent crystallinity, high surface area (690 m^2^ g^–1^), the best mechanical properties as well as the largest discrepancy between superhydrophilicity and near-hydrophobicity of the two faces.

Ensuring the physical and chemical stability of the TTA-DFP-COF membrane is crucial to ensure its efficient use in real-world environments. The thermal stability of the COF membrane was measured by thermogravimetric analysis under nitrogen (Figure S39). The TTA-DFP-COF membranes are thermally stable up to 450 °C without apparent weight loss, which meets the requirements of water treatment application.

The chemical stability of the TTA-DFP-COF membrane was evaluated after 24 h of immersion in acidic, neutral, and basic (pH = 5, 7, and 9) aqueous solutions and common organic solvents (ethanol, dioxane, acetone, and dichloromethane, Figures S40–S44). The TTA-DFP-COF membrane exhibited excellent chemical stability as it retained its integrity without any visible delamination or changes in its chemical structure after being immersed in various conditions for 24 h. The absence of significant changes was confirmed by visual inspection (Figure S40), FTIR (Figures S41 and S42), and PXRD (Figures S43 and S44), highlighting the robust chemical stability of the membrane. The high structural stability of the TTA-DFP-COF skeleton is attributed to the stable backbone, which prevents hydrolysis of the imine nitrogen.^58,59^ Due to their exceptional stability, TTA-DFP-COF membranes are well-suited to fulfill the requirements of molecular sieving and oil removal from water.

### Membrane Filtration Performance

#### Efficient Molecular Sieving Effects

To evaluate the molecular weight cutoff of the synthesized TTA-DFP-COF membrane, four anionic dyes with different molecular weights and dimensions (Rose Bengal RB, Methyl Blue MB, Naphthol Blue Black NBB, Methyl Orange MO), and two salts (NaCl and MgCl_2_), were used as pollutants to assess the rejection performance of the membrane (Table S8).

**Figure 6:**
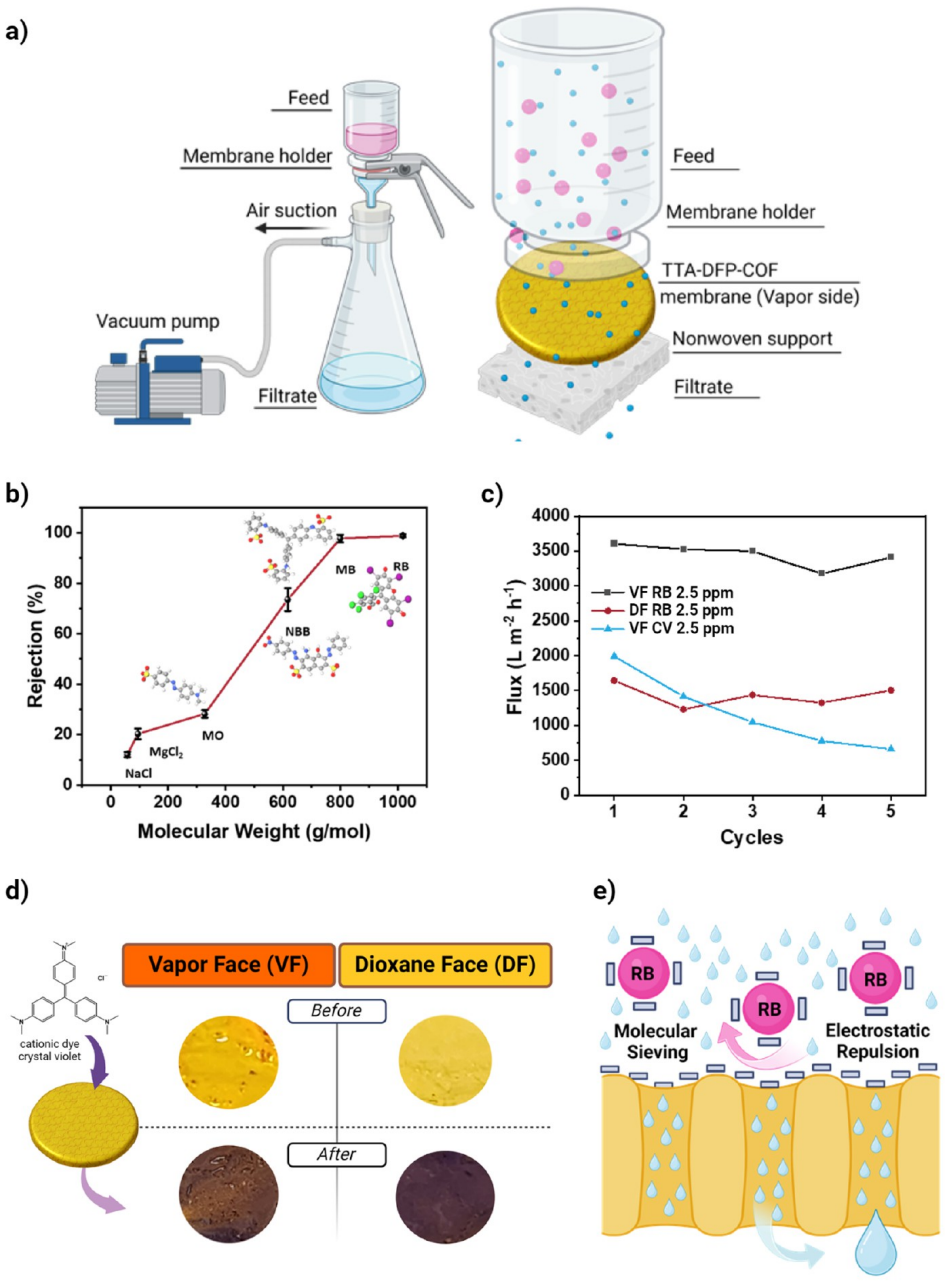
Filtration tests through the TTA-DFP-COF membrane. (a) Filtration assembly, showcasing the vacuum filtration system employed. This system includes a membrane holder comprised of two main parts: the upper section, featuring a cup to contain the feed solution and a support structure to secure the membrane, and the lower section, designed to accommodate the membrane itself, highlighting the specific area where the membrane is positioned. (b) Rejection of salts (NaCl and MgCl_2_, 1000 mg/L) and dyes (Rose Bengal RB, Methyl Blue MB, Naphthol Blue Black NBB, Methyl Orange MO, 2.5 mg/L) with respect to their molecular weight by TTA-DFP-COF membrane. (c) Variation in water flux through the membrane observed over five cycles, with each cycle processing 10 mL of solution. The assessment involved measuring the water flux for a 2.5 mg/L RB solution filtered through both the VF (black) and DF (red) faces of the membrane and for a 2.5 mg/L Crystal Violet (CV) solution filtered through the VF face (blue) of the membrane. (d) Photographs of the membrane’s VF and DF surfaces before and after the 5 cycles of filtration of Crystal Violet (CV), showing the color change caused by CV adsorption on the membrane surface. (e) Proposed mechanism for the rejection of anionic dyes, with RB as an example. The scheme highlights the role of molecular sieving and electrostatic repulsion in the rejection of RB.

After the TTA-DFP-COF membrane was securely placed in the filter holder on top of nonwoven support, the dye and salt solutions were added to the filter holder cup (Figure 6a and Figure S45). In all filtration experiments, the superhydrophilic vapor face of the membrane, which serves as the active layer, was oriented toward the feed solution. This face was chosen for its superior wettability, which increases the water filtration rate, and for its greater negative charge, which improves dye rejection by electrostatic repulsion with anionic dyes. A vacuum pump was used to facilitate the passage of the feed solution through the membrane and the average transmembrane pressure was 0.5 bar. DI water was used to clean the membrane between water filtration runs. Further details of the filtration experiments can be found in the SI.

The results of the filtration tests are shown in Figure 6b. In agreement with the calculated pore size, the membrane effectively achieved almost complete rejection of larger dyes, including Rose Bengal (RB, 11.2 × 12.4 Å) and Methyl Blue (MB, 21.3 × 16.8 Å). In contrast, smaller dyes such as Naphthol Blue Black (NBB, 14.6 × 7.9 Å) and Methyl Orange (MO, 12.1 × 2.4 Å) were partially rejected from the dye solutions (2.5 mg/L), highlighting the selective filtration ability of the membrane.

To evaluate the effectiveness of the membrane in rejecting Rose Bengal (RB) at high concentrations, a solution containing 20 mg/L RB was tested, and a constant rejection rate of about 75% was observed. This shows that the membrane still performs well even at dye concentrations in the range found in industrial wastewater.

Using the self-standing TTA-DFP-COF membrane, a salt rejection of 12% for NaCl and 20% for MgCl_2_ was achieved, indicating effective salt permeability. These results highlight the promising capabilities of the TTA-DFP-COF membrane in selective dye/salt separation processes.^60^

Numerous factors can influence the filtration performance of a membrane, including the aperture size of the membrane, the size of the solute, the surface charge of the membrane, the interaction between the solute and the solvent, and the interaction between the solute and the membrane. In aqueous solutions, the dyes studied are anionic (RB, MB, NBB, MO). Thus, depending on the surface charge of the membrane, the charge of the dye could have affected its rejection. Since the hydrophilic vapor face used for filtration is negatively charged (−28 mV), as shown in Figure S34, electrostatic repulsion could have played a role in the rejection of the negatively charged dyes. However, all the studied dyes were anionic, but only the dyes with larger molecular dimensions, especially RB and MB, were completely rejected. Moreover, the molecular sizes of the dye molecules and their short-end kinetic diameters, are shown in Figure 6b and Table S8. The pore size calculated for the self-standing membrane COF was around 10.2 Å. Interestingly, the RB dye molecule with a long-end kinetic diameter of 12.4 Å was fully rejected, while the smaller dye molecules and salts were able to partially pass through the pores of the membrane. This indicates that size-sieving and shape-selective mechanisms significantly influence the filtration process compared to electrostatic repulsion. The NBB and RB dyes have identical charge densities (−2, Table S8), but their size and molecular weight differ significantly. The higher rejection efficiency for RB than NBB confirms the size-selective nature of the membrane. However, this does not prove that the pores of the COF are the main channels for water filtration but that the pore size of the filtration channels of the membrane is in the order of the calculated pore size, which allows the removal of pollutants with larger sizes.

Furthermore, the negatively charged nature of these dyes suggests that they are more likely to be rejected than adsorbed on the negatively charged membrane surface. Indeed, when the TTA-DFP-COF membrane was immersed in a positively charged dye solution (rhodamine B) for a few minutes, the dioxane face of the membrane showed the dye’s coloration, while the vapor face retained its characteristic yellow color (Movie S7). To better understand the rejection mechanism of the membrane, filtration tests were performed with a cationic dye, crystal violet (CV), whose molecular size (14 × 14 Å) is larger than that of RB (11.2 × 12.4 Å). The results are shown in Figure 6c. Interestingly, the water filtration rate decreased rapidly with time until it was around 660 L m^–2^ h^–1^ after the filtration of 50 mL, compared to 1990 L m^–2^ h^–1^ at the beginning of the test. After assessing the membrane following the filtration tests, a clear color change is observed on both sides of the membrane (Figure 6d), indicating that the cationic dye is adsorbed by the membrane. The decrease in water flux in this case is, therefore, due to the adsorption of the cationic dye on the membrane surface, which blocks the pores of the membrane. This is to be expected due to the negatively charged surfaces of the membrane, which is less suitable for the filtration of cationic dyes due to their adsorption on the membrane surface. However, in the anionic dye filtration experiments using RB, neither staining on either face of the membrane nor a significant decrease in the water filtration rate (Figure 6c) was observed. This test was thus used as further evidence that the membrane does not adsorb anionic dyes during the filtration tests, and thus no additional studies were conducted on CV filtration using the membrane.

Furthermore, we evaluate the water flux through the nearly hydrophobic (DF) face of the membrane. Notably, while the DF face has a similar rejection rate for Rose Bengal (RB) due to its negatively charged surface (−10 mV), it exhibited a lower water permeability, averaging 1428 L m^–2^ h^–1^, in contrast to the 3443 L m^–2^ h^–1^ observed with the superhydrophilic (VF) face (Figure 6c). Nevertheless, the performance far exceeds that of traditional polymeric membranes.^8^ This high water flux indicates that the near-hydrophobic DF face does not hinder water transport and confirms the capabilities of the TTA-DFP-COF self-standing membranes, which offer significantly higher flux rates than conventional options. The remarkable water flux of the TTA-DFP-COF membrane is due not only to its superhydrophilic surface but also to its porosity and the open, interconnected porous structure that minimizes tortuosity. In comparison, conventional polymeric membranes typically used in nanofiltration and reverse osmosis have a water contact angle between 40 and 80° and a denser structure, resulting in lower flux rates.^8^

The proposed filtration mechanism for the rejection of anionic dyes is illustrated in Figure 6e, which highlights the potential role of molecular sieving and electrostatic repulsion in the efficient rejection of RB. This process involves two key strategies: molecular sieving, which allows the passage of water while filtering out larger anionic dye molecules due to the selective size permeability of superhydrophilic surface, and electrostatic repulsion, where the negatively charged surface of the membrane repels anionic dye molecules and prevents their adhesion or penetration. Together, these mechanisms facilitate the efficient separation of dyes from water and ensure thorough dye removal.

#### Water Purification from Mineral Oil

**Figure 7:**
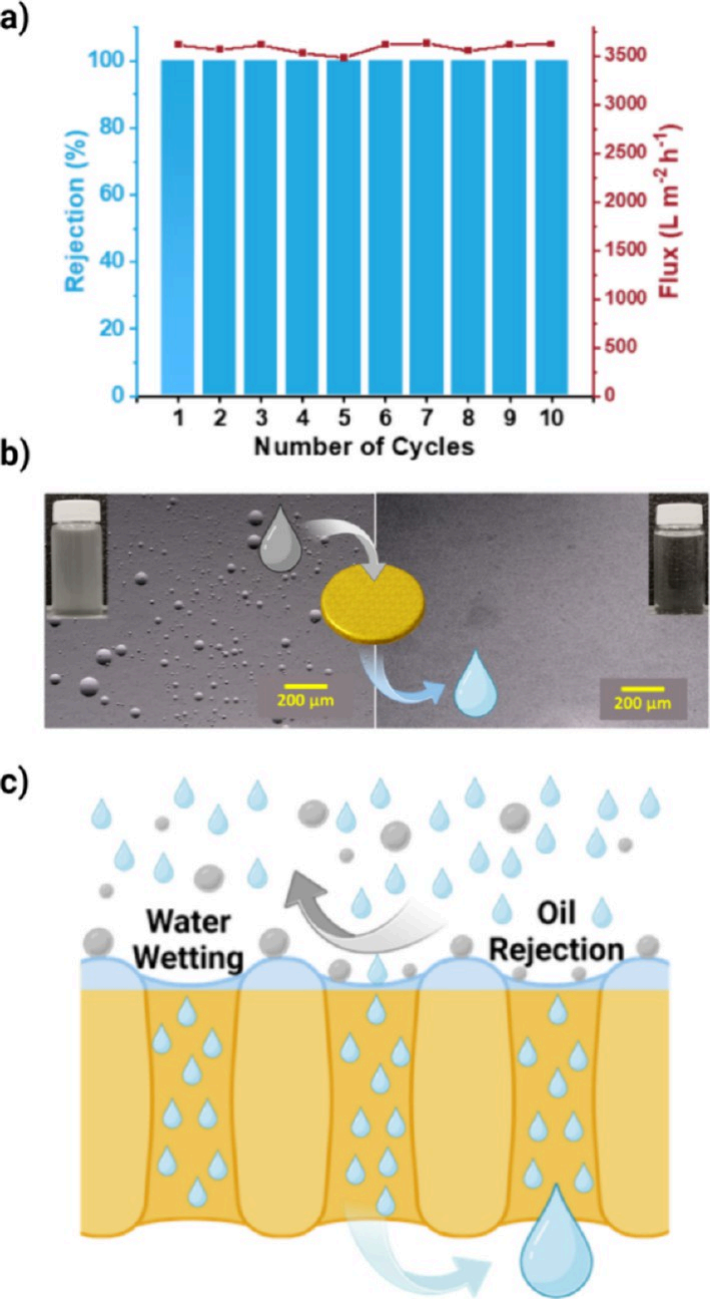
Water Purification from Mineral Oil through the TTA-DFP-COF membrane. (a) Removal of oil from water by TTA-DFP-COF membrane. Left axis (blue): rejection of oil (1000 mg/L) from oil-in-water suspension over 10 cycles. 40 mL of permeate was collected in each cycle. Right axis (red): water flux of the oil-in-water suspension through the membrane over 10 cycles. (b) Microscopic images of the oil-in-water suspension before (left) and after (right) filtration through the TTA-DFP-COF membrane. Photographic images of the suspension before and after filtration are also included as insets in each microscopic image. (c) Proposed mechanism for the rejection of mineral oil facing the superhydrophilic VF of the membrane. The scheme highlights the effect of surface complete wetting by water and how it results in high contact angle with oil droplets, thus rejecting oil.

Leveraging the superhydrophilic vapor face of the TTA-DFP-COF membrane, we tested its performance in water purification from mineral oil (Figure 7a). Remarkably, the TTA-DFP-COF membrane showed an excellent 99% oil rejection from water at a significantly high-water flux of about 3500 L m^–2^ h^–1^. This remarkable performance continued after several filtration cycles, with each cycle efficiently processing around 40 mL of the 1000 ppm oil-in-water emulsion to produce pure water. This not only highlights the efficiency of the membrane in water purification from oil, but also shows the robustness of the membrane, which maintained its performance throughout the tests performed over 10 cycles. Additionally, to test the membrane’s durability, the PXRD of the membrane was recorded after being immersed in water for over two years, and the results show that the membrane retained its crystallinity over time (Figure S46).

The microscope images shown in Figure 7b illustrate the presence of oil droplets within the feed solution on a scale of 200 μm, which represents a clear contrast to the purity of the water permeate after filtration through the COF membrane. Images of the feed and permeate water in glass vials show the transition from a cloudy oil-in-water emulsion to clear, purified water after membrane filtration. In the oil–water separation field, the membrane’s surface wettability is a critical factor. Given the superhydrophilic nature of the membrane’s vapor face, which is used as the active layer for oil rejection, the water phase is expected to spread across the membrane vapor face before infiltrating its porous structure.^61^ This leads to a high contact angle of the oil on the water-wetted membrane vapor-face, which reduces its affinity to the membrane, resulting in its efficient rejection. The proposed mechanism for removing oil from water using the COF membrane is schematically represented in Figure 7c. Table S9 in the SI shows a comparison of the performance of the TTA-DFP-COF membrane reported in this study with recent reports on polymer membranes and composite polymer membranes in oil rejection from oil-in-water emulsions. The TTA-DFP-COF membrane shows superior performance compared to most membranes tested in recent reports, especially in terms of permeation flux, which is in the range of 100–1000 L m^–2^ h^–1^ for most unmodified polymeric membranes.^62−66^

#### Antibacterial Properties and Biocompatibility

In order to assess the resistance of the TTA-DFP-COF membrane to fouling, its antimicrobial properties were studied. *E. coli*, representing Gram-negative bacteria, and *S. aureus*, representing Gram-positive bacteria, were selected to evaluate the membranes’ ability to prevent bacterial adhesion and inhibit their growth.

**Figure 8:**
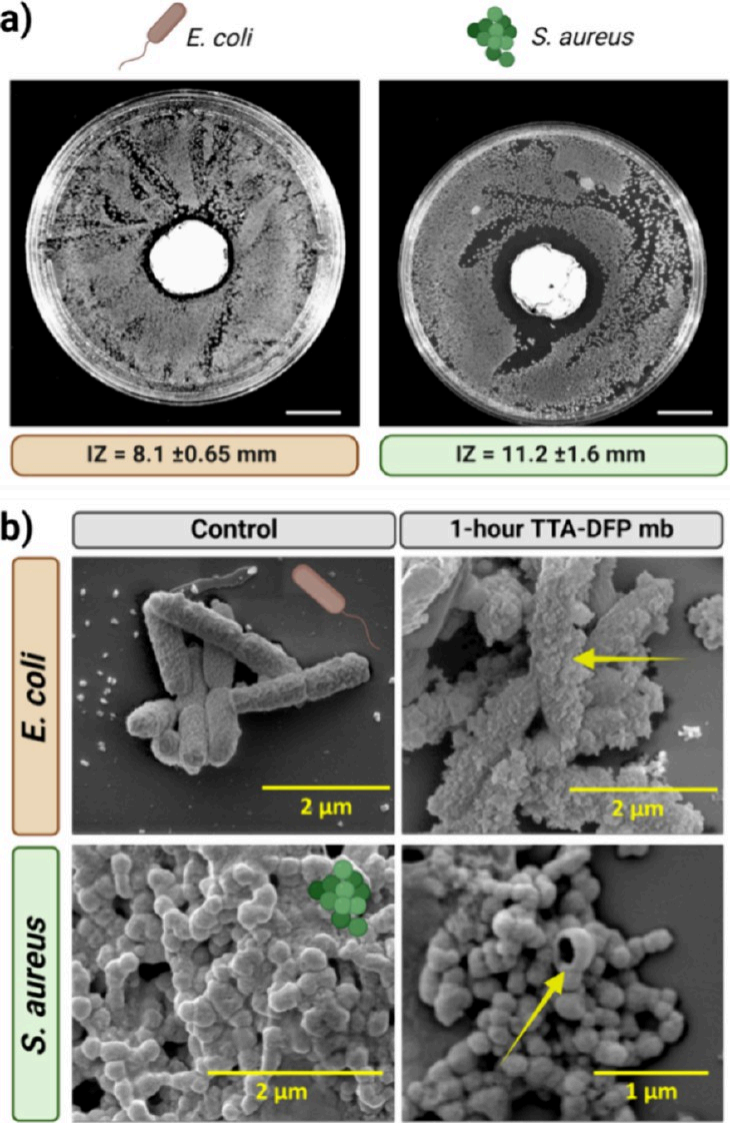
Antimicrobial properties. (a) Inhibition zone (IZ) images of TTA-DFP-COF membrane against bacteria: *E. coli* as a representative Gram-negative model and *S. aureus* as a typical Gram-positive model. (b) Comparative SEM analysis of bacterial morphology before and after 1 h contact with the TTA-DFP-COF membrane. Top panel: *E. coli* bacteria appear intact in control samples but show notable deformation after 1 h exposure to the TTA-DFP-COF membrane, as indicated by the yellow arrow. Bottom panel: *S. aureus* exhibits a similar trend, with control bacteria maintaining their characteristic shape and those in contact with the TTA-DFP-COF membrane displaying significant structural disruptions, highlighted by yellow arrow.

The antimicrobial properties of the TTA-DFP-COF membrane (2.5 cm diameter) were first assessed using the agar disc diffusion technique. The zones of inhibition (IZ) surrounding the areas treated with TTA-DFP-COF against *E. coli* and *S. aureus* were 8.1 ± 0.65 and 11.2 ± 1.6 mm in diameter, respectively, after 24 h, indicating effective antimicrobial activity (Figure 8a and Figure S47).

Subsequently, the TTA-DFP-COF membrane was exposed to suspensions of *E. coli* and *S. aureus* at a concentration of 1 × 10^6^ CFU/mL, maintained at 37 °C and shaken for a 1 h period, resulting in antibacterial efficacies of 83.07 and 78.37%, respectively, highlighting the antibacterial properties of the membrane (Figure S48). Following this exposure, the membrane was washed with PBS and then incubated in PBS at the same temperature for 4 h. Agar plate analyses performed on samples from both the wash solution and the elution solution showed no bacteria, confirming no bacterial adhesion or survival on the membrane (Figure S49). The antibacterial efficacy of the TTA-DFP-COF membrane against *E. coli* and *S. aureus* was demonstrated by morphological changes observed after 1 h exposure by SEM. For *E. coli* and *S. aureus*, the control cells were intact while the cells exposed to the membrane showed damage, including holes and blebs leading to potential content leakage and cell death, indicating broad-spectrum antibacterial activity of the membrane, as seen in Figure 8b and Figure S50.

The TTA-DFP-COF membrane exhibits potent antimicrobial activity by disrupting bacterial cells through electrostatic and hydrogen bond interactions with their membranes. Its efficiency is consistent with previous findings for triazine and imine-based COFs, which rapidly compromise bacterial membranes, prolonging antimicrobial effectiveness and preventing biofilm growth.^67−70^ The TTA-DFP-COF membrane uses a contact-killing strategy for antimicrobial defense, providing a sustainable, nontoxic alternative for water treatment that combines environmental safety with effective bacterial control.^71^

The biocompatibility of the TTA-DFP-COF membrane was validated *in vitro* with HEK-293 cells, a standard for testing material safety in human biology. After incubating the membrane fragments with these cells for 48 h, optical microscopy showed that the cells not only survived but also thrived around and, on the membrane, proving its compatibility (Figure S51). This shows that the membrane is suitable for use in sensitive environments, and does not affect water quality. The TTA-DFP-COF membrane represents an innovative step in water filtration, that combines health, safety, and eco-friendliness. Proven to effectively purify water and combat bacteria while being biocompatible, it stands out as a sustainable choice for solving global water problems.

## Conclusions

In summary, we have developed an approach to rapidly synthesize highly crystalline dual superhydrophilic/near-hydrophobic free-standing COF membranes by using a microwave-mediated self-assembly method at the liquid-water vapor interface. This technique produces COF membranes with exceptional rejection rates, which represents a significant progress in COF membrane synthesis. Our method offers a distinct advantage over previous techniques as it avoids the typically slow diffusion and lengthy amorphous-to-crystalline transformation processes. This efficiency directly addresses the existing challenge of preparing COF membranes suitable for molecular separation in a more straightforward manner. Our approach lays the foundation for the synthesis of high-quality crystalline, free-standing COF membranes.

By tuning the reaction time, we can adjust both the membrane thickness and its wettability characteristics. The TTA-DFP-COF membrane has a unique combination of hydrophilic and near-hydrophobic properties derived from its surface chemistry and micro/nanotexture. The smoother vapor face inherits its hydrophilic nature from the reaction between the terminal −NH_2_ groups of the TTA precursor and aqueous acetic acid, while the rougher dioxane face is enriched with terminal aldehyde groups.

The duality of this membrane enhances its water permeability and makes it superior in organic fouling resistance compared to standard polymeric membranes. Furthermore, it shows exceptional performance in removing oil from oil-in-water mixtures and has a water flux of approximately 3600 L m^–2^ h^–1^. This performance is due to its multilayered structure and consistent porosity. In tests, the TTA-DFP-COF membranes showed outstanding rejection rates for anionic dyes with sizable molecules, along with significant antimicrobial efficacy against bacteria such as *E. coli* and *S. aureus*, while being biocompatible.

Our technology can particularly be suited for small-scale applications. With its unique properties, the TTA-DFP-COF membrane sets a new standard in membrane technology and ideal for use in columnar systems or household water filters. Its simple production, excellent purification performance and antibacterial properties make it an innovative solution for tackling the global water crisis and underline its importance for access to clean water.

## Supporting Information Available

(Movie S1) Membrane formation inside microwave, (Movie S2) vapor vs dioxane faces, (Movie S3) easy peeling process, (Movie 4A–C) membrane formation inside microwave mechanism, (Movie S6) mechanical robustness, (Movie S7) membrane with rhodamine (ZIP)
Supplemental experimental procedures, Figures S1–S51, Tables S1–S10, and supplemental references (PDF)
