# Supplementary material for: Tunable Wettability of a Dual-Faced Covalent Organic Framework Membrane for Enhanced Water Filtration
Source: J Am Chem Soc. 2024 Aug 7;146(33):23537–54. doi: 10.1021/jacs.4c07559 (PMC11345768; doi:10.1021/jacs.4c07559)
Supplement: Supplementary file 2 — ja4c07559_si_002.pdf [file ja4c07559_si_002.pdf]

Supporting Information

Tunable Wettability of a Dual-Faced Covalent Organic Framework Membrane for Enhanced Water Filtration

*Farah Benyettou,[†*1] Asmaa Jrad,[†,1,2] Zineb Matouk,[3] Thirumurugan Prakasam,[1] Houeida Issa Hamoud,[4] Guillaume Clet,[4] Sabu Varghese,[5] Gobinda Das,[1] Mostafa Khair,[5] Sudhir Kumar Sharma,[6] Bikash Garai,[1,2] Rasha G. AbdulHalim,[1] Maryam Alkaabi,[1] Jamaliah Aburabie,[2,6] Sneha Thomas,[5] James Weston,[5] Renu Pasricha,[5] Ramesh Jagannathan,[6] Felipe Gándara,[7] Mohamad El-Roz,[4] Ali Trabolsi*[1,2]*

† equal contribution

1. Chemistry Program, New York University Abu Dhabi (NYUAD), P.O. Box 129188, Abu Dhabi, United Arab Emirates.
2. NYUAD Water Research Center, New York University Abu Dhabi (NYUAD), 129188 Abu Dhabi, United Arab Emirates
3. Technology Innovative Institute, P.O. Box 9639, Abu Dhabi, United Arab Emirates.
4. Normandie Univ, ENSICAEN, UNICAEN, CNRS, LCS, 14000 Caen, France.
5. Core Technology Platform, New York University Abu Dhabi, 129188, Abu Dhabi, United Arab Emirates.
6. Engineering Division, New York University Abu Dhabi, 129188 Abu Dhabi, United Arab Emirates.
7. Instituto de Ciencia de Materiales de Madrid-CSIC, C. Sor Juana Inés de la Cruz 3, 28049 Madrid, Spain

**Correspondence: fb51@nyu.edu*
**Correspondence: at105@nyu.edu*

Table of Content

1. Reagents and techniques 4

2. Synthesis 6

2.1. Synthesis of 2,6-diformylpyridine (DFP) 6

2.2. TTA-DFP-COF membrane synthesis 6

2.3. Control experiment 1: TTA-DFP-COF synthesis in pure acetic acid (no water) 7

2.4. Control experiment 2: TTA-DFP-COF synthesis in the presence of a magnetic stirrer 8

2.5. Control experiment 3: TTA-DFP-COF synthesis using conventional heating methods (oven and an oil bath) 8

2.6. Control experiment 4: TTA-DFP-COF synthesis under microwave irradiation using a maximum power setting of 100 watts. 10

3. Characterizations 11

3.1. Fourier Transform infrared (FTIR) spectroscopy 11

3.2. Solid-state NMR spectroscopy 14

3.3. Mechanism behind the -NH_2 reaction with acetic acid to form the N-phenyl acetamide (phenyl-NH-CO-CH_3) 16

3.4. Scanning electron microscopy (SEM) 21

3.5. Atomic force microscopy (AFM) 26

3.6. Powder X-ray diffraction (PXRD) measurements 28

3.7. High-resolution transmission electron microscopy (HRTEM) 29

3.8. N_2 adsorption-desorption experiments 33

3.9. Mechanical properties by nano-indentation 35

3.10. Water contact angle 37

3.11. Raman spectroscopy 38

3.12. ATR-FTIR spectroscopy 39

3.13. X-ray photoelectron spectroscopy (XPS) 40

3.14. Surface zeta potentials (ζ) measurement 44

3.15. Time-dependent TEM/STEM studies of the membrane formation 45

3.16. Thermogravimetric analysis (TGA) 50

3.17. Stability study 51

4 - Filtration Tests 56

5 - Antibacterial Properties 62

5.1. Disk diffusion 62

5.2. Antibacterial activity 63

5.3. Bacteria attachment experiment 65
5.4. Bacterial morphology study by SEM 66
5.5. Biocompatibility of the TTA-DFP-COF membrane 68
References 70

1. Reagents and techniques

All reagents and starting materials were purchased from Sigma-Aldrich and used without further purification. Deionized water was used from Millipore Gradient Milli-Q water purification system. Thin-layer chromatography (TLC) was performed on silica gel 60 F254 (E. Merck). The plates were inspected under the UV light. Column chromatography was performed on silica gel 60F (Merck 9385, 0.040–0.063 mm). Infrared spectra were recorded on an Agilent Technologies Cary 600 Series FTIR Spectrometer using the ATR mode. The samples' PXRD patterns were recorded using an X-ray Panalytical Empyrean diffractometer. High-resolution transmission electron microscopy (HRTEM) images were obtained using a Talos F200X Scanning/Transmission Electron Microscope (STEM) with a lattice-fringe resolution of 0.14 nm at an accelerating voltage of 200 kV equipped with CETA 16M camera. The high-resolution images of periodic structures were analyzed using TIA software. N_2 adsorption-desorption isotherms were obtained at 77 K using Micrometrics ASAP 2020 surface area analyzer. The topography of the samples was analyzed by dynamic atomic force microscopy (5500 Atomic Force Microscope; Keysight Technologies Inc., Santa Rosa, CA). We acquired topography, phase, and amplitude scans simultaneously. Silicon cantilevers (NanosensorsTM, Neuchatel, Switzerland) with resonant frequencies of 250–300 kHz and force constants of 100– 130 Nm^{-1} were used. The set point value was kept at 2.5V. AFM scans were collected at 1024 points/lines with scan speed of 0.20 at fixed scan angle of 0°. Scan artifacts were minimized by acquiring a typical scan at an angle of 90o under identical image acquisition parameters. We used GwyddionTM free software (version 2.47), an SPM data visualization and analysis tool for post-processing the AFM scans. All solid-state NMR experiments were conducted on a Bruker Avance-HD 600 MHz spectrometer operating at a static field of 14.1 T, resonating at 150.0 MHz for ^{13}C, using a 4.0 mm double resonance MAS probe. Thermogravimetric analysis (TGA) was performed on TA SDT Q600. Scanning electron microscope (SEM) images were obtained from FEI Quanta 450FEG. Surface zeta potentials (ζ) measurement was conducted using ZetaSizer (ZEN3600, Malvern Panalytical, UK).

Table S1. Synthesis conditions of COF self-standing membranes in the literature

COF Name	Synthesis procedure	Synthesis Temperature	Synthesis time	Membrane Thickness	Reference
**TAPA-TFP**<br>**TAPA-TFB**	Interfacial Polymerization (water/DCM interface)	Room temperature	24 h	330 nm	[1]
**COFM**	Steam induction	120 °C	72 h	800 µm	[2]
**TFP–PDA**<br>**TFP–TTA**	Phase switching assembly in the vapor phase	145 °C – 155 °C	12 – 18 h	150 nm	[3]
**COF-membrane**	Interfacial Polymerization (water/oil interface)	Room temperature	48 h	50 nm – 1 µm	[4]
**TAPB-BdPDA**	Interfacial Polymerization (water/DCE interface)	Room temperature	144 h	1.6 – 130 µm	[5]
**COF_{DT} film**	Interfacial Polymerization (liquid/solid interface)	Room temperature	120 h	300 – 500 nm	[6]
**Tam-DbT\ta**	Interfacial Polymerization (Water/ethylacetate interface)	Room temperature	48 h	10 – 20 µm	[7]
**TAPT-TMC**<br>**TAPA-TMC**	Interfacial Polymerization (alkane/ionic liquid interface)	Room Temperature	1 – 30 min	50 – 800 nm	[8]
**COF-300**	Interfacial Polymerization (water/mesitylene interface)	65 °C	24 h	-	[9]
**TTA-DFP-COF**	Microwave-mediated interfacial polymerization (water vapor/dioxane interface)	110 °C	5 min – 2 h	25 – 85 µm	This work

2. Synthesis

2.1. Synthesis of 2,6-diformylpyridine (DFP)

2,6-diformylpyridine (DFP) was synthesized according to the published procedure with no modifications.[10]

2.2. TTA-DFP-COF membrane synthesis

A series of TTA-DFP-COF membranes were prepared by using increasing reaction times. A 30 mL clean glass microwave tube (previously scratched using clean tweezers) was charged with 2,6-diformylpyridine (DFP, 21 mg, 0.15 mmol, 5 equivalents) and 4,4',4''-(1,3,5-triazine-2,4,6-triyl)trianiline (TTA, 12 mg, 0.03 mmol, 1 equivalent), in 3 mL of anhydrous 1,4-dioxane. The mixture was sonicated for 1 min to dissolve the molecular organic building units completely. Then, 0.5 mL of aqueous acetic acid (13 M, $[\text{acetic acid}]_{final}$ = 4.0 M) was added rapidly, and the mixture was placed immediately in the microwave oven and heated under microwave irradiation (300 W) to 110 °C (Figure S1). The thickness of the membrane was controlled by varying the reaction time. The reaction was stopped after 5, 45, and 120 minutes under microwave irradiations. The membrane was denoted as TTA-DFP-COF-**5**, TTA-DFP-COF-**45**, and TTA-DFP-COF-**120**, where 5, 45, and 120 represent the reaction time in minutes. At the end of the chosen reaction time, the free-standing membrane with and no visible cracks could be easily collected with a tweezer and cleaned several times in dioxane first, followed by ethanol and water. The membrane is stored in ethanol or water.

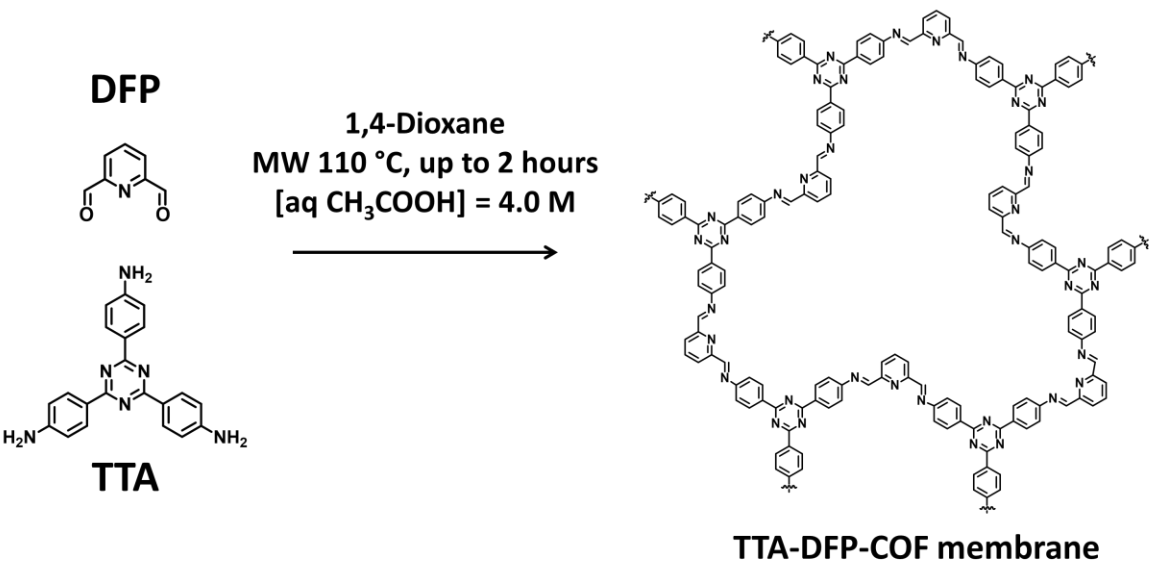

Figure S1. Synthetic route and chemical structure of linkers and TTA-DFP-COF membrane.

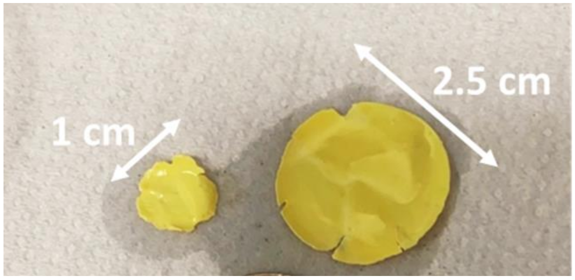

Figure S2. TTA-DFP-COF membrane photograph showing the membrane collected from the microwave reaction vessel and their centimeter-scale size, self-standing nature and flexibility.

2.3. Control experiment 1: TTA-DFP-COF synthesis in pure acetic acid (no water)

TTA-DFP-COF was synthesized by co-condensation of 2,6-diformylpyridine (DFP, 21 mg, 0.15 mmol, 5 equivalents) and 4,4',4''-(1,3,5-triazine-2,4,6-triyl)trianiline (TTA, 12 mg, 0.03 mmol, 1 equivalent), in 3 mL of anhydrous 1,4-dioxane in presence of 0.5 mL of dry acetic acid (17 M, ([acetic acid]$_{final}$ = 5.0 M, no water) sealed in a 10 mL glass microwave tube and heated under microwave irradiation (300 watt) to 110 °C for 120 minutes. No membrane formation was observed.

2.4. Control experiment 2: TTA-DFP-COF synthesis in the presence of a magnetic stirrer

TTA-DFP-COF was synthesized by co-condensation of 2,6-diformylpyridine (DFP, 21 mg, 0.15 mmol, 5 equivalents) and 4,4',4''-(1,3,5-triazine-2,4,6-triyl)trianiline (TTA, 12 mg, 0.03 mmol, 1 equivalent), in 3 mL of anhydrous 1,4-dioxane in presence of 0.5 mL of aqueous acetic acid (13 M, $[\text{acetic acid}]_{final}$ = 4.0 M) sealed in a 10 mL clean glass microwave tube and heated under microwave irradiation (300 watt) to 110 °C for 120 minutes in the presence of magnetic stirring. No membrane formation was observed (Movie 5).

2.5. Control experiment 3: TTA-DFP-COF synthesis using conventional heating methods (oven and an oil bath)

TTA-DFP-COF was synthesized by co-condensation of 2,6-diformylpyridine (DFP, 21 mg, 0.15 mmol, 5 equivalents) and 4,4',4''-(1,3,5-triazine-2,4,6-triyl)trianiline (TTA, 12 mg, 0.03 mmol, 1 equivalent), in 3 mL of anhydrous 1,4-dioxane in presence of 0.5 mL of aqueous acetic acid (13 M, $[\text{acetic acid}]_{final}$ = 4.0 M) sealed in a 10 mL clean glass microwave tube and heated using conventional heating methods (oven and an oil bath) to 110 °C for 120 minutes. No membrane formation was observed (Figure S3).

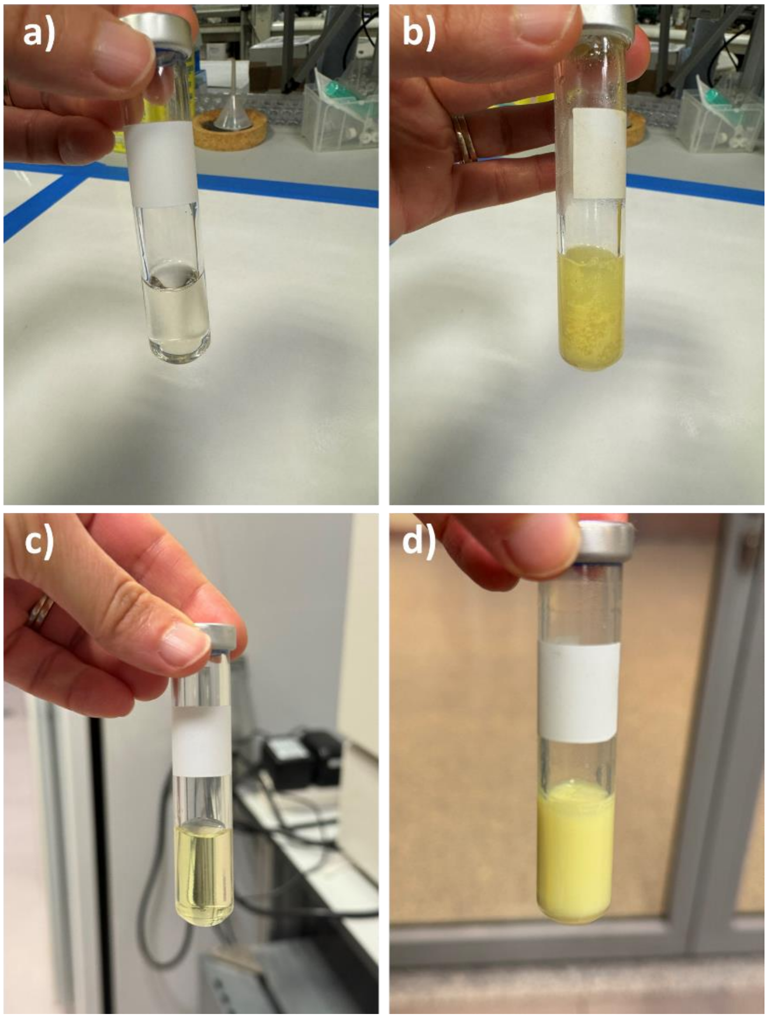

Figure S3. Visual comparison of reaction mixtures before (a, c) and after (b, d) conventional heating methods. Images (a) and (c) depict the vials prior to heating, containing a mixture of 2,6-diformylpyridine (DFP, 21 mg, 0.15 mmol, 5 equivalents) and 4,4',4''-(1,3,5-triazine-2,4,6-triyl)trianiline (TTA, 12 mg, 0.03 mmol, 1 equivalent) in 3 mL of anhydrous 1,4-dioxane with the addition of 0.5 mL of aqueous acetic acid (13 M, resulting in a final concentration of 4.0 M acetic acid). Image (b) shows the vial after heating in an oil bath, and image (d) after heating in an oven, both for 2 hours at 110 °C. In both (b) and (d), no membrane formation is observed, contrasting with the outcomes when microwave heating is applied to the same mixture under similar conditions.

2.6. Control experiment 4: TTA-DFP-COF synthesis under microwave irradiation using a maximum power setting of 100 watts.

TTA-DFP-COF membrane was synthesized by co-condensation of 2,6-diformylpyridine (DFP, 21 mg, 0.15 mmol, 5 equivalents) and 4,4',4''-(1,3,5-triazine-2,4,6-triyl)trianiline (TTA, 12 mg, 0.03 mmol, 1 equivalent), in 3 mL of anhydrous 1,4-dioxane in presence of 0.5 mL of aqueous acetic acid (13 M, $[\text{acetic acid}]_{final}$ = 4.0 M) sealed in a 10 mL clean glass microwave tube and heated under microwave irradiation (100 watt) to 110 °C for 120 minutes. Under this reduced power setting, we successfully synthesized a COF membrane; however, it exhibited less robustness compared to membranes synthesized at 300 watts.

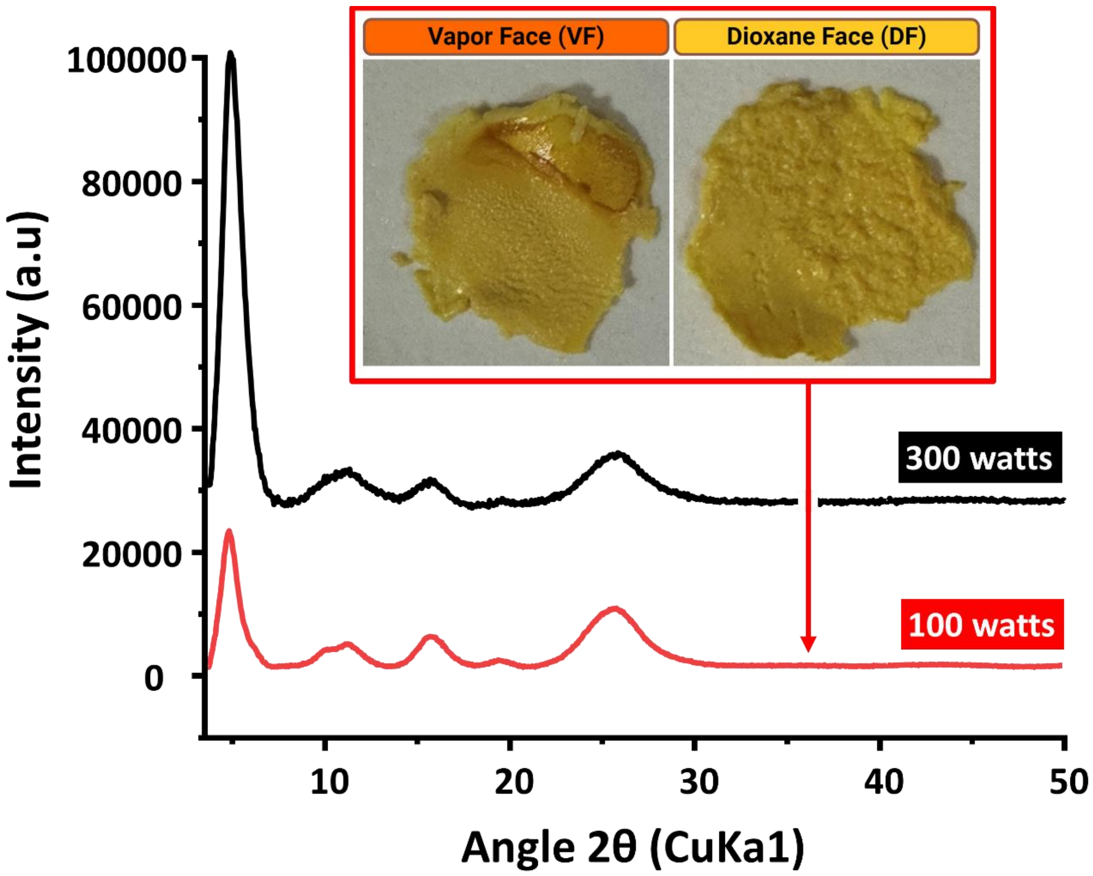

Figure S4. PXRD of TTA-DFP membrane performed under microwave irradiation using a maximum power setting of 300 watts (black) and 100 watts (red). Inset: Pictures of TTA-DFP-COF membrane VF and DF performed under microwave irradiation using a maximum power setting of 100 watts.

3. Characterizations

3.1. Fourier Transform infrared (FTIR) spectroscopy

The TTA-DFP-COF membranes formation was confirmed and characterized by ATR-IR spectroscopy using an Agilent Technologies Cary 600 Series FTIR spectrometer. The spectral data within the range of 4000 to 600 cm^{-1} were recorded, and 512 scans were averaged for each spectrum with a spectral resolution of 2 cm^{-1}. The spectrum of the background was recorded first, and it was subtracted from the spectra of samples automatically.

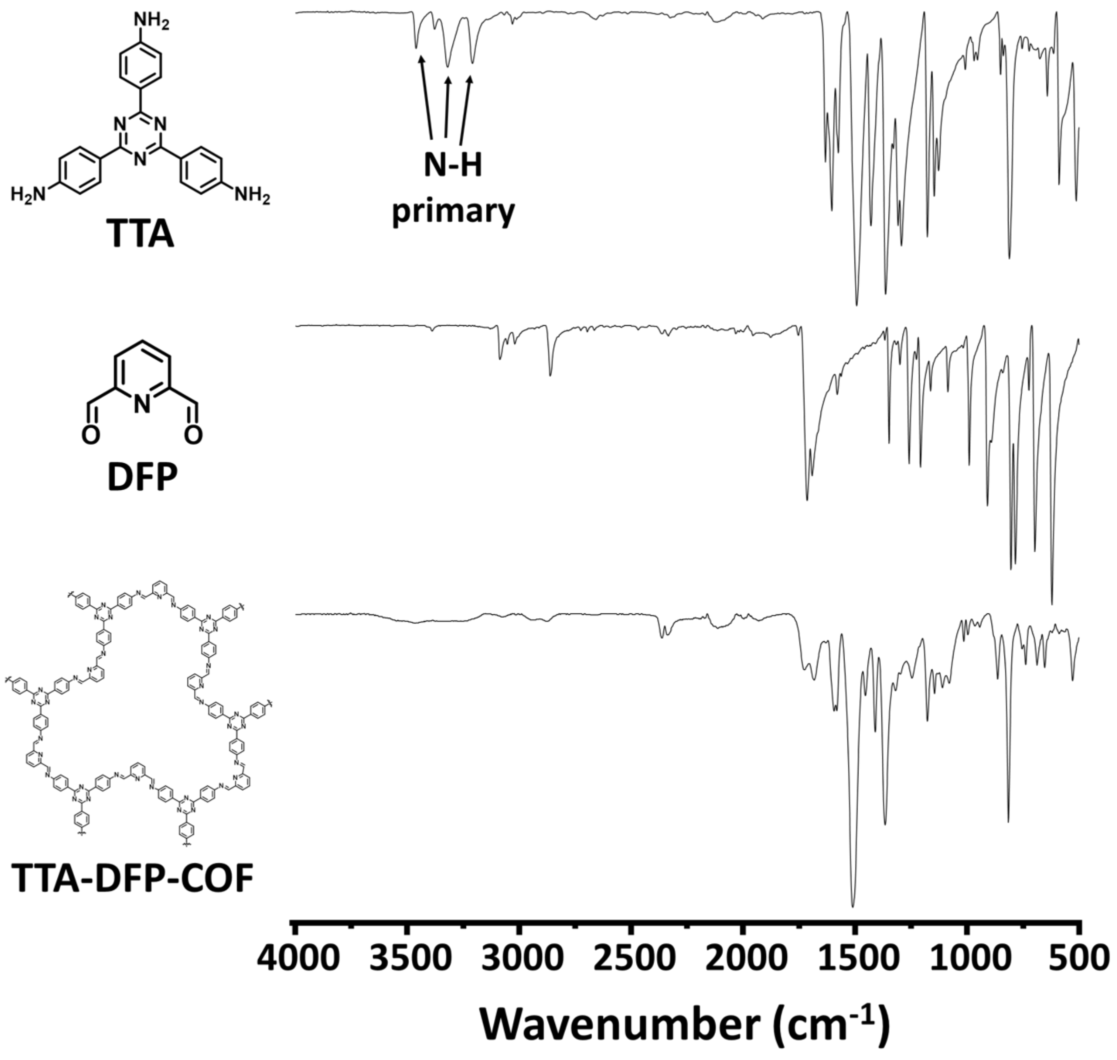

Figure S5. Stacked FTIR spectra of TTA-DFP-COF membrane and its precursors, 2,6-diformylpyridine (DFP) and 4,4',4''-(1,3,5-triazine-2,4,6-triyl)trianiline (TTA).

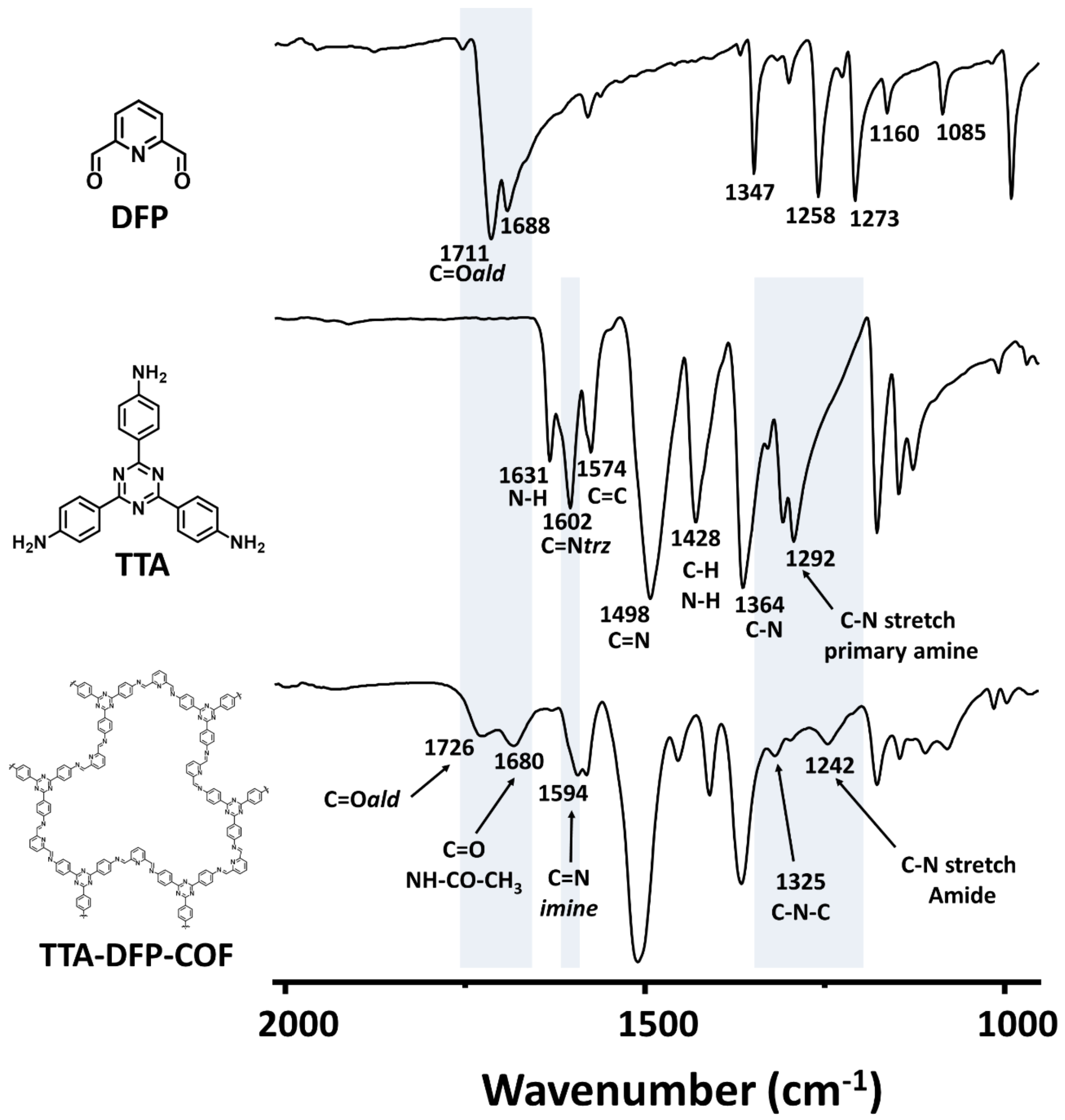

Figure S6. Stacked FTIR spectra of TTA-DFP-COF membrane and its precursors, 2,6-diformylpyridine (DFP) and 4,4',4''-(1,3,5-triazine-2,4,6-triyl)trianiline (TTA) between 2000-1000 cm^{-1}.

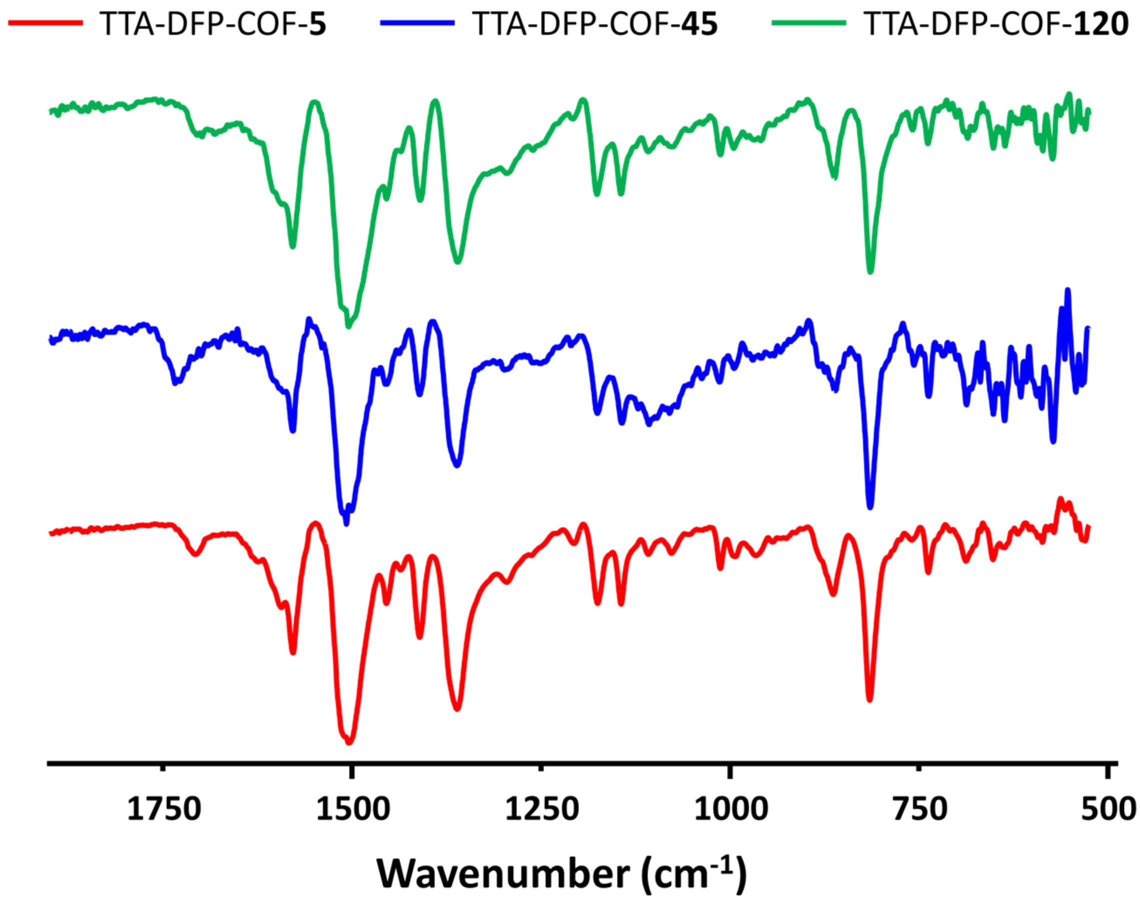

Figure S7. Stacked FTIR spectra of TTA-DFP-COF-**5** (red), TTA-DFP-COF-**45** (blue), and TTA-DFP-COF-**120** (green).

3.2. Solid-state NMR spectroscopy

Magic Angle Spinning (MAS) solid-state NMR experiments were carried out on a Bruker Avance-HD 600 MHz spectrometer operating at a static field of 14.1 T using a 4.0 mm MAS probe. Dry samples were packed into 4.0 mm zirconia rotors and were spun at a MAS frequency of 14 kHz. ^{1}H-^{13}C CP/MAS experiments were performed using a standard linearly ramped cross-polarization pulse sequence. ^{13}C chemical shifts were externally referenced to the adamantane CH_2 signal at 38.48 ppm. NMR data were processed using TopSpin software.

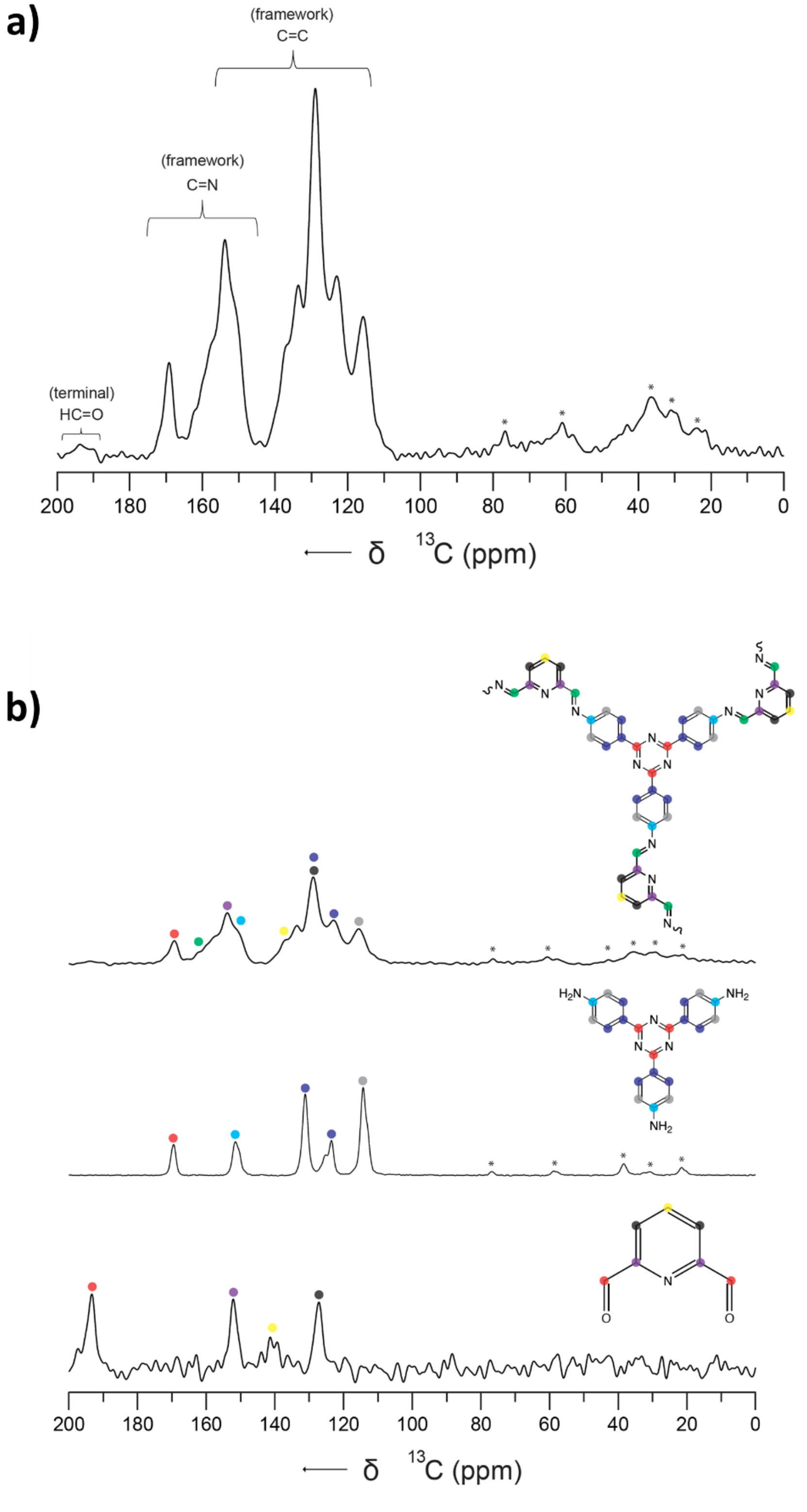

Figure S8. ^{13}C CP/MAS solid-state NMR spectra of a) TTA-DFP-COF membrane and b) TTA-DFP-COF membrane (top), TTA (middle) and DFP (bottom). The asterisks denote spinning sidebands.

3.3. Mechanism behind the $-NH_2$ reaction with acetic acid to form the N-phenyl acetamide (phenyl-NH-CO-CH_3)

To elucidate the reaction mechanism between the $-NH_2$ groups of TTA and acetic acid, we conducted a control experiment where TTA was reacted with acetic acid in the same conditions as the membrane synthesis. These experiments were specifically designed to demonstrate the formation of N-phenyl acetamide (phenyl-NH-CO-CH_3).

We performed the reaction between TTA and acetic acid. 4,4',4''-(1,3,5-triazine-2,4,6-triyl)trianiline (TTA, 12 mg, 0.03 mmol, 1 equivalent) was put in 3 mL of anhydrous 1,4-dioxane in presence of 0.5 mL of acetic acid (13 M, [acetic acid]final = 4.0 M) sealed in a 10 mL clean glass microwave tube and heated under microwave irradiation to 110 °C for 120 minutes. The compound was precipitated using a solution of NaOH (1M) to collect a yellow solid, which was washed with ethyl acetate twice and dried overnight in an oven.

The successful formation of N-phenyl acetamide was confirmed by ^{1}H NMR, ^{13}C Cross-Polarization/Magic Angle Spinning (CP/MAS) solid-state NMR, and Fourier-Transform Infrared (FTIR) spectroscopy analysis, which showed characteristic peaks corresponding to the amide bond formation. This validation serves as a clear indication of the reaction mechanism, where the primary amine groups in TTA react with acetic acid to form amide linkages.

a – ^{1}H NMR spectroscopy

The proton NMR spectrum (Figure S9) illustrates the successful synthesis of N-phenyl acetamide from the reaction of TTA which contains $-NH_2$ groups with acetic acid, based on the chemical shifts and splitting patterns observed. The resonance signal labeled "c" corresponds to the protons of the $-NH_2$ group in TTA that have been acetylated to form the -NH-CO-CH_3 group. In the product, amide -NH protons to couple within N-phenyl appear as a singlet and are shifted upfield (δ: 10.41 ppm) compared amine protons in TTA. The resonance signal labeled "d" represents the methyl group protons (CH_3) of the amide functional group as a singlet (δ: 2.12 ppm). The presence of the singlet of the methyl group supports the conclusion that the reaction

to form N-phenyl acetamide was successful. The resonance signal labeled "b and a" corresponds to the aromatic protons shifted to up field compared to aromatic protons in the TTA.

The absence of signals associated with the starting materials (such as the singlet of NH_2 groups in the free amine) and the presence of the singlet of the methyl group supports the conclusion that the reaction to form N-phenyl acetamide was successful.

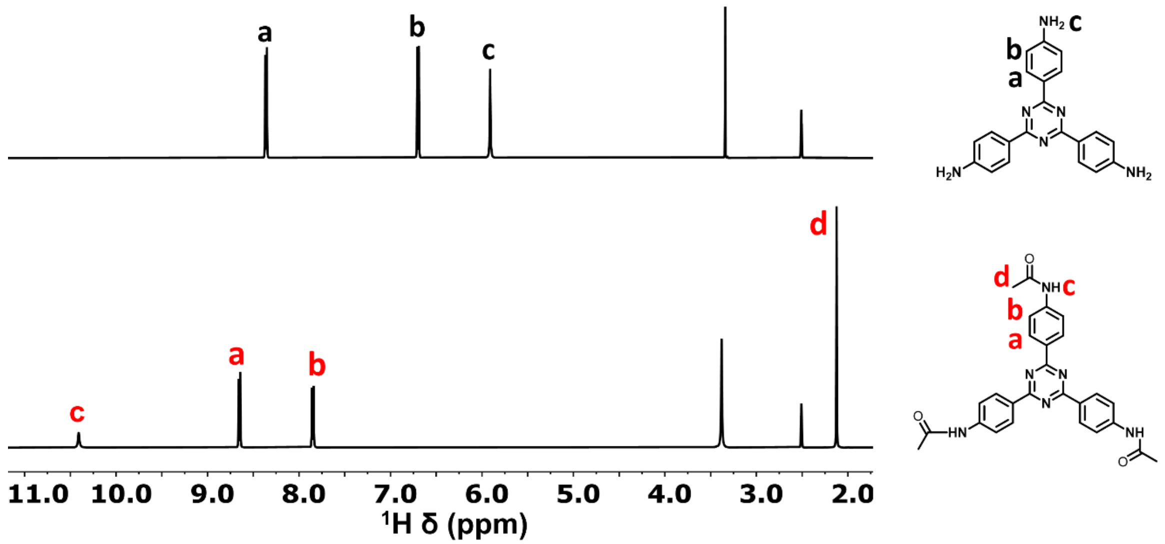

Figure S9. ^1H NMR Spectrum of a) TTA and b) N-Phenyl Acetamide from the reaction between TTA and Acetic Acid.

b - ^{13}C Cross-Polarization/Magic Angle Spinning (CP/MAS) solid-state NMR spectroscopy

The successful formation of N-phenyl acetamide was also confirmed by ^{13}C CP/MAS solid-state NMR, which showed high frequency peaks appearing from the triazine carbons (170.6 ppm), aromatic carbons (110 to 150 ppm) in addition to the new peaks appearing from the carbonyl (164.6 ppm) and methyl groups (25.3 ppm), indicative of the amide bond formation (Figure S10).

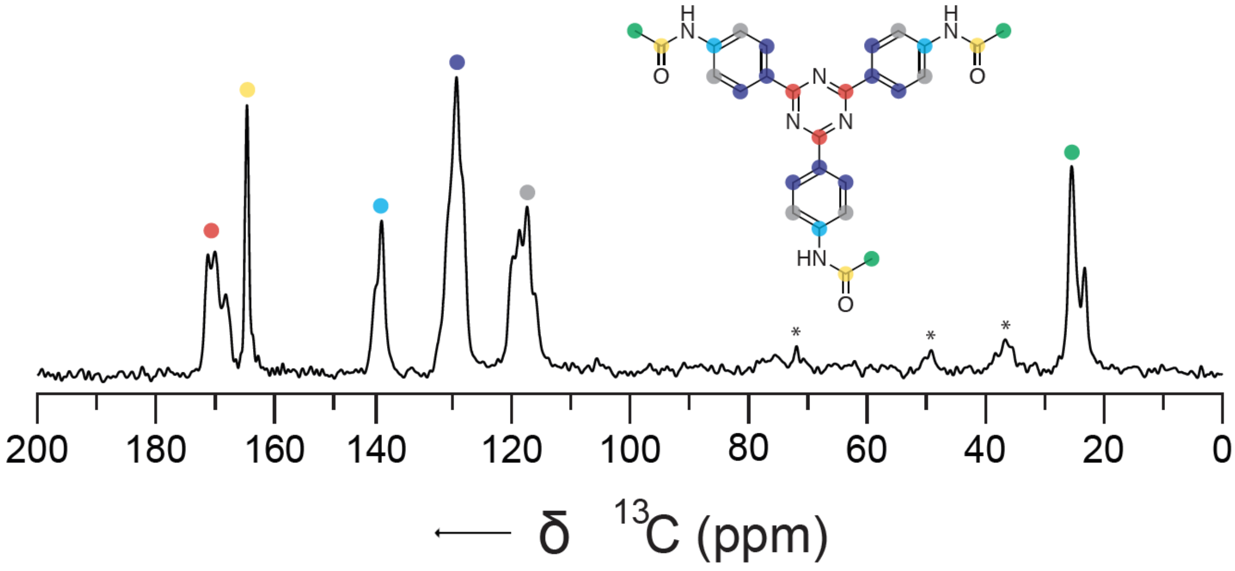

Figure S10. ^{13}C Cross-Polarization/Magic Angle Spinning (CP/MAS) solid-state NMR spectrum confirming N-Phenyl Acetamide formation. The spectrum displays characteristic resonances corresponding to the triazine carbon (red dot), carbonyl carbon (yellow dot), aromatic carbons (blue, cyan and grey dots), and the methyl carbon (green dot) in the amide group, consistent with the chemical structure of N-phenyl acetamide.

b – FTIR spectroscopy

The FTIR spectra of TTA before and after reacting with acetic acid show significant differences due to the chemical changes during the reaction (Figures S11 and S12). Primary Amine (TTA): In TTA, as a primary amine, the FTIR spectrum typically shows N-H stretching vibrations. These are observed as three distinct bands at 3457, 3317, and 3206 cm^{-1}, indicative of the N-H stretching modes as well as at 1631 cm^{-1} indicative of the N-H bending (scissoring) vibrations, in addition the C-N stretch of primary amine can be observed at 1291 cm^{-1}. After Reaction with Acetic Acid (Formation of Amide): C=O Stretch (Amide): A prominent change in the spectrum is the appearance of the C=O stretching vibration of the amide group, observed at 1684 cm^{-1}. This strong band is characteristic of the amide linkage. N-H Stretch (Amide): The N-H stretch in the primary amine, previously observed at 1631 cm^{-1}, is significantly diminished upon reacting with acetic acid due to amide formation. In the FTIR spectrum of the amide, this N-H stretch now

appears as a shoulder at 1622 cm^{-1}. Alteration of C-N Stretch: The C-N stretch of the primary amine, originally at 1291 cm^{-1}, undergoes a significant change as the amine converts to an amide. The resulting spectrum features C-N stretching vibrations characteristic of amides, observed at 1253 cm^{-1}.

The conversion of the primary amine to an amide upon reacting with acetic acid is evidenced in the FTIR spectrum by the emergence of characteristic amide bands, particularly the C=O stretch, and changes in the N-H and C-N stretch vibrations. The diminished N-H stretch intensity of the primary amine, along with new amide-specific bands, verifies the chemical transformation.

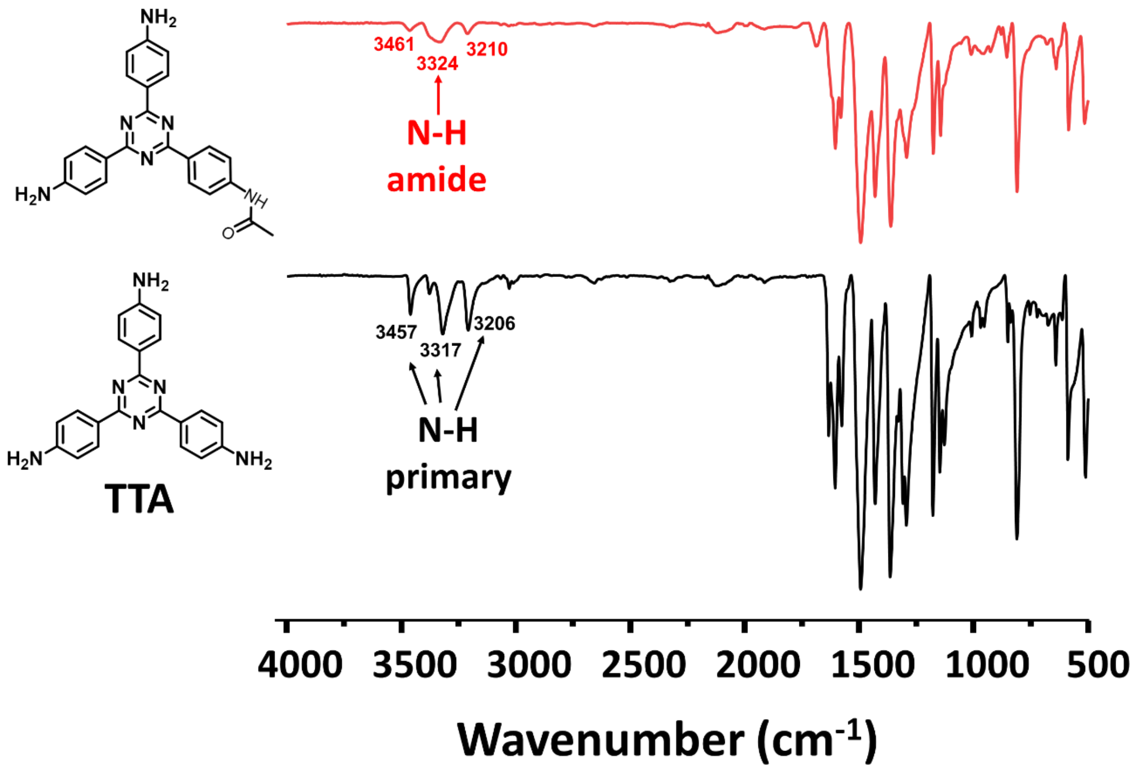

Figure S11. Stacked FTIR spectra of 4,4',4''-(1,3,5-triazine-2,4,6-triyl)trianiline (TTA) and after reaction with acetic acid.

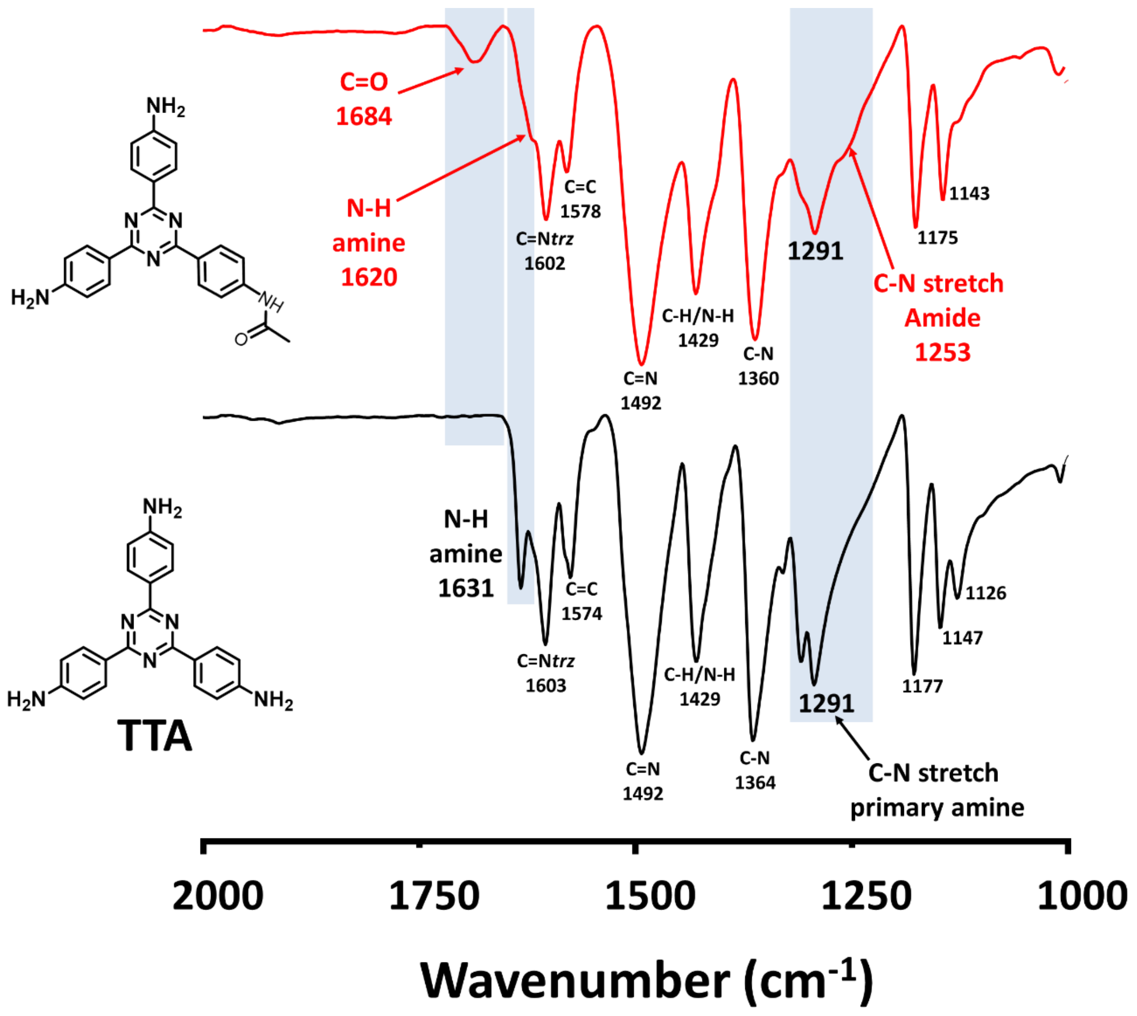

Figure S12. Stacked FTIR spectra of 4,4',4''-(1,3,5-triazine-2,4,6-triyl)trianiline (TTA) after reaction with acetic acid between 2000-1000 cm^{-1}.

3.4. Scanning electron microscopy (SEM)

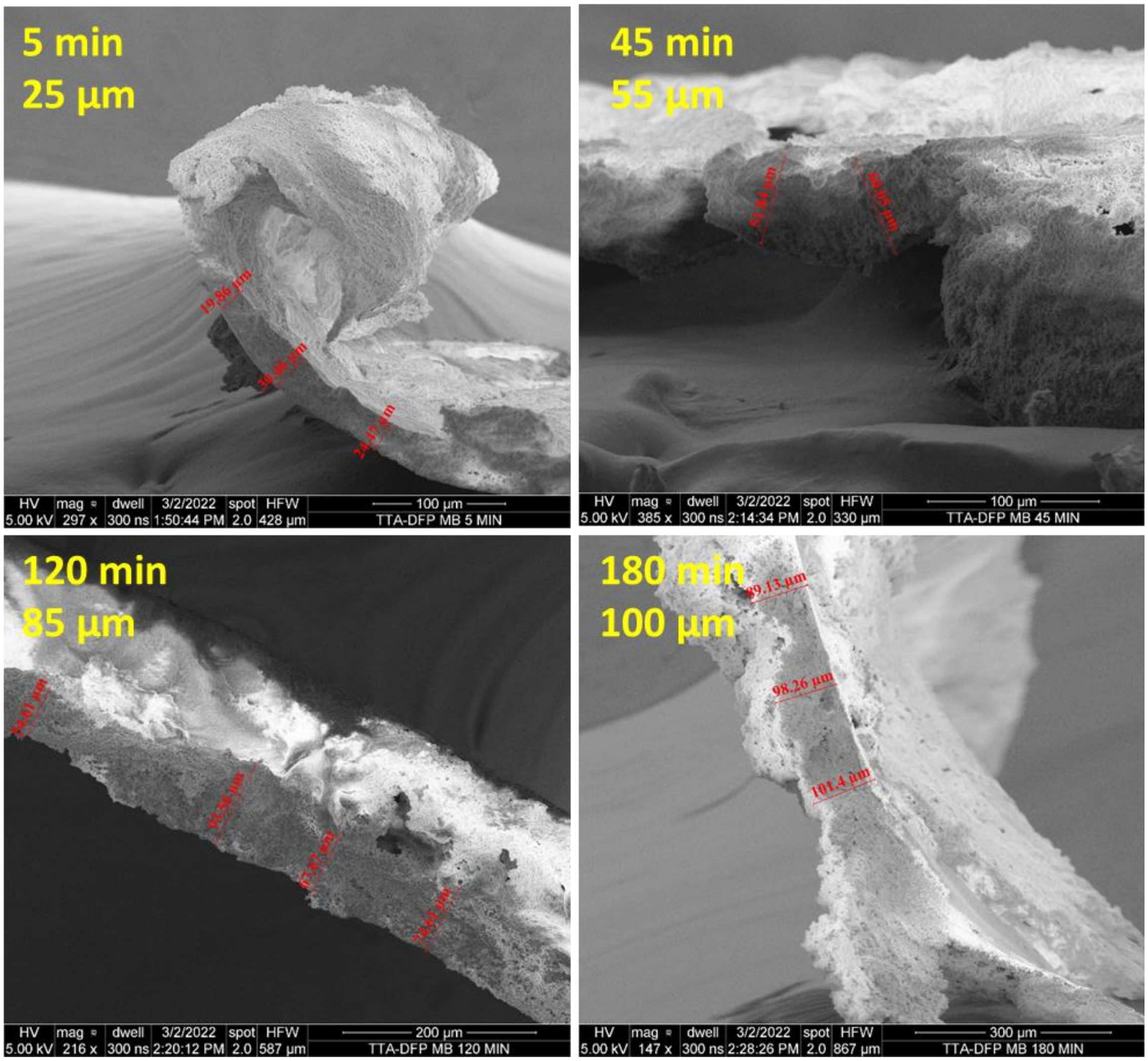

Figure S13. SEM images of TTA-DFP-COF-**5**, TTA-DFP-COF-**45**, TTA-DFP-COF-**120** and TTA-DFP-COF-**180**.

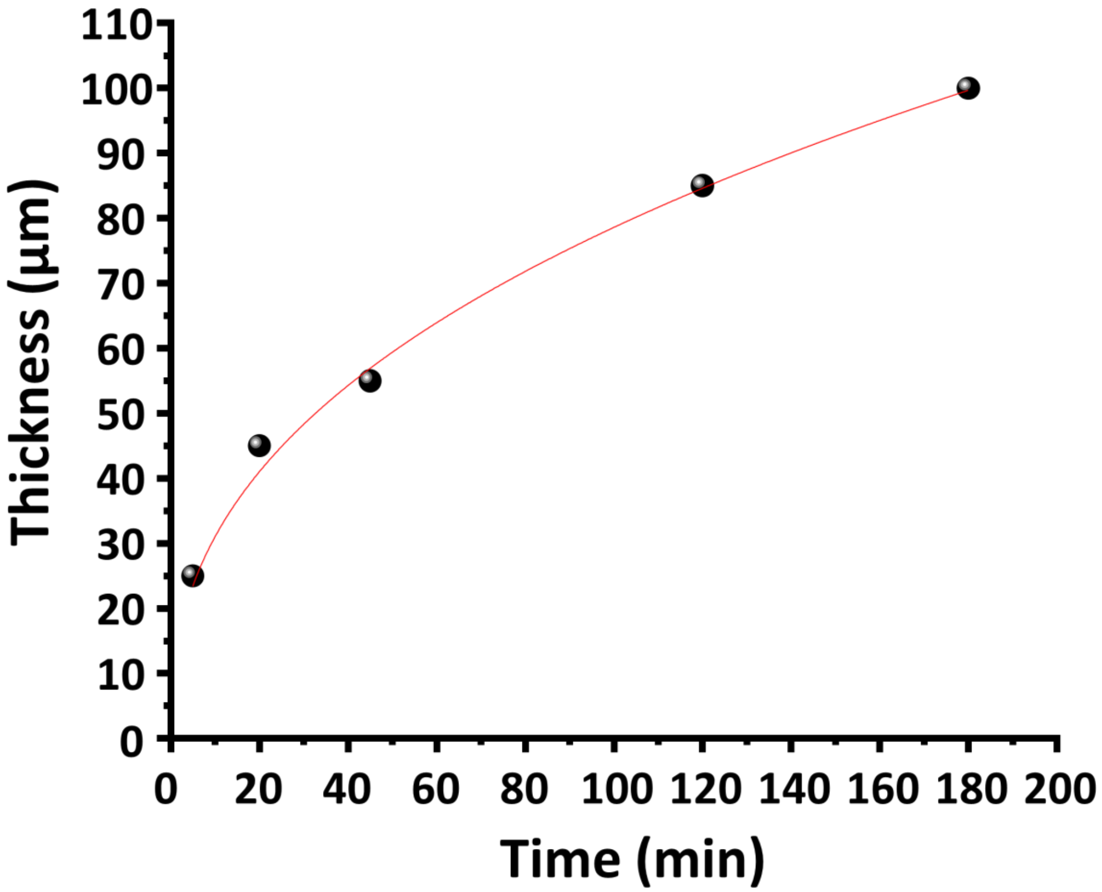

Figure S14. Time-dependent thickness study of TTA-DFP-COF-**5/25/45/120/180** thickness was measured by SEM.

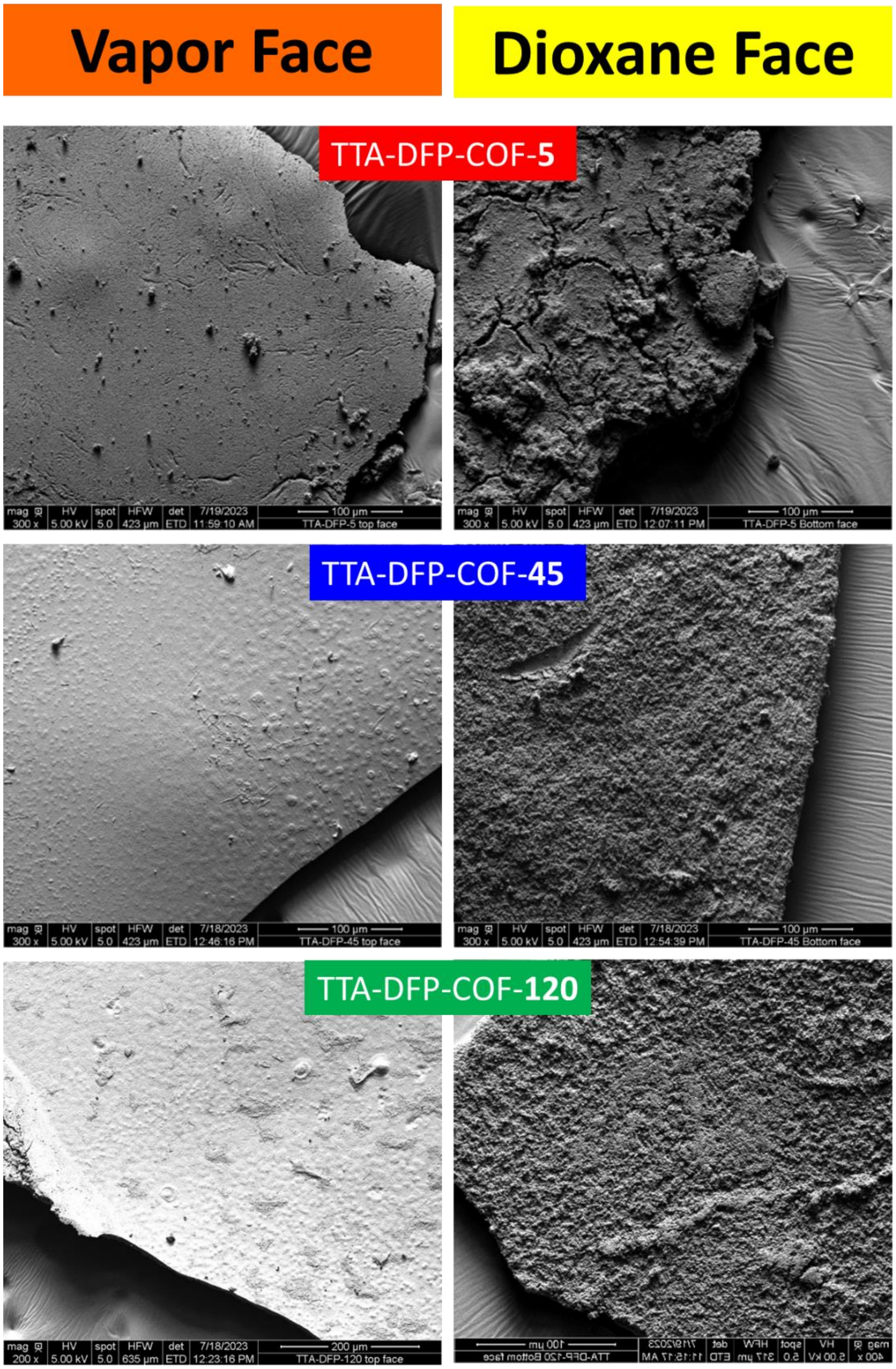

Figure S15. SEM images of vapor (left panel) and dioxane (right panel) faces of TTA-DFP-COF-**5**, TTA-DFP-COF-**45,** and TTA-DFP-COF-**120**.

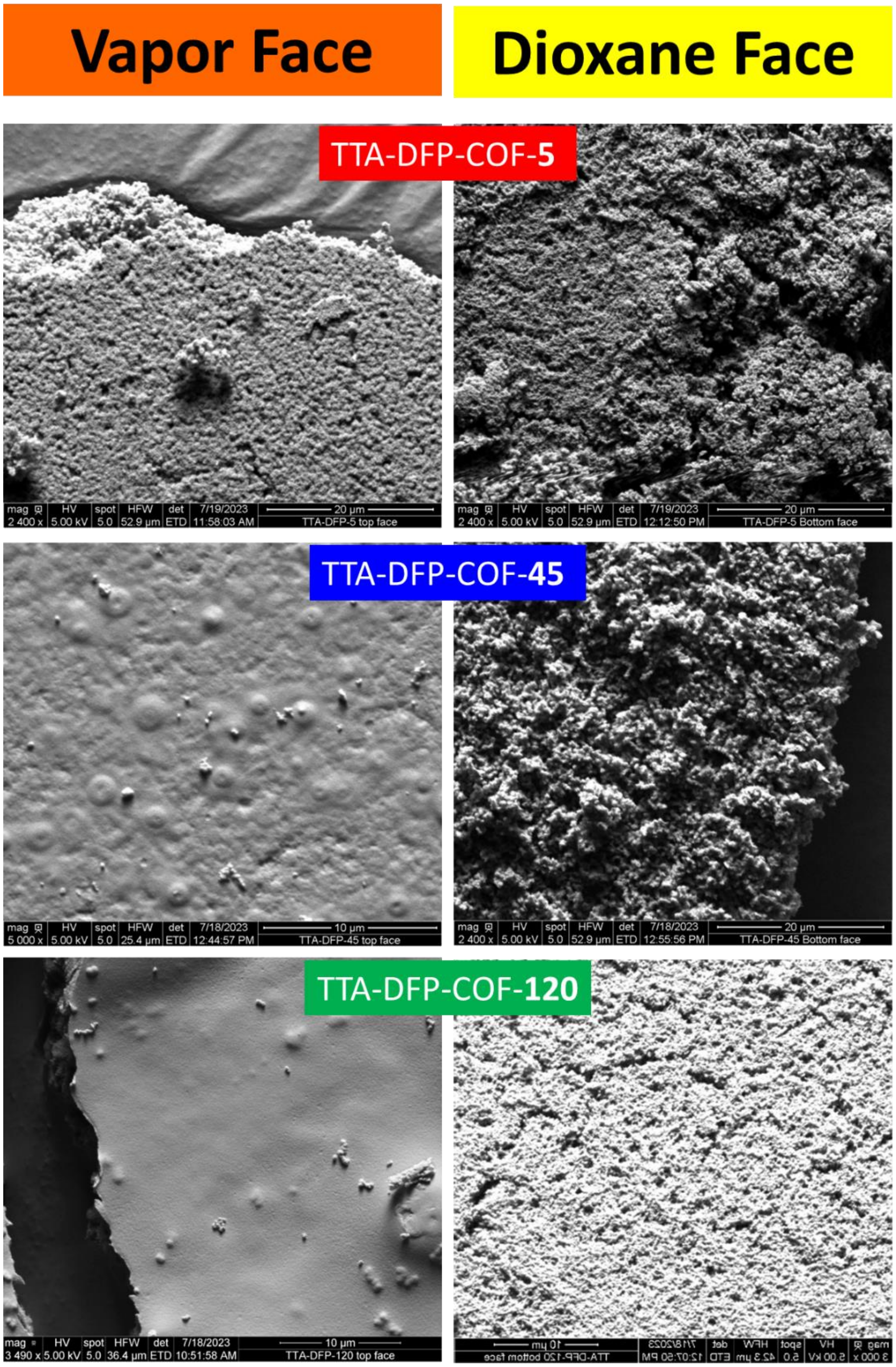

Figure S16. SEM images of vapor (left panel) and dioxane (right panel) faces of TTA-DFP-COF-**5**, TTA-DFP-COF-**45,** and TTA-DFP-COF-**120** at higher magnifications.

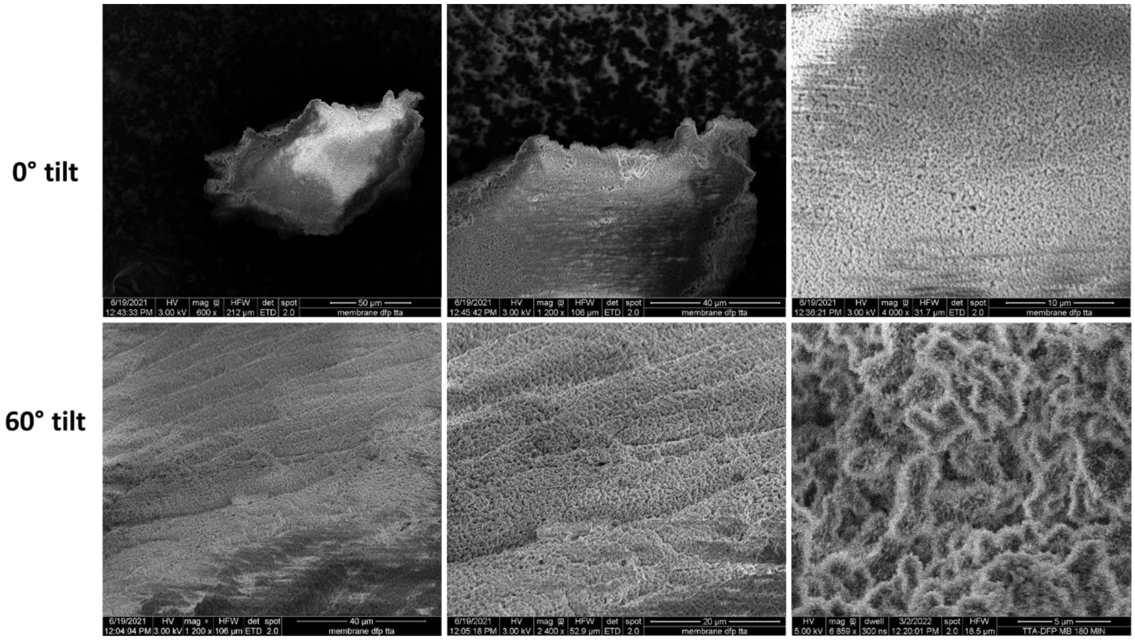

Figure S17. SEM images of TTA-DFP-COF-**120** with a tilt angle of 0 and 60°.

3.5. Atomic force microscopy (AFM)

The TTA-DFP-COF membranes could be readily exfoliated into COF nanosheets through ultrasound treatment, allowing in-depth examination of the continuous structure using AFM. The nanosheets, which are about 6 nm thick, are formed by the systematic stacking of COF nanoparticles, serving as the foundation for the membrane.

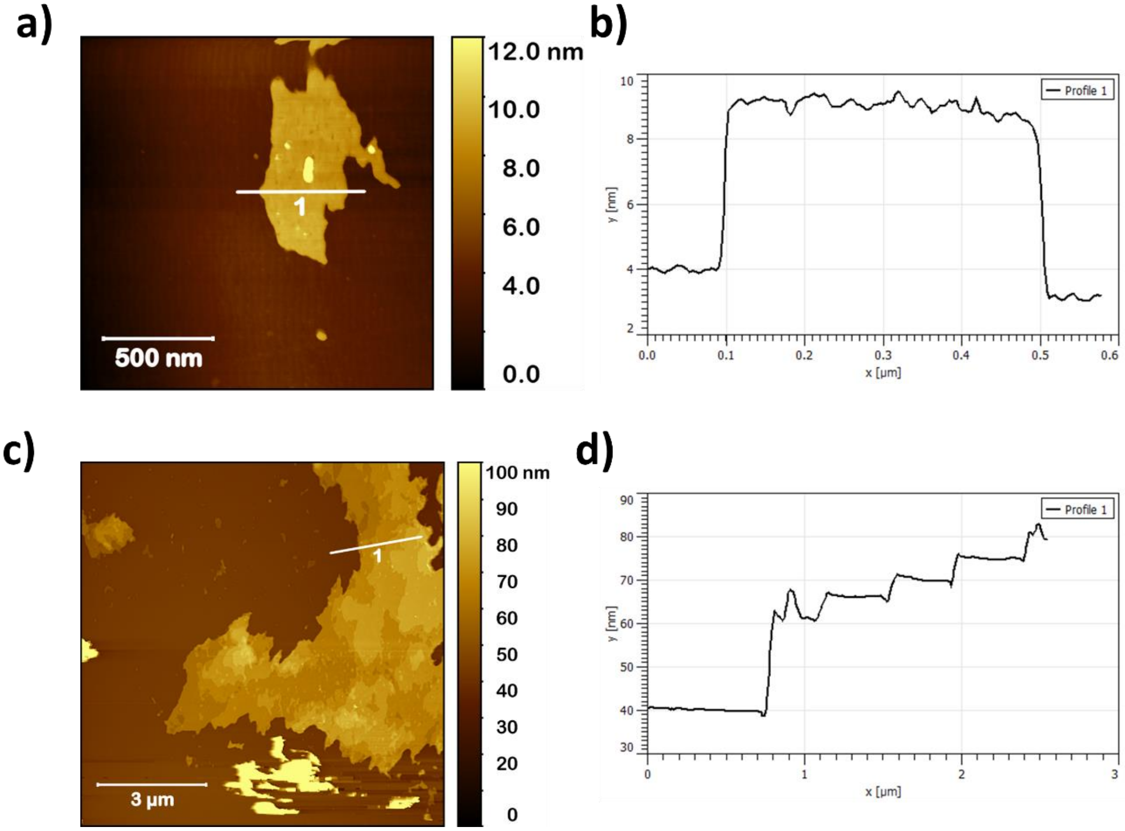

Figure S18. AFM images (a, c) and height profiles (b, d) of exfoliated TTA-DFP-COF-**120**.

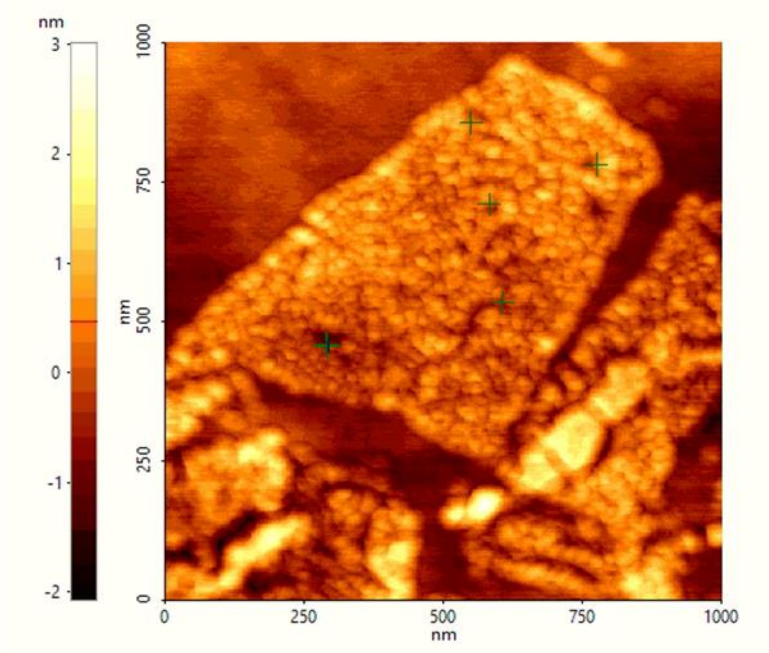

Figure S19. AFM images of exfoliated TTA-DFP-COF-**120**.

3.6. Powder X-ray diffraction (PXRD) measurements

Powder X-ray diffraction (PXRD) measurements were carried out to confirm the crystalline nature of the framework. The TTA-DFP-COF membranes were found to be highly crystalline in nature. In fact, we observed a strong peak at 2θ of 4.8 ° assigned to the (110) plane of the regularly ordered lattice. TTA-DFP-COF membrane shows a broad peak at ~25.5, corresponding to the reflection from the (003) plane.

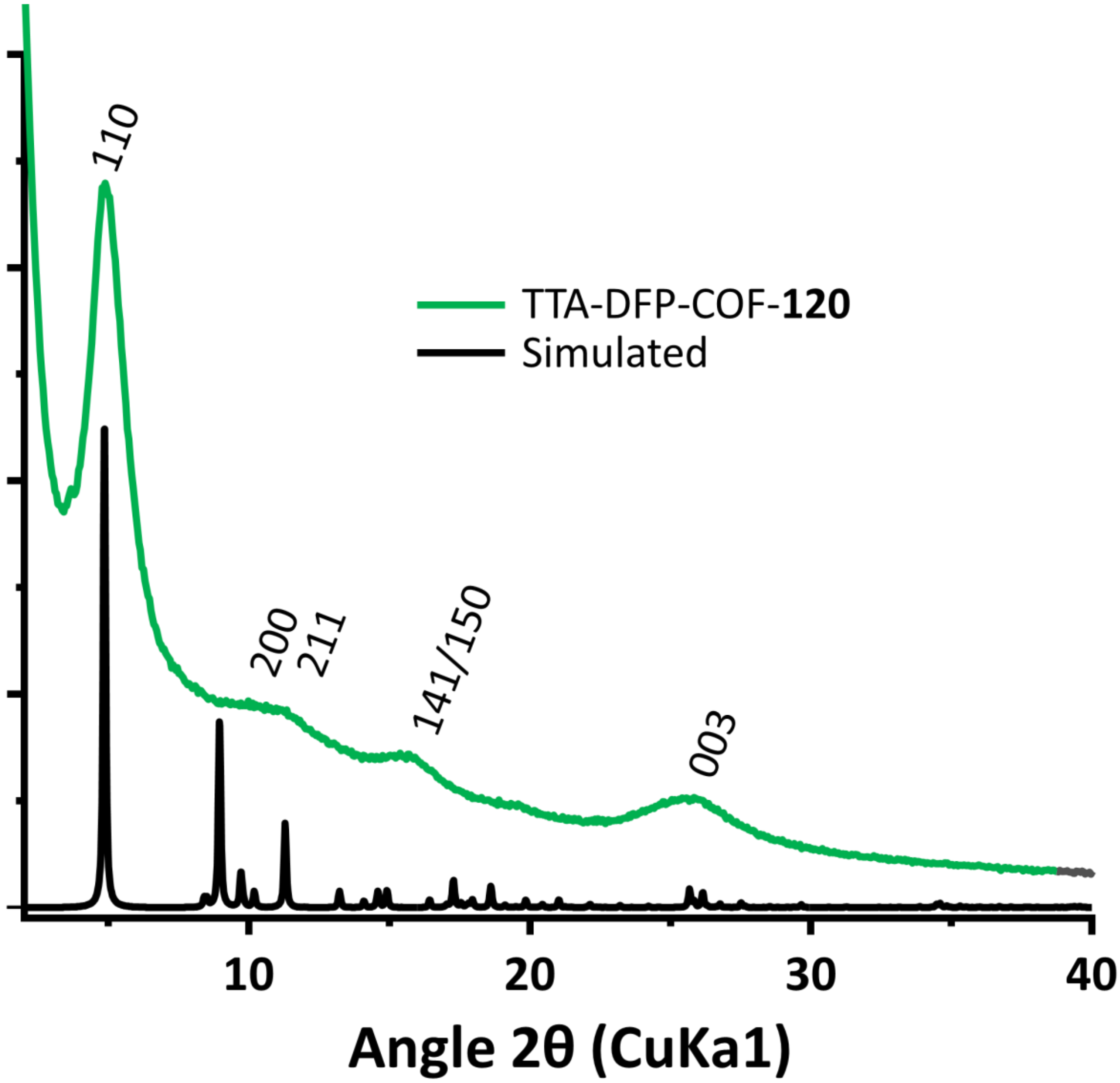

Figure S20. PXRD analysis of TTA-DFP-COF**-120** membrane. Comparison of experimental PXRD pattern and calculated from the proposed crystal structure.

3.7. High-resolution transmission electron microscopy (HRTEM)

High-resolution transmission electron microscopy (HRTEM) images were obtained using a Talos F200X Scanning/Transmission Electron Microscope with a lattice-fringe resolution of 0.14 nm at an accelerating voltage of 200 kV equipped with a CETA 16M camera. The samples were prepared on holey carbon film mounted on a copper grid. A drop of diluted particle solution was spotted on the grid and dried overnight at room temperature (298 K). All the relevant areas were marked using bright field imaging mode at spot size 3, and the marked areas were also scanned using the STEM-HDAAF mode at spot size 9 for imaging and spot size 6 for conducting the STEM-EDAX. The STEM mode helps in providing the elemental composition as it works on the principle of mass determination. Such measurements can be performed at low electron dose by collecting the high-angle dark-field signal using an annular detector. This mode is generally used to image the elements with different masses, with the heavier mass element appearing brighter. The samples were scanned at spot size 9 and with a screen current of 60 pA. The data was analyzed using Velox analytical software.

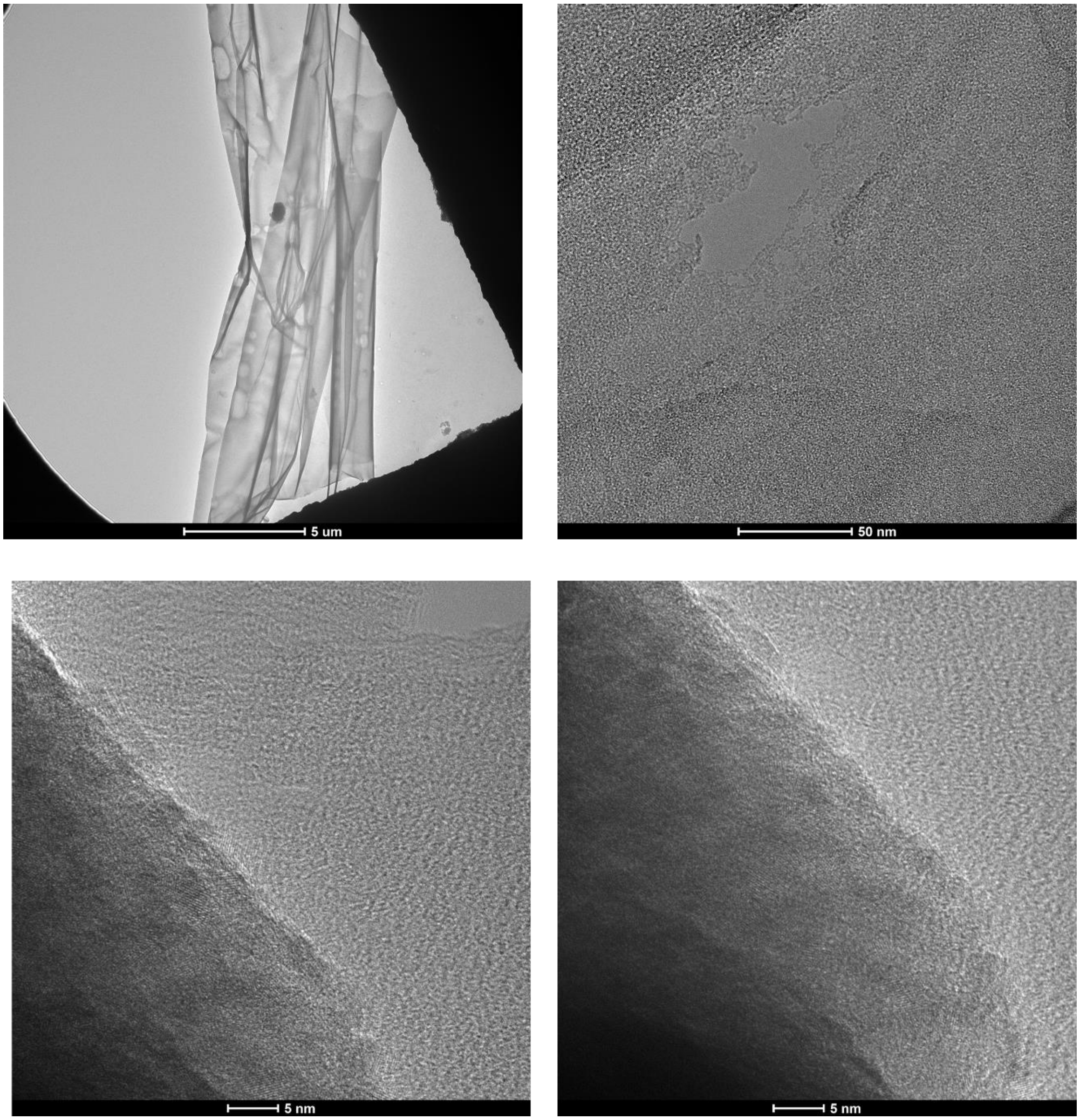

Figure S21. HRTEM images of exfoliated TTA-DFP-COF-**120**.

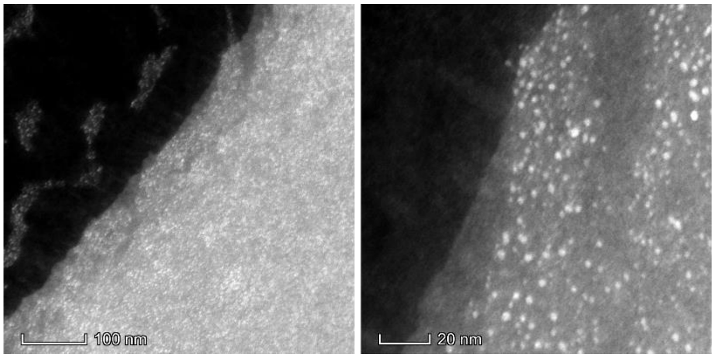

Figure S22. STEM image of exfoliated TTA-DFP-COF-**120**.

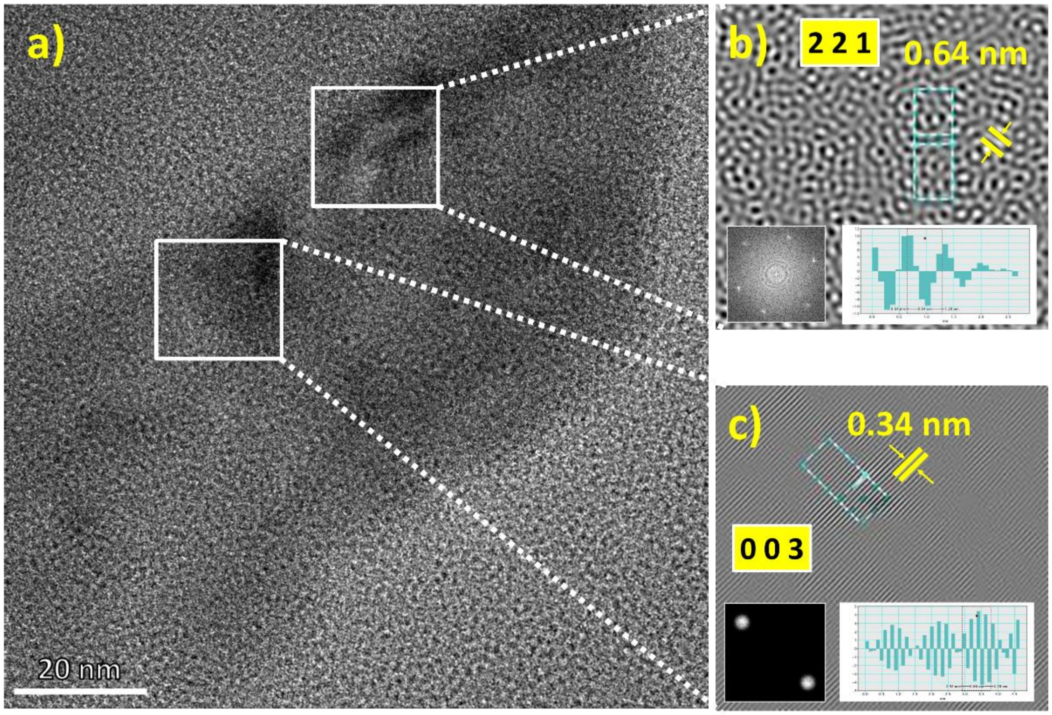

Figure S23. HRTEM analysis of TTA-DFP-COF-**120** confirming the material's crystallinity: a) HR-TEM image displaying lattice fringes and b) and c) images of lattice fringe reconstruction. Lattice fringe distances (d = 0.34 and 0.64 nm) corresponding to the (003) and (221) planes of the COF

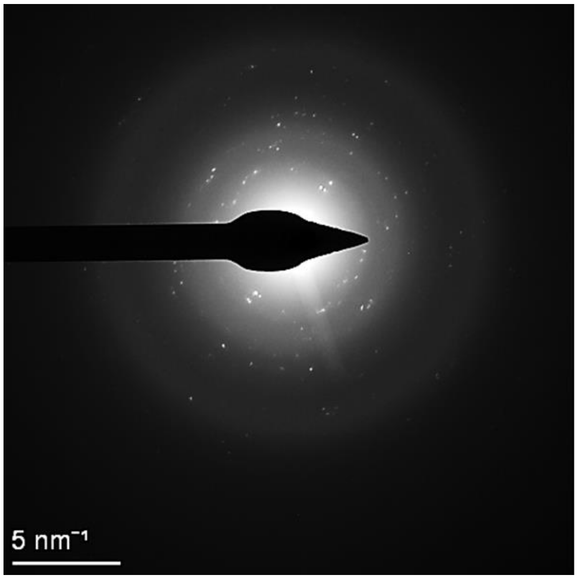

Figure S24. Selective area electron diffraction (SAED) image indicate the TTA-DFP-COF membrane's crystallinity.

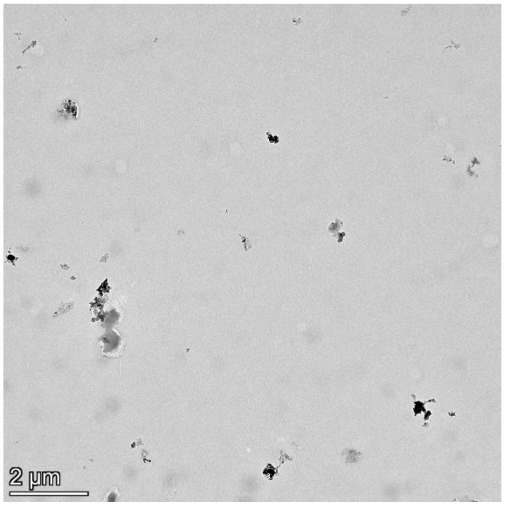

Figure S25. TEM image of the reaction in the absence of acetic acid, only linkers.

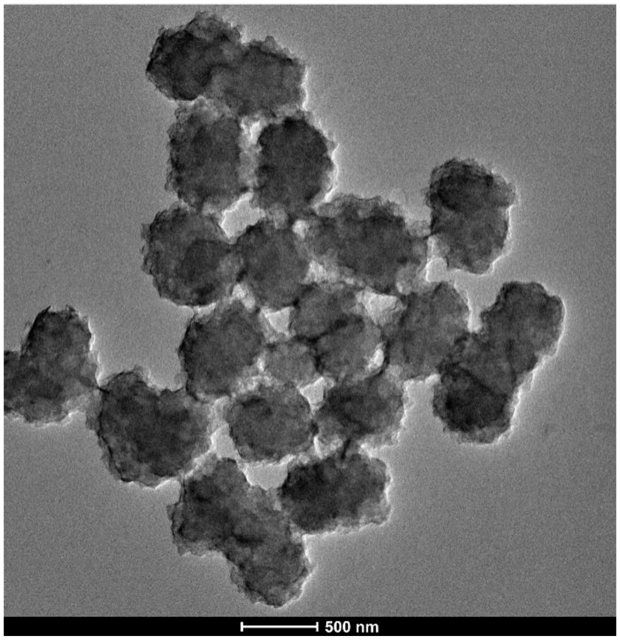

Figure S26. TEM image of the liquid phase (dioxane) after membrane synthesis.

3.8. N_2 adsorption-desorption experiments

Low-pressure gas adsorption measurements were performed on 3-Flex Surface Characterization Analyzer (Micromeritics) at relative pressures up to 1 atm. The cryogenic temperatures were controlled using liquid nitrogen baths at 77 K. The apparent surface area was determined from N_2 adsorption isotherm collected at 77 K by applying the Brunauer-Emmet-Teller (BET) model between P/P_0 values of 0.0005 and 0.05 for microporous COFs.

In a typical experiment, 20 to 30 mg of homogenous polycrystalline samples of TTA-DFP-COF membranes are transferred (dry) to a 6-mm large bulb glass sample cell and are evacuated at room temperature using a turbo molecular vacuum pump and then gradually heated to 125 °C, held for 24 h and cooled to room temperature.

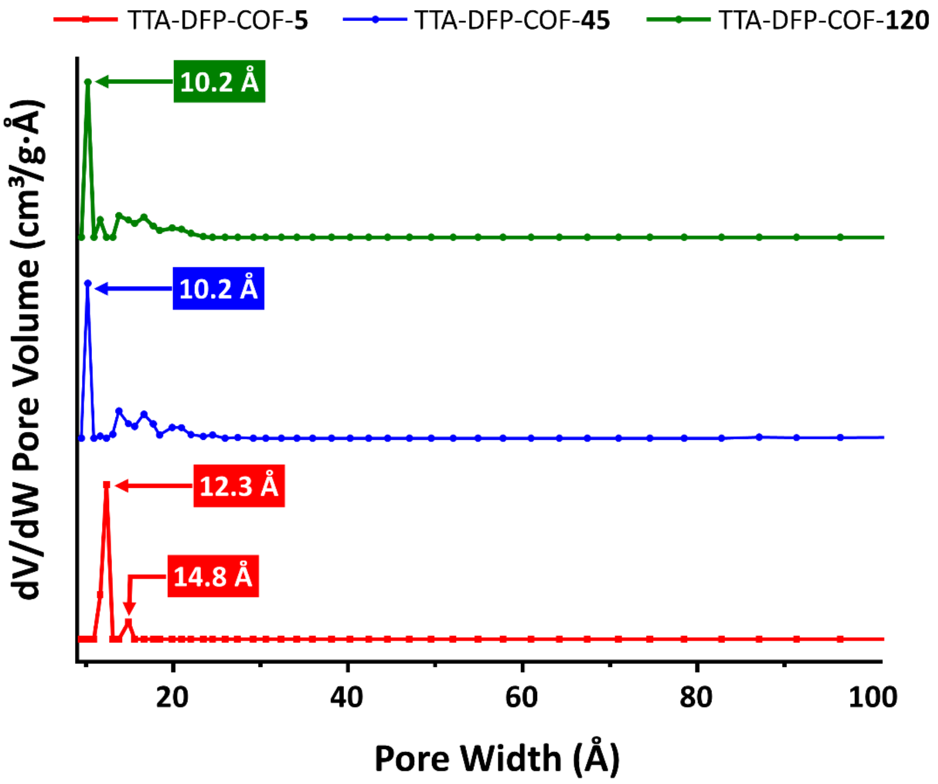

Figure S27. Pore size distribution of TTA-DFP-COF-**5** (red), TTA-DFP-COF-**45** (blue), and TTA-DFP-COF-**120** (green).

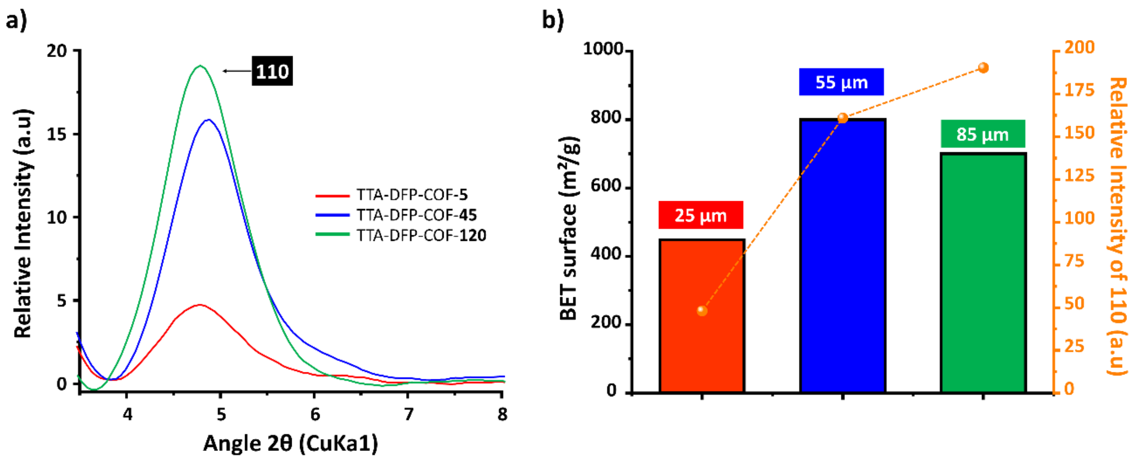

Figure S28. a) PXRD of TTA-DFP-COF-**5** (red), TTA-DFP-COF-**45** (blue), and TTA-DFP-COF-**120** (green) between 3 and 8° corresponding to the 110 peak. b) BET surface versus intensity of the 110 peak of TTA-DFP-COF-**5** (25 µm, red), TTA-DFP-COF-**45** (55 µm, blue), and TTA-DFP-COF-**120** (85 µm, green).

3.9. Mechanical properties by nano-indentation

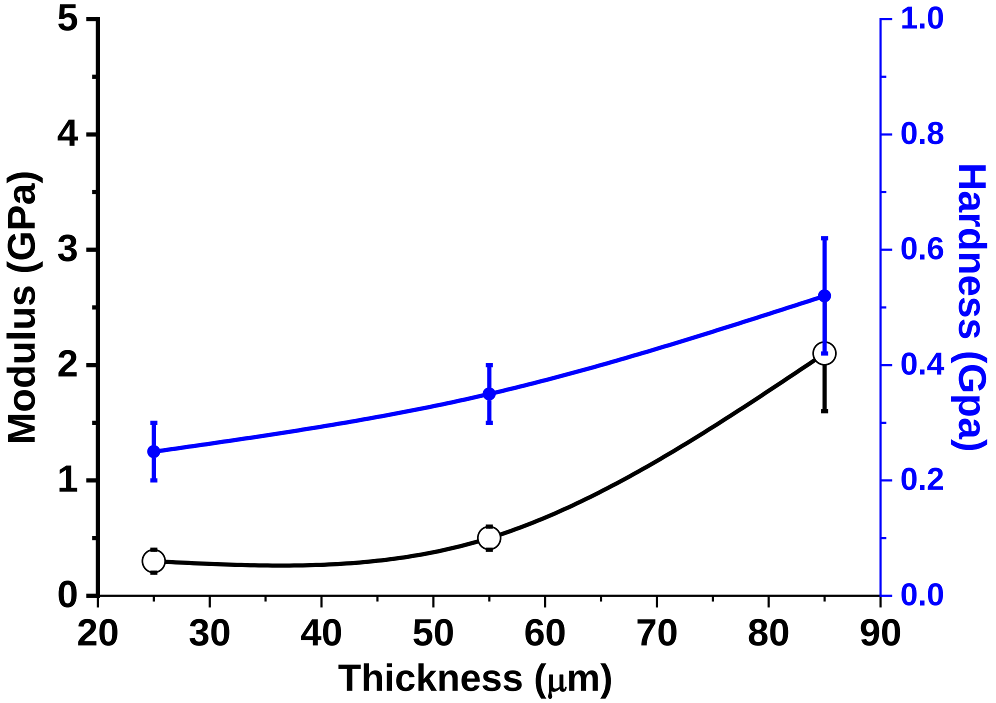

Figure S29. Mechanical properties as a function of the thicknesses of TTA-DFP-COF-**5/45/120**, Young modulus (black), and hardness (blue) were deduced from loading displacement curves.

Table S2. Comparison of the mechanical properties obtained by nanoindentation (Young's modulus (*Er*) and hardness (*H*)) of TTA-DFP-COF-**5/45/120** membrane vs. reported membranes.

Membrane	Young's modulus (Er) and hardness (H)	Reference
**amide-COF**	*Er* = 104.5 MPa, *H* = 21.5 MPa	Lu *et Al.*[11]
**COF-1**	*Er* = 3064 MPa, *H* = 230 MPa	Turangan *et Al.*[12]
**Tp–Azosphere**	*Er* ≈ 15300 MPa, *H* ≈ 66 MPa	Dey *et Al.*[13]
**COF-DhTGCl**	*Er* ≈ 350 MPa, *H* ≈ 278 MPa	Wang *et Al.*[14]
**FCOF**	*Er* ≈ 30000 MPa, *H* ≈ 1200 MPa	Zhao *et Al.*[15]
**poly3/6COF-42**	*Er* = 914 MPa	Wang *et Al.*[16]
**TAPB-BTCA-MCOF**	*Er* =800 MPa	Martín-Illán *et Al.*[17]
**TAPB-PDA COF**	*Er* = 1400 MPa	Zhu *et Al.*[18]
**COF-5**	*Er* = 59.42 MPa	Duan *et Al.*[19]
**LZU-8**	*Er* = 22.78 MPa	Wang *et Al.*[20]
**TpPa-SO3H**	*Er* = 2557 MPa	Hou *et Al.*[21]
**TFG-EDA**	*Er* = 144 MPa	Mishra *et Al.*[22]
**HKUST-1**	*Er* = 1010 MPa	Bundschuh *et Al.*[23]
**MIL-53 membrane**	*Er* = 7010 MPa, *H* = 82.4 MPa	Zhang *et Al.*[24]
**EM400/MIL-101(Cr)–NH_2 MMMs**	*Er* = 650 MPa	Chen *et Al.*[25]
**Poly(alkyl cyanoacrylate)s (PACAs)**	*Er* = 4000-17000 MPa	Altabal *et Al.*[26]
**PAE film**	*Er* = 3900 MPa, *H* = 320 MPa	Xu *et Al.*[27]
**Freestanding Poly (Methyl Methacrylate)/Monolayer Graphene Membrane**	*Er* = 180 MPa	Bais *et Al.*[28]
TTA-DFP-COF-**5**	*Er* = 300 MPa, *H* = 250 MPa	This work
TTA-DFP-COF-**45**	*Er* = 500 MPa, *H* = 350 MPa	
TTA-DFP-COF-**120**	*Er* = 2100 MPa, *H* = 520 MPa	

3.10. Water contact angle

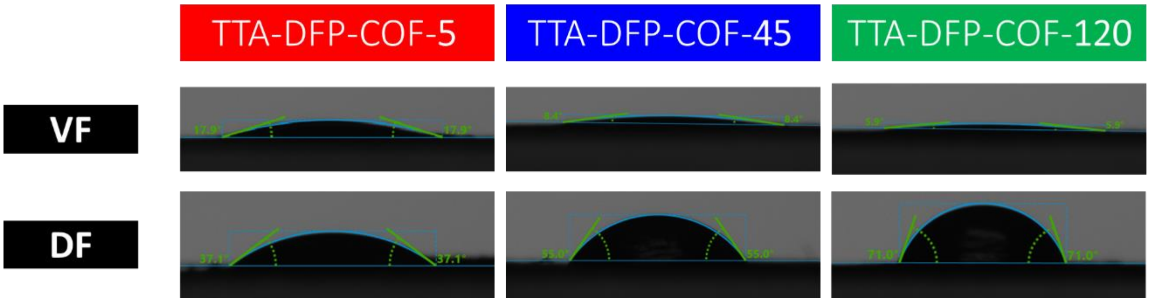

Figure S30. Digital images of water contact angle (WCA) of the vapor face (VF, top) and dioxane face (DF, bottom) of TTA-DFP-COF-**5**, TTA-DFP-COF-**45,** and TTA-DFP-COF-**120**.

3.11. Raman spectroscopy

The two faces of the TTA-DFP-COF membrane were characterized using Raman microscope (Horiba Labram HR Evolution). A green laser at 532 nm wavelength was used for excitation with a 50x objective. The focal length was optimized to minimize the laser's penetration depth.

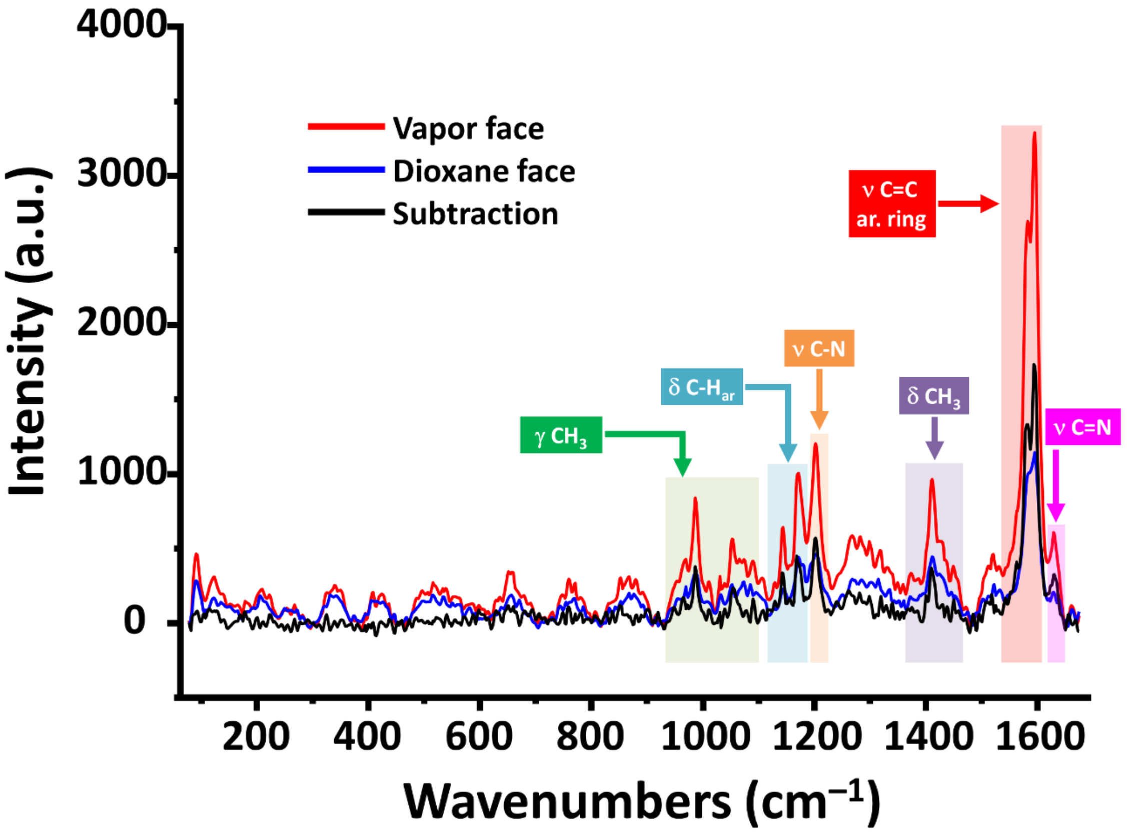

Figure S31. Raman spectra of vapor face (hydrophilic, red), dioxane face (near-hydrophobic, blue) of the TTA-DFP-COF membrane and their corresponding subtraction (black).

3.12. ATR-FTIR spectroscopy

A Nicolet Nexus FTIR Spectrometer (Nicolet Instrument Co., Madison, USA) equipped with a DTGS (Deuterated Triglycine Sulphate) detector was employed. A diamond ATR accessory (DuraSample IR–Technologies Danbury, USA) was used for ATR FTIR experiments. Each spectrum comprises 128 scans measured at a spectral resolution of 4 cm^{-1} in the 4000–600 cm^{-1} range.

Table S3. Experimental ATR FTIR bands (at wavenumbers/cm^{-1}) of the TTA-DFP-COF membrane and vibrational assignment of the infrared bands.

Band position (cm^{-1})	Assignment
3070	C-H stretch. (arom.)
2977	C-H antisym. stretch. (CH_3 group)
2925	C-H(stretch.)
2868	C-H sym. stretch. (CH_3 group)
1710<br>1680	C=O (aldehyde)<br>C=O (N-phenyl acetamide)
1607-1580	C-C & C=C stretch. ring
1511	C=N stretch. + C-H bend.
1480	NH bend. (amide)
1453	C-H bend. ring + C-C stretch.
1409	C-H bend. + N-H bend
1350	C–N stretch. or CH(methyl) bend and combination
1325	C-C stretch and C-N-C stretch. and C-H(arom.) bend. and combination
1176	C-H bend.
1145	C-H bend. + C-C stretch.
866-816	ring def.

3.13. X-ray photoelectron spectroscopy (XPS)

X-ray photoelectron spectroscopy (XPS) analysis was performed to analyze the elemental composition and understand the interactions in the membrane. XPS experiments were carried out on a Kratos Axis Ultra DLD spectrometer under a base pressure of ∼ 2×10^{-10} mbar. A monochromated Al Kα X-ray source (1486.69 eV) irradiated samples at room temperature. XPS spectra were recorded from an analysis area of 700 µm × 300 µm. High-resolution XPS data of core levels were obtained with an energy resolution of 0.05 eV. For consistency, XPS measurements were calibrated to C1s (∼ 285 eV). Data were analyzed using CasaXPS package with Shirley background subtraction.

Our approach employs the pseudo-Voigt function, chosen for its robustness and effectiveness in integrating a weighted blend of Gaussian (70%) and Lorentzian (30%) profiles. We have meticulously adjusted the deconvolution process to accurately accommodate multiple subsidiary peaks, ensuring a precise and reliable fit. This adjustment aims to achieve an expected χ^2 value around 1, adhering closely to the established physical principles underlying X-ray Photoelectron Spectroscopy (XPS). A key aspect of our method involves ensuring physical plausibility, such as maintaining consistent Full Width at Half Maximum (FWHM) values for comparable components to accurately represent core-hole broadening effects.

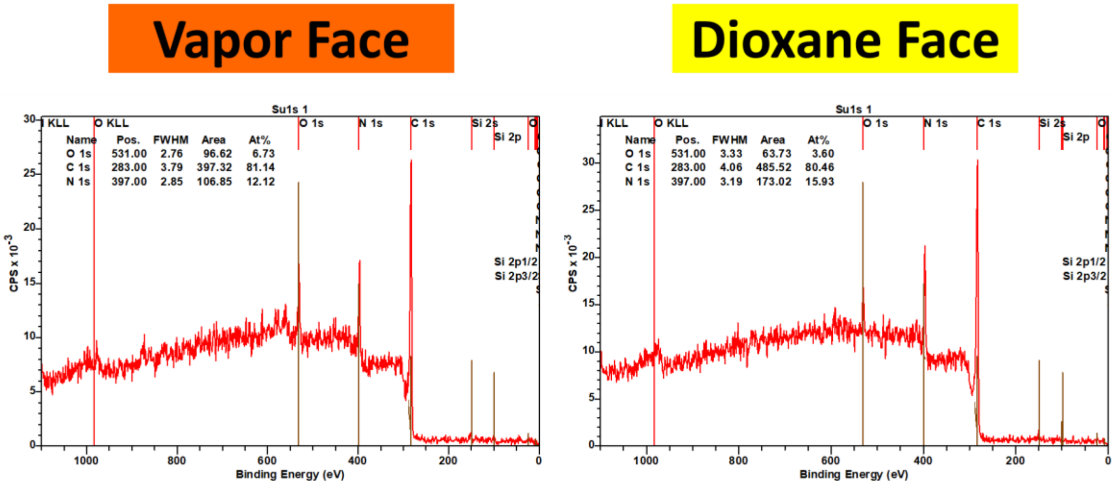

Figure S32. XPS survey spectrum of vapor and dioxane faces of the TTA-DFP-COF membrane.

Table S4. Peak % for C, N, and O of vapor and dioxane faces of the TTA-DFP-COF membrane

Element	Vapor Face	Dioxane Face
**C 1s**	81.1 %	80.5 %
**N 1s**	12.1 %	15.9 %
**O 1s**	6.7 %	3.6 %

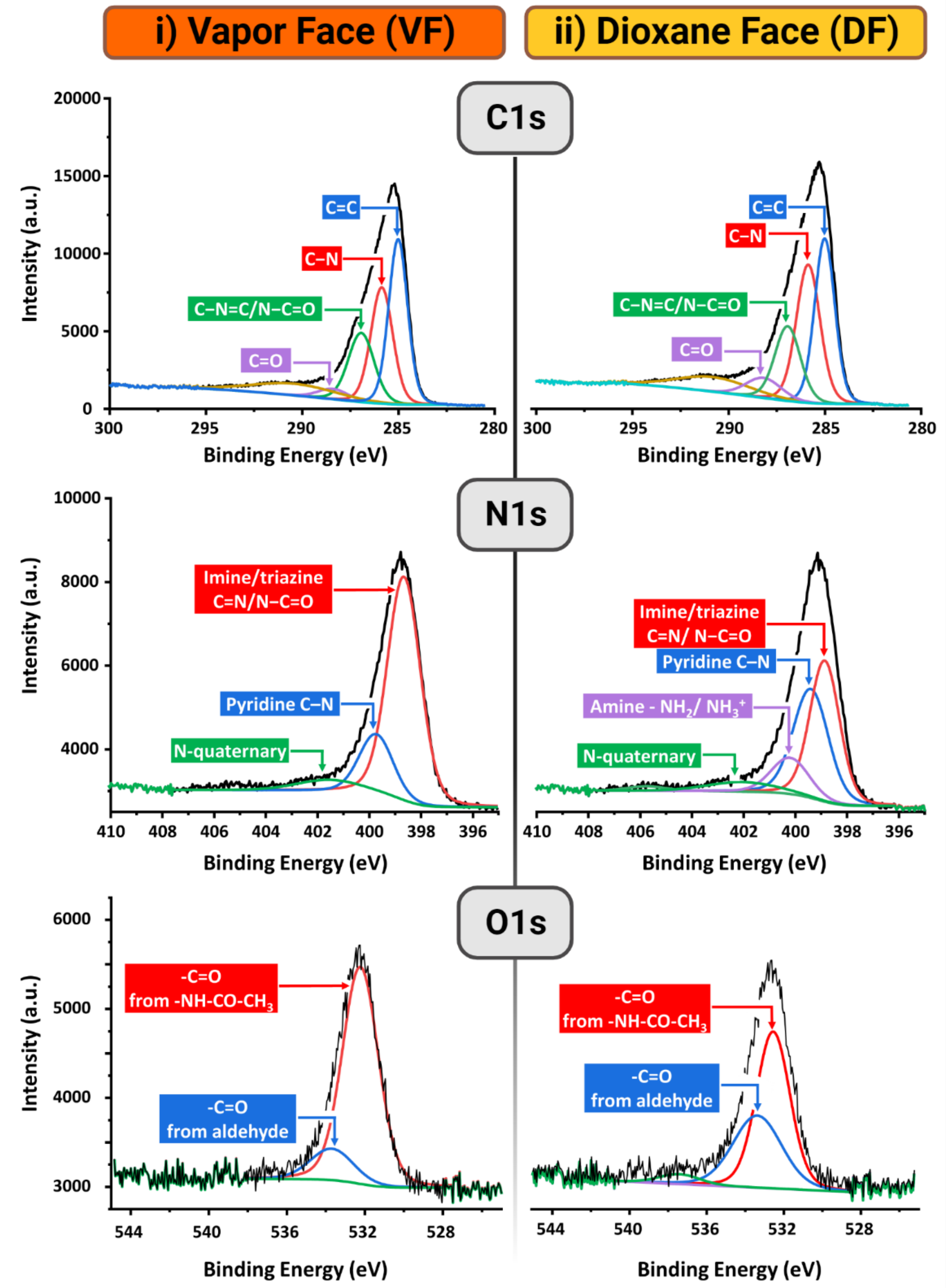

Figure S33. XPS a) C 1s, b) N 1s, and c) O 1s spectra of vapor (left panel) and dioxane (right panel) faces of the TTA-DFP-COF membrane.

Table S5. Binding energy (eV) and peak % for C 1s spectrum deconvolution curves of vapor and dioxane faces of the TTA-DFP-COF membrane (instrumental error is ±0.2eV).

C1s	Vapor Face		Dioxane Face	
Bond	Binding energy (eV)	%	Binding energy (eV)	%
C=C	285.0	29.1	284.9	32.0
C-N	285.8	34.6	285.9	33.3
C–N=C/ N–C=O amide	286.9	21.4	286.9	16.4
C=O from aldehyde	288.5	3.6	288.2	5.0
π-π	291.1	11.2	291.2	10.5

Table S6. Binding energy (eV) and peak % for N1s spectrum deconvolution curves of vapor and dioxane faces of the TTA-DFP-COF membrane (instrumental error is ±0.2eV).

N1s	Vapor Face		Dioxane Face	
Bond	Binding energy (eV)	%	Binding energy (eV)	%
Imine/triazine/C=N/ N–C=O	399.4	50	399.1	37.5
Pyridine C–N	400.1	31.4	399.9	23.7
Amine - NH_2	-	-	400.6	22.1
N-quaternary (protonated pyridine)	402.0	15.9	402.5	14.8

Table S7. Binding energy (eV) and peak % for O1s spectrum deconvolution curves of vapor and dioxane faces of the TTA-DFP-COF membrane (instrumental error is ±0.2eV).

O1s	Vapor Face		Dioxane Face	
Bond	Binding energy (eV)	%	Binding energy (eV)	%
-CO (from -NH-CO-CH_3)	532.3	85.214.8	532.6	57.4
-CHO (from DFP)	533.7	14.8	533.4	38.2
Satellite	-	-	537.4	4.4

3.14. Surface zeta potentials (ζ) measurement

Surface zeta potentials (ζ) measurement was conducted using ZetaSizer (ZEN3600, Malvern Panalytical, UK). A drop of tracer (polystyrene) was used as the charged particle and was added to 2 ml DI water at pH ~ 7, since all characterization and testing were conducted around neutral pH. The ζ potential is calculated with the aid of parameters such as dielectric constant, particle velocity, and solution viscosity.

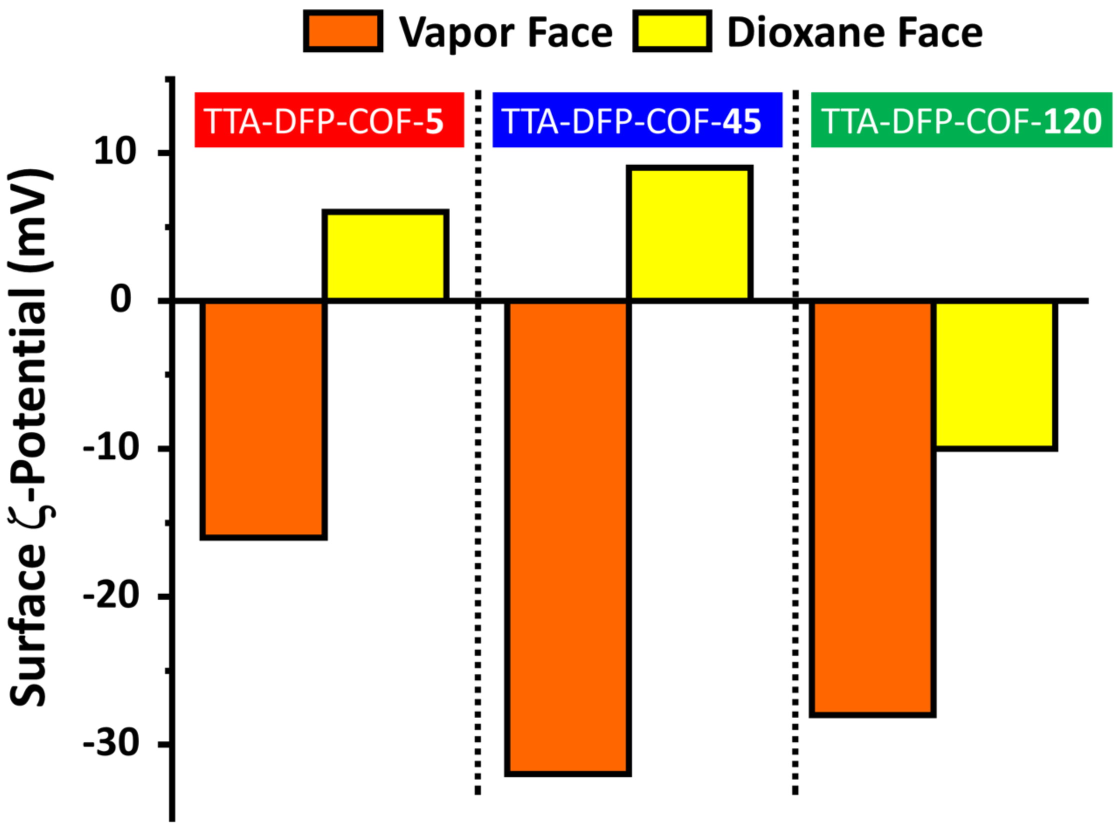

Figure S34. Surface zeta potentials (ζ) measurement for the vapor (orange, superhydrophilic) and dioxane (yellow, near-hydrophobic) faces of TTA-DFP-COF-**5**, TTA-DFP-COF-**45,** and TTA-DFP-COF-**120**.

3.15. Time-dependent TEM/STEM studies of the membrane formation

We executed time-dependent TEM studies by dipping TEM grids at the liquid/vapor interface inside the microwave oven during the synthesis and performed a morphological analysis. The synthesis was slightly altered to slow down the reaction kinetics and simplify the experimental process. We conducted the experiment in an open vessel mode, dipping the grid at various time intervals inside the oven. To prevent water evaporation as the system was no longer sealed, we reduced the reaction temperature to 85 °C. By decreasing the temperature, we achieved a slower reaction rate without affecting its overall outcome.

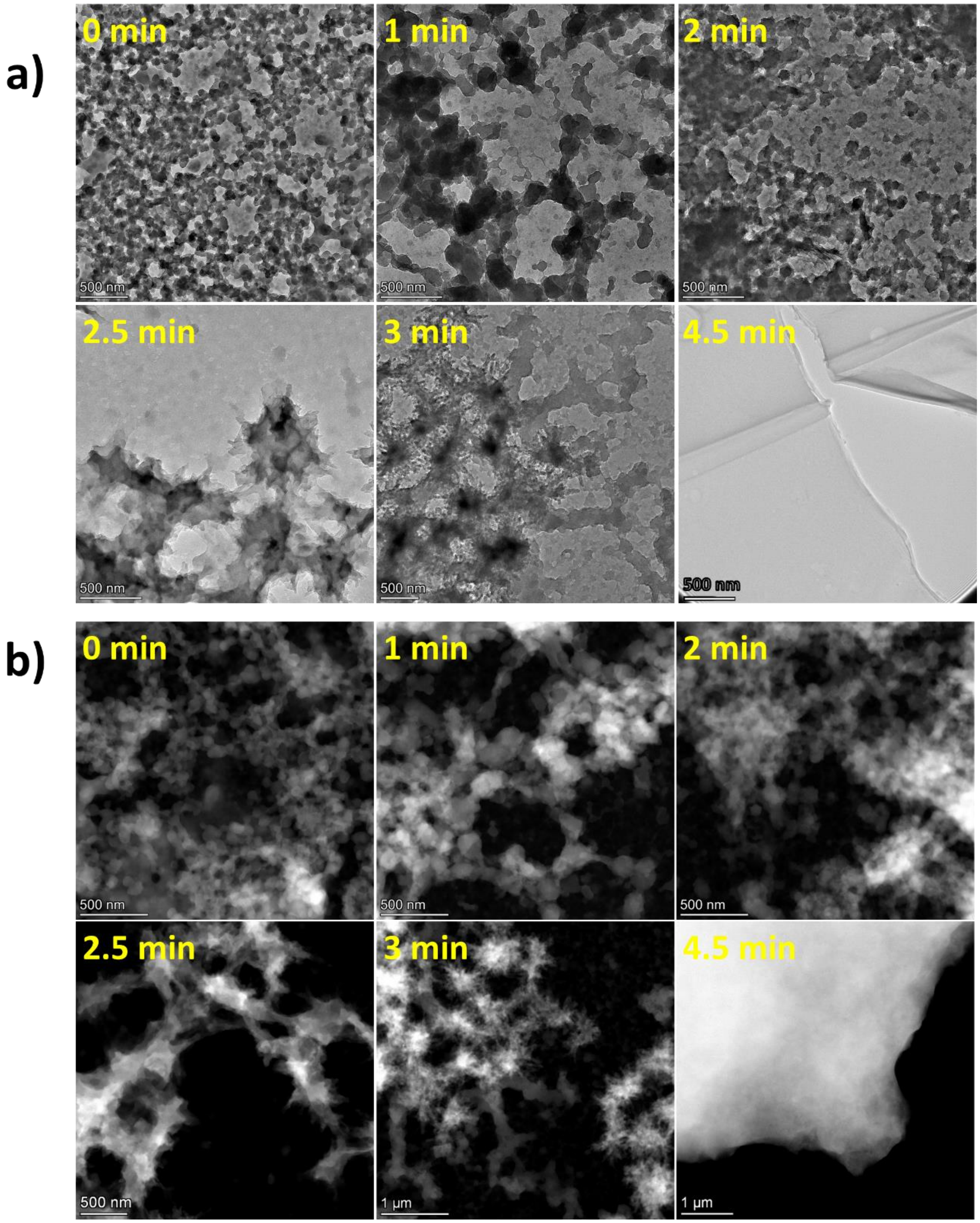

Figure S35. Time-dependent study of the TTA-DFP-COF membrane formation by a) TEM and b) STEM.

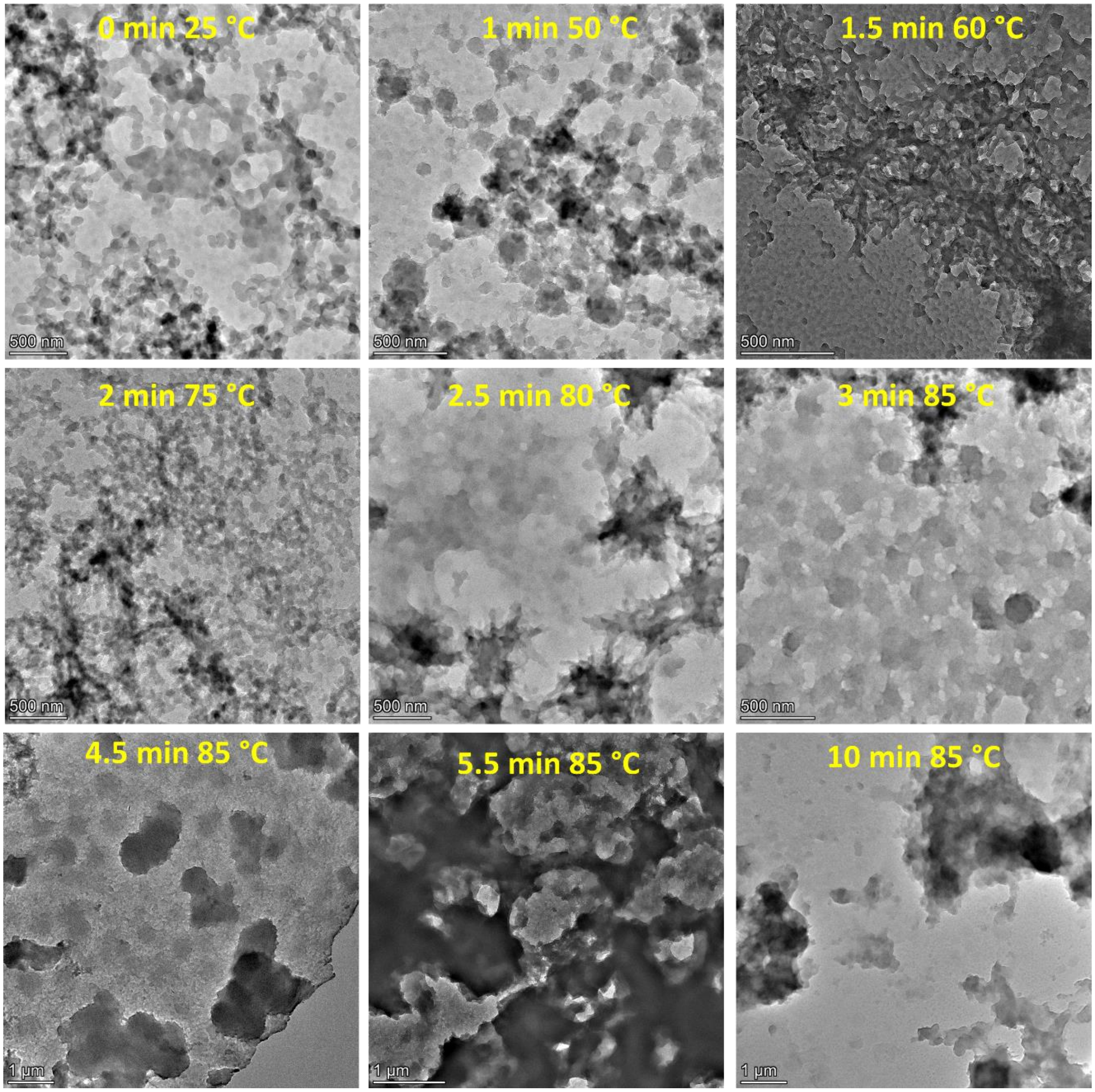

Figure S36. Time-dependent study of the TTA-DFP-COF membrane formation by TEM.

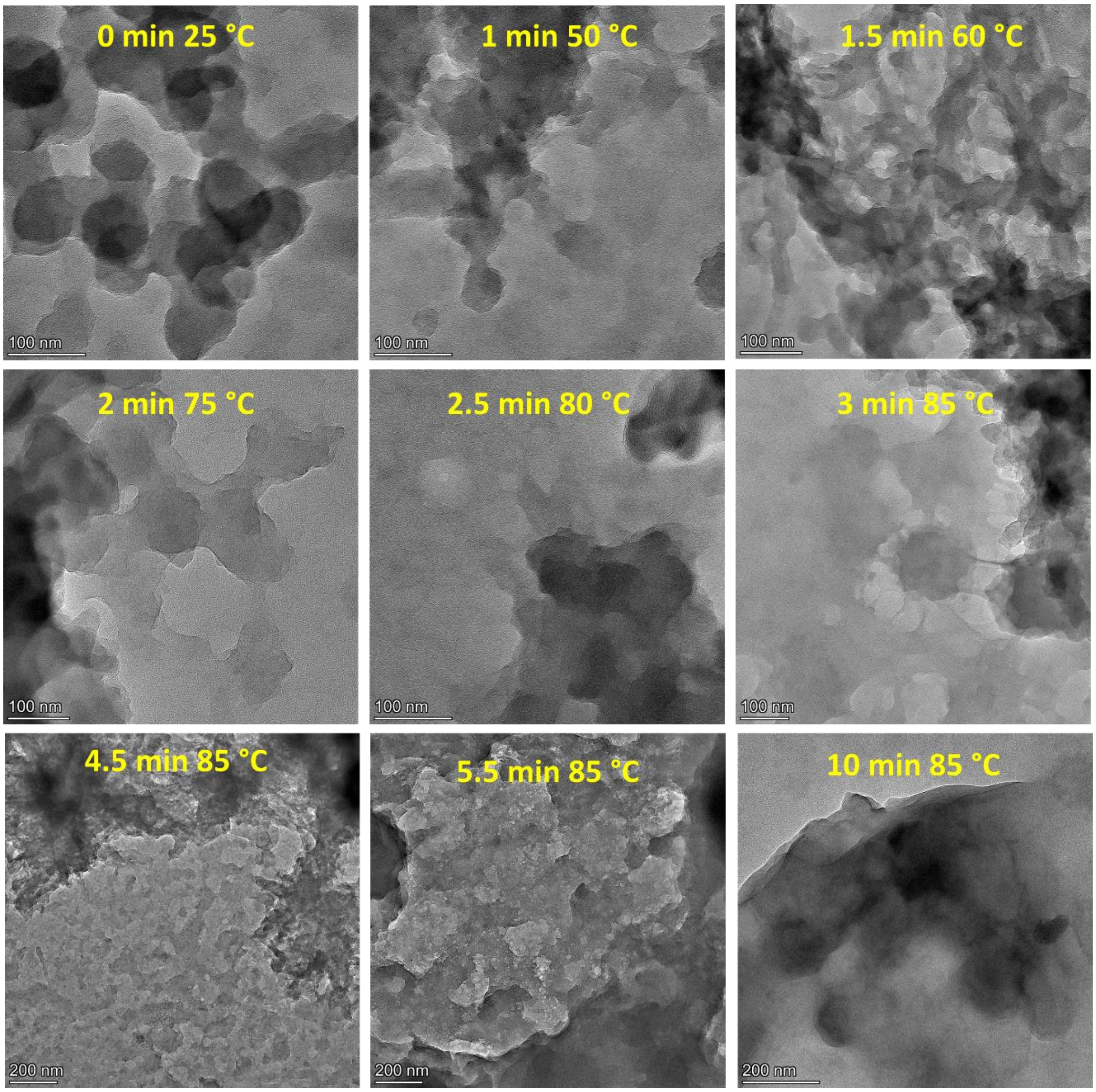

Figure S37. Time-dependent study of the TTA-DFP-COF membrane formation by TEM at higher magnification.

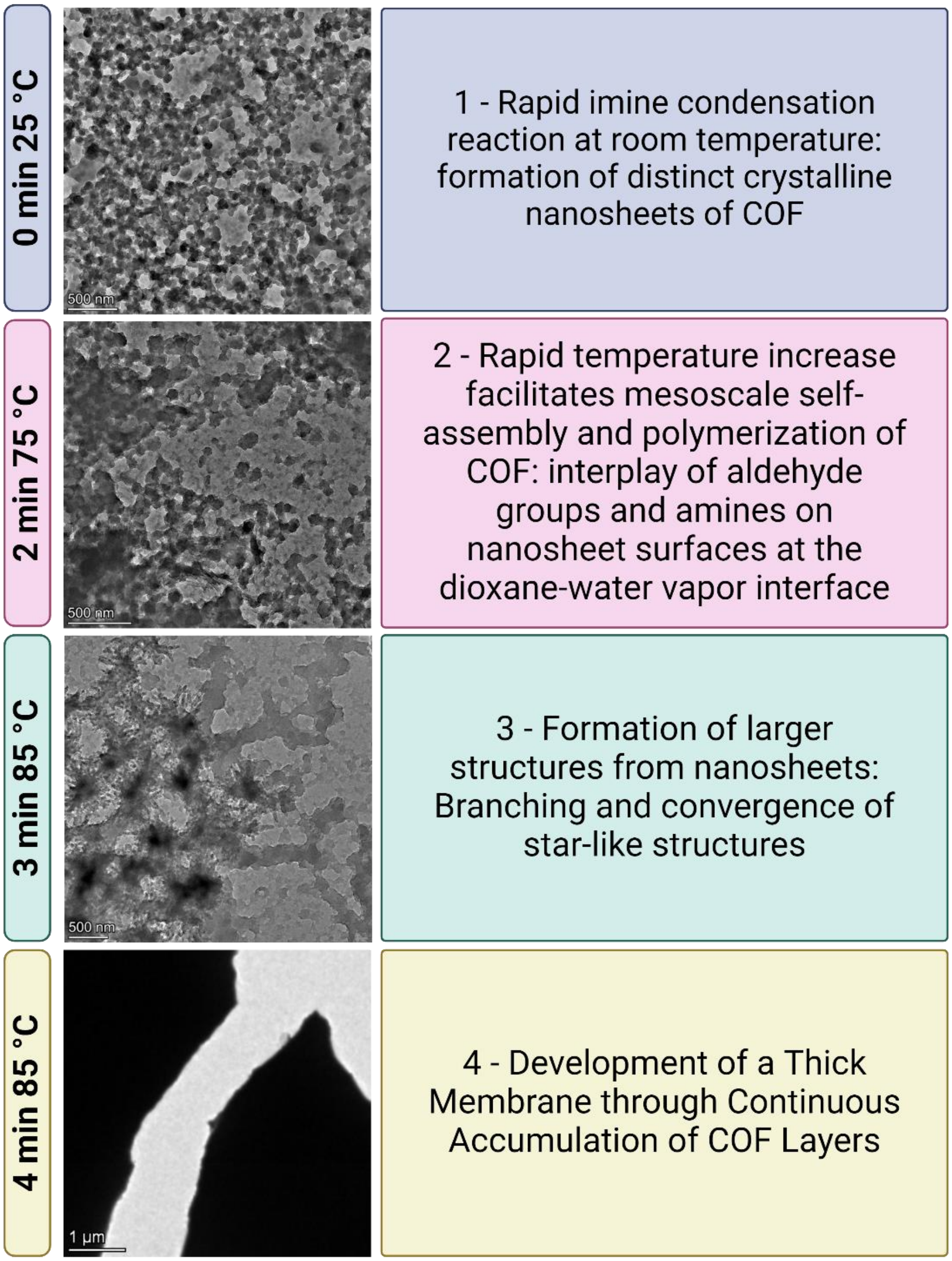

Figure S38. Time-sensitive TEM analysis of TTA-DFP-COF nanosheet synthesis and assembly. **Step 1**: 0 min, room temperature: Rapid imine condensation forms crystalline COF nanosheets in solution. **Step 2**: 2 min, 75°C: Mesoscale self-assembly at the dioxane-water vapor interface. **Step 3**: 3 min, 85°C: Nanosheets form larger structures with branching. **Step 4**: 4 min, 85°C: A thick membrane is formed from accumulated COF layers with a smooth face (VF) and a rough face (DF).

3.16. Thermogravimetric analysis (TGA)

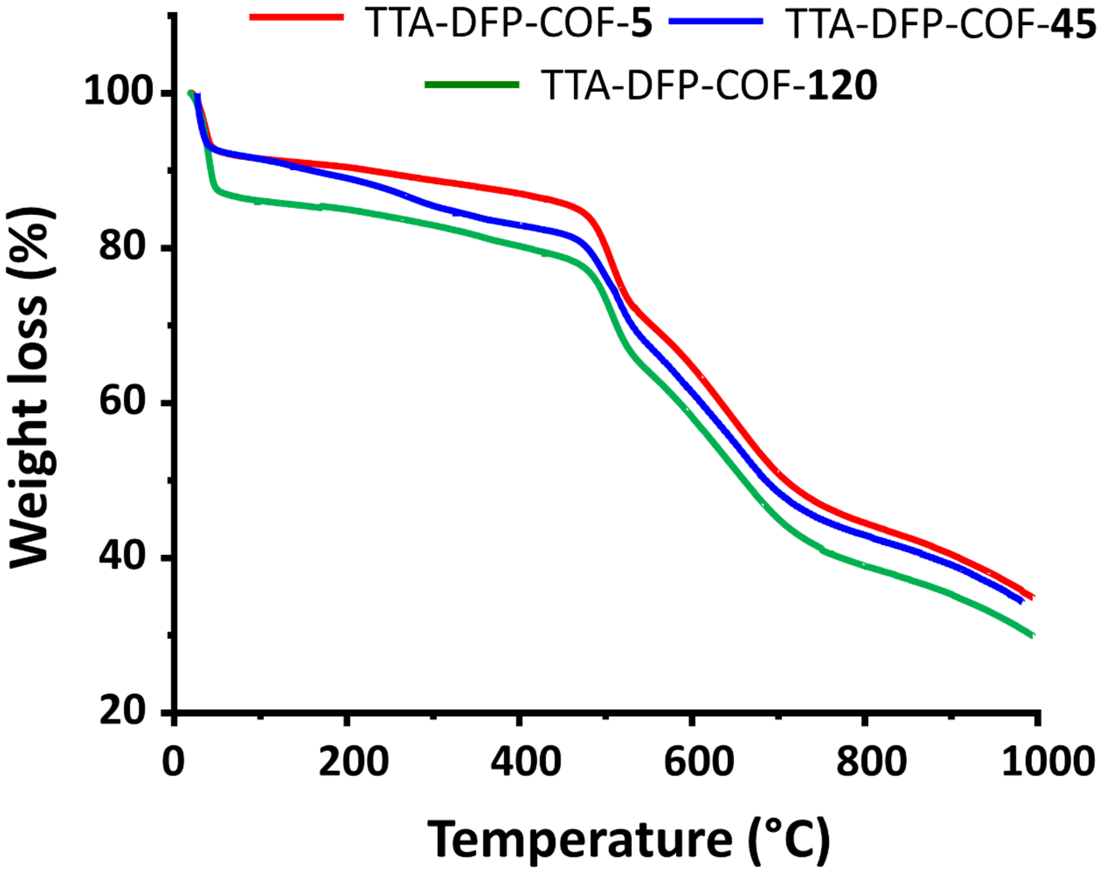

Figure S39. Thermogravimetric analysis (TGA) of TTA-DFP-COF-**5** (red), TTA-DFP-COF-**45** (blue), and TTA-DFP-COF-**120** (green). The thermal stability of the COF profile was recorded at a scan rate of 5 °C/min.

3.17. Stability study

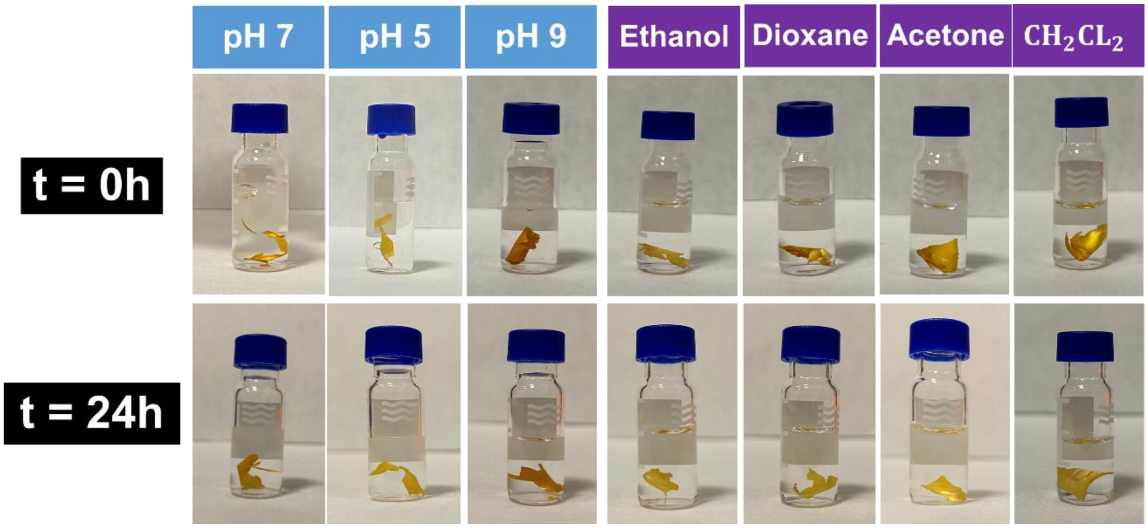

Figure S40. Stability of TTA-DFP-COF-**120** in aqueous solution at pH 5, 7, and 9 and various organic solvents. Membranes were immersed for 24 hours in various conditions.

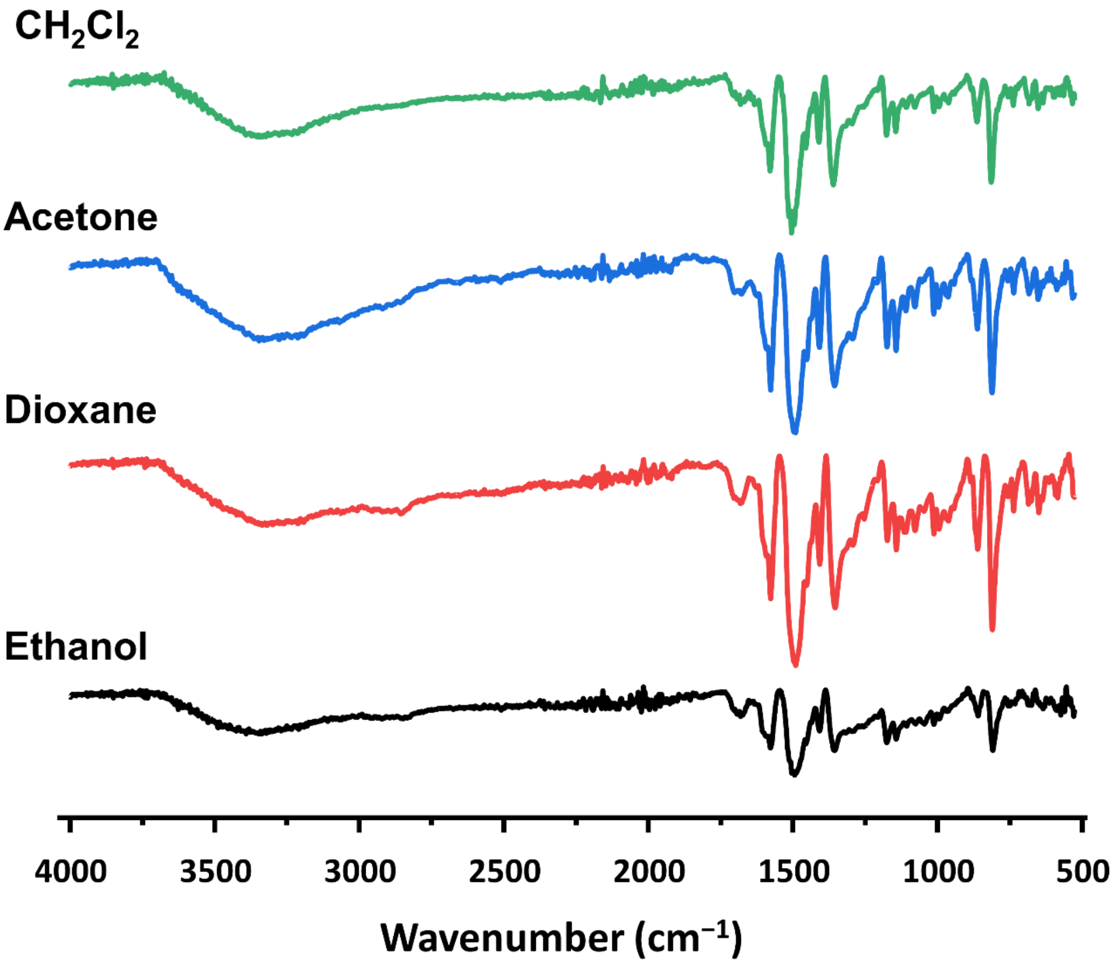

Figure S41. Stacked FTIR spectra of TTA-DFP-COF-**120** in various organic solvents. Membranes were immersed for 24 hours in the various conditions.

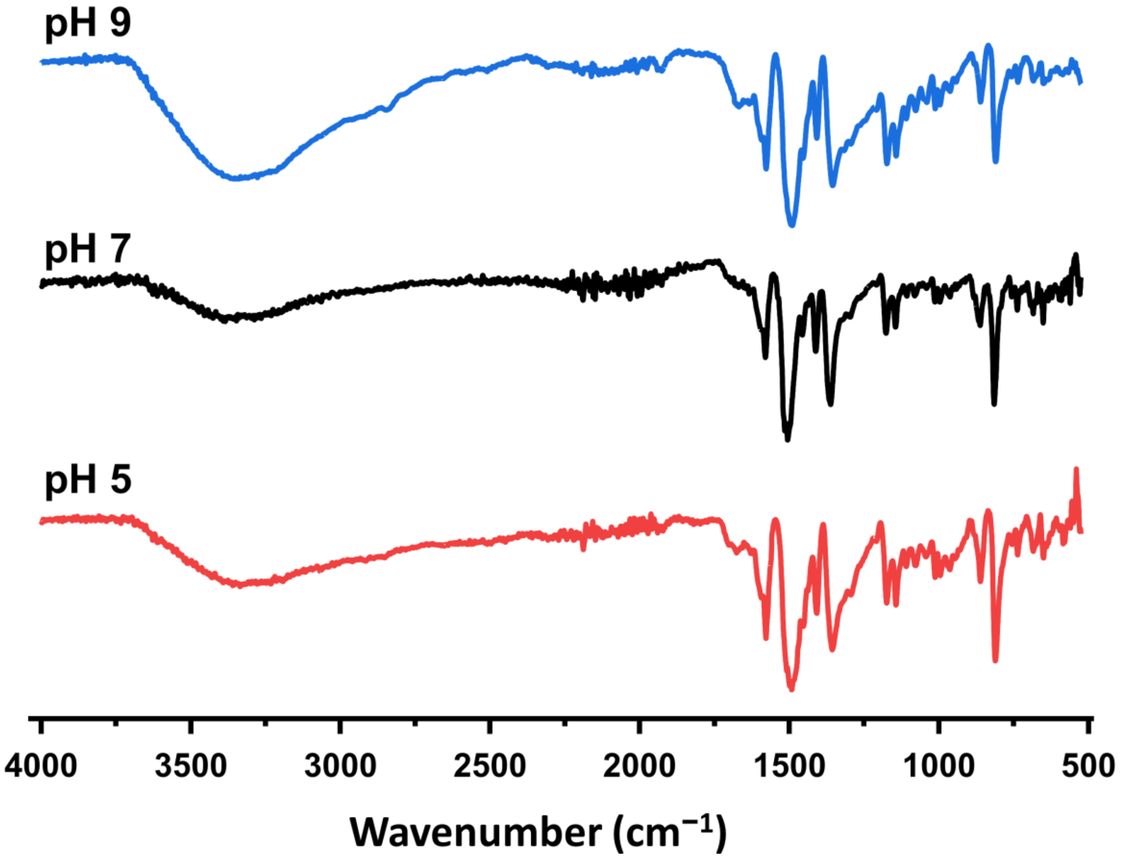

Figure S42. Stacked FTIR spectra of TTA-DFP-COF-**120** in an aqueous solution at pH 5, 7, and 9. Membranes were immersed for 24 hours in the various conditions.

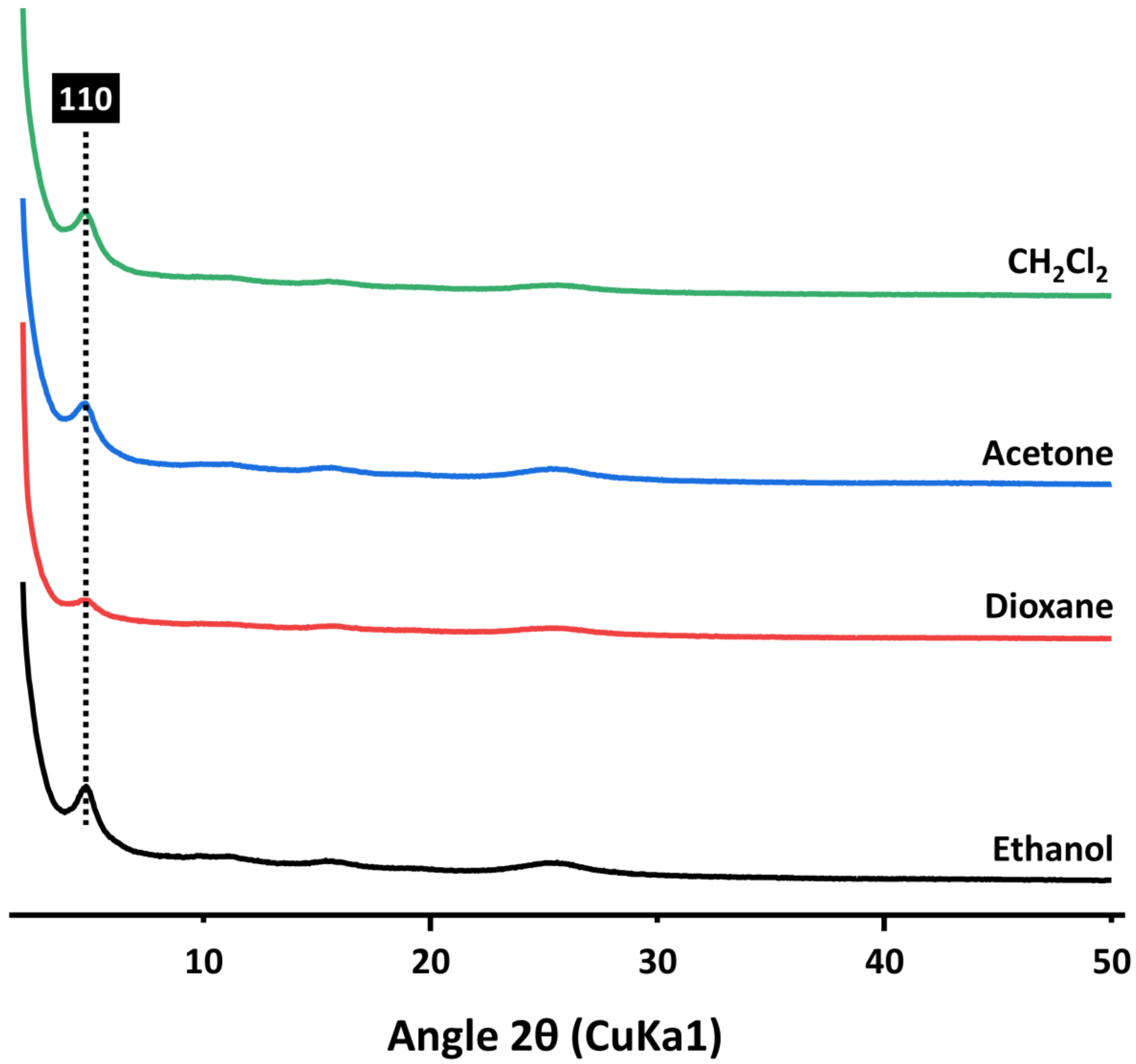

Figure S43. Stacked PXRD of TTA-DFP-COF-**120** in various organic solvents. Membranes were immersed for 24 hours in the various conditions.

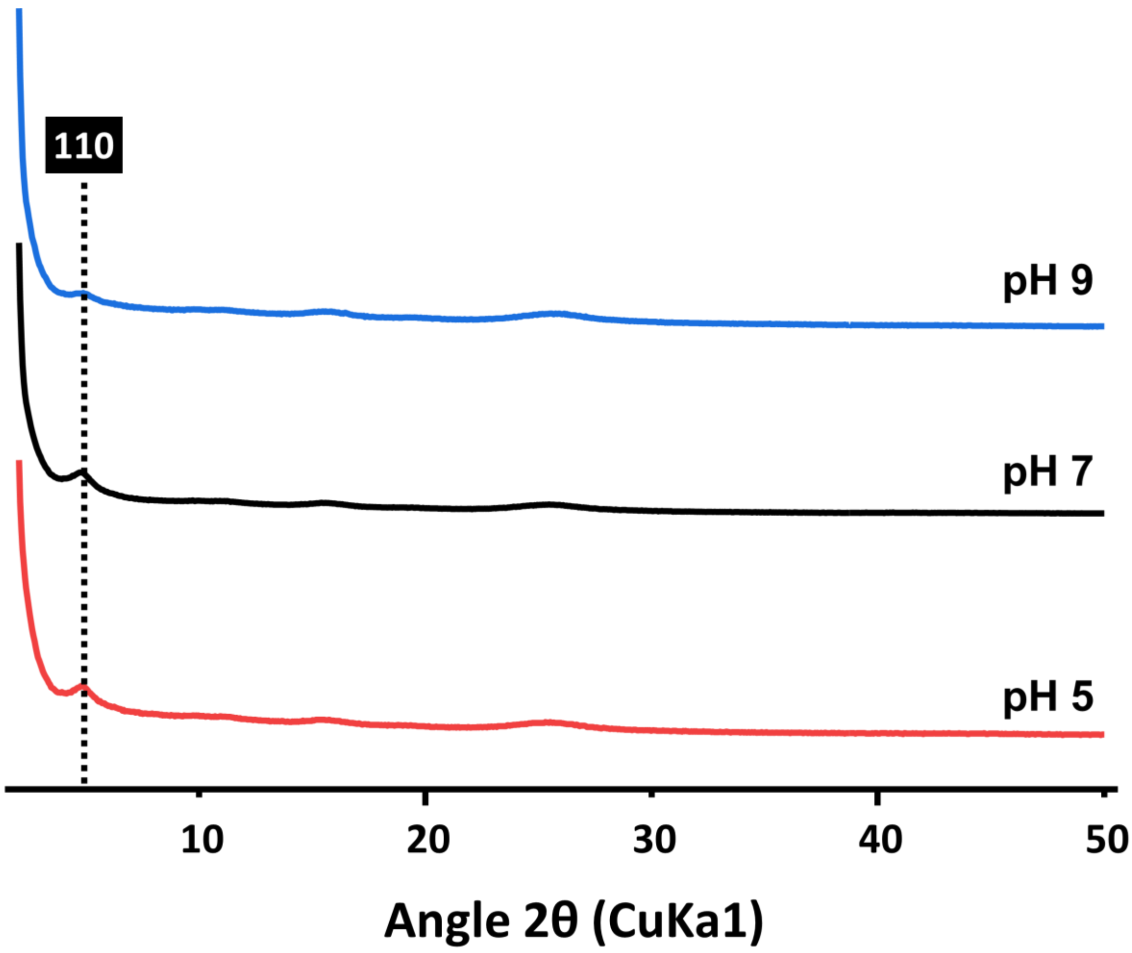

Figure S44. Stacked PXRD of TTA-DFP-COF-**120** in aqueous solution at pH 5, 7, and 9. Membranes were immersed for 24 hours in the various conditions.

4 - Filtration Tests

Before testing, the TTA-DFP-COF membrane was thoroughly washed with water by immersing it in a water bath and changing the water at least three times. The TTA-DFP-COF membrane was then cut into a 1.4 cm circle using a hole punch. A vacuum filtration setup was used to test the TTA-DFP-COF membrane. A membrane holder was attached to the filtration setup and acted as a reducer of the filtration area to a 1.4 cm diameter circle. Another important function of the membrane holder was to sandwich the membrane and prevent its buckling and further damage when the vacuum pump was turned on. Non-woven nylon support was used to support and protect the membrane from both sides, and their size was also adjusted to a 1.4-diameter circle using the hole punch.

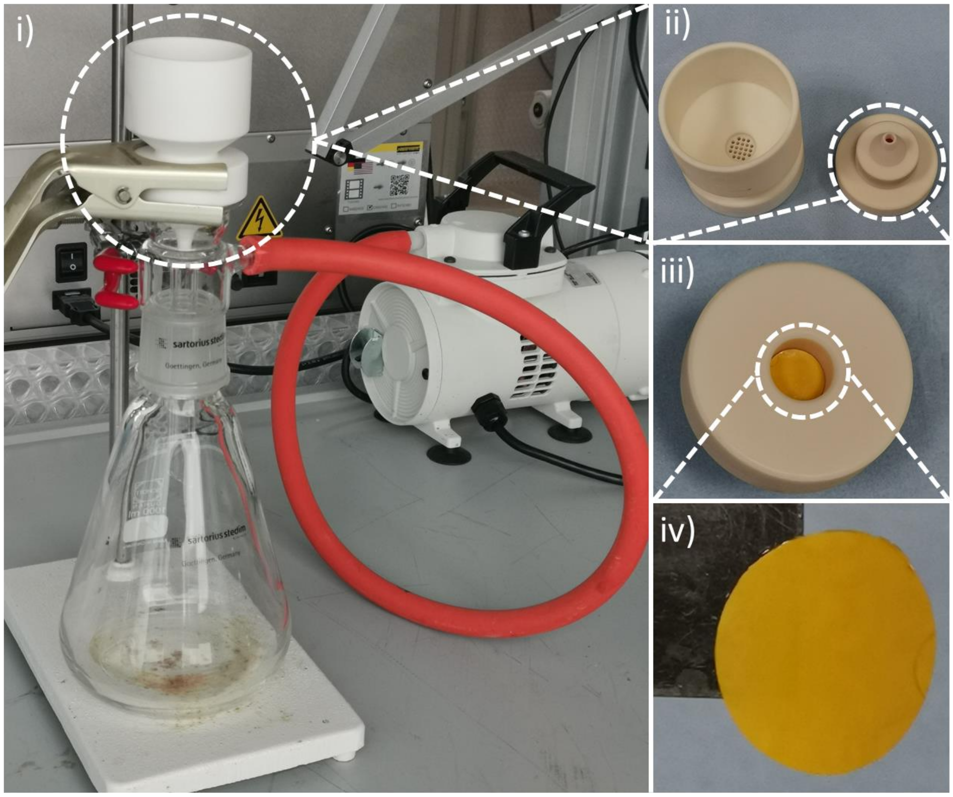

Figure S45. Filtration set-up consisting of i) the vacuum filtration setup used, ii) the membrane holder formed from the upper part, which is a cup that holds the feed and upper support that sandwiches the membrane, and the lower part where the membrane is placed, iii) the lower part

of the membrane holder showing the space where the membrane is placed, and iv) a picture of the membrane cut to 1.4 cm diameter circle as used in the filtration setup.

The salt solutions were obtained by dissolving 1 g of NaCl or $MgCl_2$ in 1L of water, yielding a 1000 ppm salt solution. The dye solutions were prepared by dissolving 2.5 mg of each dye in 1 L of water. Oil in water suspensions were prepared by adding 1g of oil to 1 L of water. A stable oil in water suspension was obtained by mixing the solution for 15 minutes using a homogenizer at 14000 rpm. The pH of all solutions was monitored and adjusted to 7 ±0.05. The filtration setup was cleaned and dried before all experiments. 50 mL of the salt/dye solution or oil in water suspension was added to the filtration cup, and the vacuum pump was turned on. The average transmembrane pressure during our vacuum filtration experiments was 0.5 bar.

Standard procedures were followed to prepare the filtration setup prior to conducting membrane filtration tests. Specifically, the setup underwent thorough flushing with deionized (DI) water to remove any residual contaminants and to ensure optimal conditions for membrane performance evaluation. Additionally, the filtration setup was allowed to run without collecting any samples during the initial run of each test. This practice allowed sufficient time for the system to stabilize, ensuring a stable filtration flux before formal sampling was performed.

After obtaining an adequate amount of filtrate, samples from the filtrate, feed, and retentate were taken and stored. The volume of the filtrate and the time required to collect it are noted to calculate the flux. The salt rejection was calculated using a conductivity meter (Orion VersaStar Pro, Thermo Scientific). The dye rejection was calculated using a UV-VIS (UV–3100PC, VWR). The oil rejection was determined using a total organic carbon (TOC) analyzer (Sievers InnovOx ES). DI water was used to clean the membrane between filtration runs to keep the membrane performance optimal, even when no flux drop was observed.

The filtration experiments methodology, the samples' volume collected, and the number of cycles done are favorable compared to recent literature.[29-34]

The rejection of the salts, dyes, and oil was calculated using the following equation:

$$R = \left(\frac{C_F - C_P}{C_F}\right) \times 100\%$$

Where (R) is the rejection of the salt, dyes, and oil, (C_F) is their concentrations in the feed, while (C_P) is their concentrations in the permeate. The water flux was calculated using the following equation:

$$F = \frac{V}{A \times t}$$

Where (F) is the flux in (L m^{-2} h^{-1}), (V) is the volume of permeate in (L), A is the filtration area in (m^2), and (t) is the time in (h).

All experiments were performed at least three times, and the average and standard deviation were calculated. The membrane was flushed with DI water between tests to clear out any residuals. Blank tests were also performed where the nonwoven supports were placed in the filter holder to confirm that they did not contribute to the rejection.

Table S8. Characteristics and molecular structures of the different dyes studied.

Dye	Molecular weight (g/mol)	Charge	Size (Å)	Molecular structure
**Rose Bengal (RB)**	1017.6	−2	11.2 × 12.4	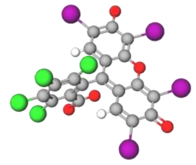
**Methyl Blue (MB)**	799.8	−3	21.3 × 16.8	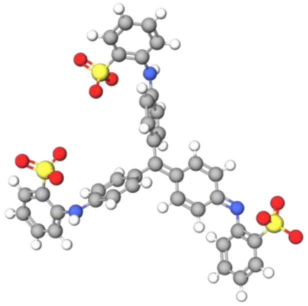
**Naphthol Blue Black (NBB)**	616.5	−2	14.6 × 7.9	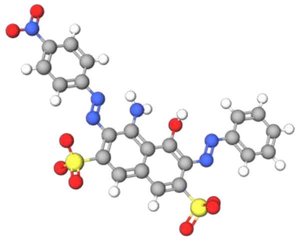
**Methyl Orange (MO)**	327.3	−1	12.1 × 2.4	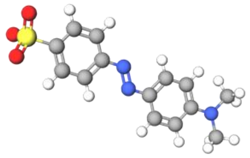

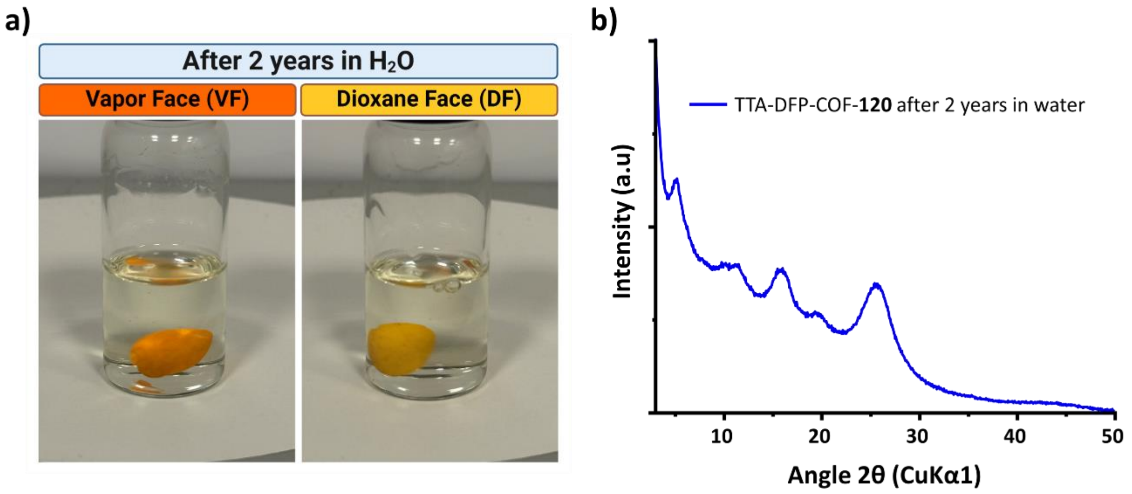

Figure S46. Long-term Stability Test of the TTA-DFP-COF Membrane. a) Pictures of the membrane's Vapor Face (VF) and Dioxane Face (DF) after filtration experiments and being submerged in water for two years, highlighting the preservation of surface textures. b) PXRD pattern of the same membrane post-exposure, indicating that it retained crystallinity and structural integrity.

Table S9. Comparison of the performance of the TTA-DFP-COF membrane with COF, MOF, polymer membranes and composite polymer membranes in water filtration from oil-in-water emulsions

Composite Membrane	Permeation Flux (L m^{-2} h^{-1} bar^{-1})	Separation Efficiency (%)	Number of Cycles	Reference
TTA-DFP-COF-**120**	7200	99.9	10	This Study
**COF–COOH**	1843	>98	5	[35]
**TpHZ COF Nanofibers**	38842	>95	5	[36]
**PVDF/PDA/PEI**	233	>85	3	[37]
**PAI (HFPS)**	440	99.9	18	[38]
**PVDF/ESP**	229	99.5	10	[39]
**PVDF-TiO_2**	400	99.7	5	[40]
**PAN/PANI**	1300	99.8	15	[41]
**PAN-PA6/PANI**	326	97.8	5	[42]
**PEN/HNTs@GO-PDA**	705	99.4	10	[43]
**PEN@PDA/ATPES**	6121	99.63	10	[44]
**PCL/Tween80**	70	99	-	[45]
**PVA/GO/PPY**	6503	99.5	10	[46]
**PAN/HPEI/PDA**	1600	98.5	10	[47]
**MCNM**	1580	99.6	10	[48]
**PAN/ZIF-8**	2514	99.92	-	[49]
**PAN/MOF-808**	379.3	99.97	10	[50]
**SiC**	980	>90	-	[51]
**HF-SiC70Si30**	2515	98.75	2	[52]
**SiC**	654	93.5	2	[53]
**SiC**	13298	>85	-	[54]

5 - Antibacterial Properties

The antibacterial properties of TTA-DFP-COF membranes were assessed against *E. coli* (Gram-negative) and *S. aureus* (Gram-positive) bacteria. All glassware, solutions, and membrane samples were sterilized using ultraviolet light before the experiment. Bacterial concentration was estimated using the optical density at 600 nm (OD_{600}) using a spectrophotometer. The overnight cultures were diluted in Luria-Bertani (LB) medium to achieve target OD_{600} values. Before the bacteria experiment, the membrane was washed and rinsed with sterile PBS. CFU: number of colony-forming unit

All the images presented in this study are representative of results obtained from three independent experiments.

5.1. Disk diffusion

These bacterial cultures were introduced into fresh, sterilized media at a 1:100 volume ratio and incubated at 37 °C for 3 hours with shaking. TTA-DFP-COF membranes, cut into 2.5 cm diameter disks, were placed on the plate, then coated with 100 µL of bacterial suspensions (10^7 CFU/mL) and incubated at 37 °C for 24 hours. The antibacterial efficacy of the membranes was determined by measuring the diameter of the inhibition zones, with larger diameters indicating more effective antibacterial activity.

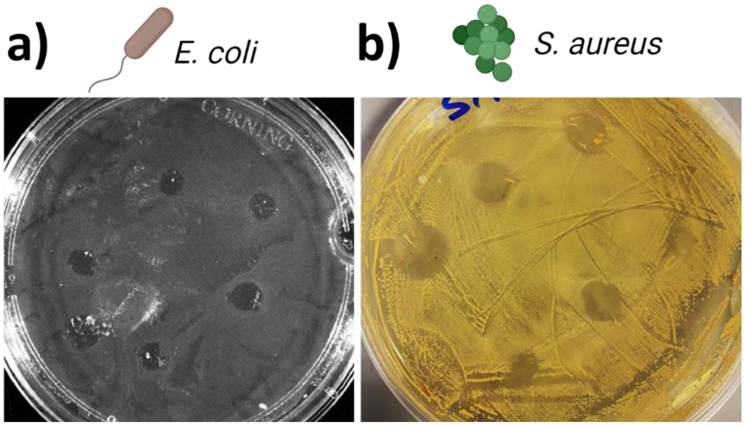

Figure S47. Images of the LB agar plates of a) *E. coli* and b) *S. aureus* cultures after removal of the membranes showing the inhibition zones after 24 hour-contact.

5.2. Antibacterial activity

2.5-cm membrane sample was placed in a Petri dish, immersed in 1 mL of *E. coli* or *S. aureus* suspension (bacteria concentration= 1 10^6 CFU/mL) respectively, and incubated with shaking at medium speed and 37 °C for 1 hour while ensuring the membrane surface was uniformly in contact with the bacteria liquid.

Blank control group: the same *E. coli* and *S. aureus* suspensions (cell concentration= 1 10^6 CFU/mL) without being contacted with the membrane were cultured in constant temperature incubation at 37 °C for the same time with the inoculated membrane groups, as well as LB alone and membrane with only LB (no bacteria) for the sake of comparison. The experiment was conducted in triplicate.

100 µL of the bacterial solution was spread on the LB agar plate, and incubated at 37 °C for 24 h, and the CFUs in each plate was counted to evaluate the antibacterial performance.

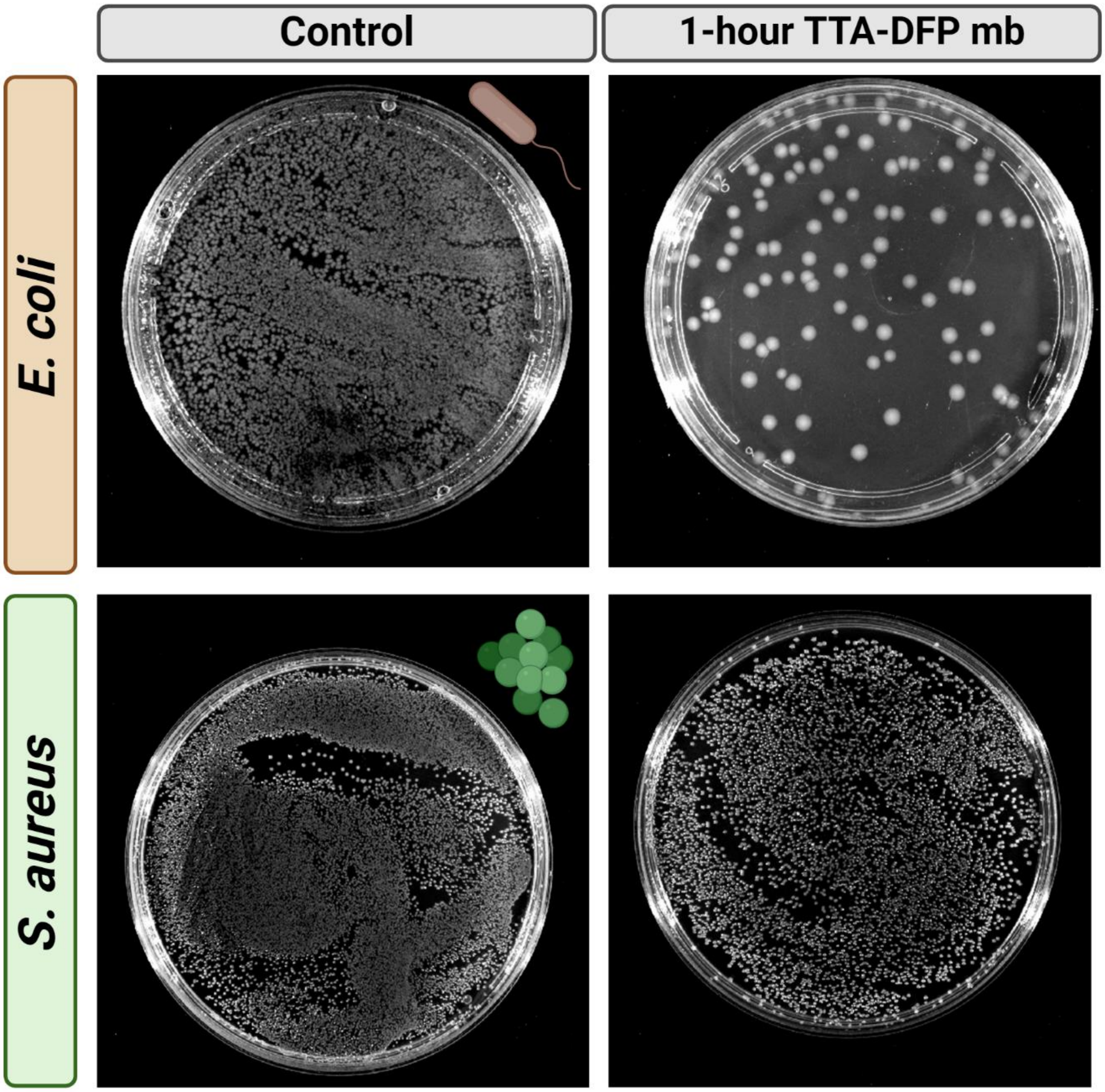

Figure S48. Antibacterial activity of TTA-DFP-COF membrane against *E. coli* and *S. aureus.* Control groups show dense growth of both bacteria. After 1-hour treatment with the TTA-DFP-COF membrane, there is a clear reduction in *E. coli* and *S. aureus* colonies, indicating an inhibition rate of 83.07 % and 78.37 % respectively. Results are from triplicate experiments.

Table S10. Percentage of inhibition (%) of *E. coli* and *S. aureus* after 1 hour of contact with TTA-DFP-COF membrane.

	**% inhibition**
E. coli	83.07 %
S. aureus	78.37 %

5.3. Bacteria attachment experiment

Subsequently, the TTA-DFP-COF membrane was removed and washed with 10 mL sterile PBS solution to remove nonadherent bacteria and collect the whole cells. The collected bacterial suspensions (0.1 mL) were spread in an LB agar plate. The viable bacterial colonies were counted after being cultured for 24 h at 37 °C (Figure S49 ii and vi). As observed, no viable bacteria were collected from the wash solution of the TTA-DFP-COF membrane compared to the control in both bacteria strains (Figure S49).

The membrane was then transferred to bacterial culture tubes with 5 mL of sterile PBS solution and placed at 37 °C in an incubator-shaker for 4 h. 100 mL of the eluent bacterial solution was spread on an LB agar plate. The plates were incubated at 37 °C for 24 h, and the viable bacterial colonies in each plate were counted to evaluate the antibacterial and anti-attachment performance. As observed in Figure S49 iii and vii, no viable bacteria were collected from the eluent bacterial solution compared to the control in both bacterial strains, showing that no bacteria were attached to the membrane or survived its contact.

The membrane was subsequently placed on an agar plate at 37 °C for 24 hours. The absence of colonies indicated that no bacteria, from either strain, adhered to the membrane, as illustrated in Figure S49 iv and viii.

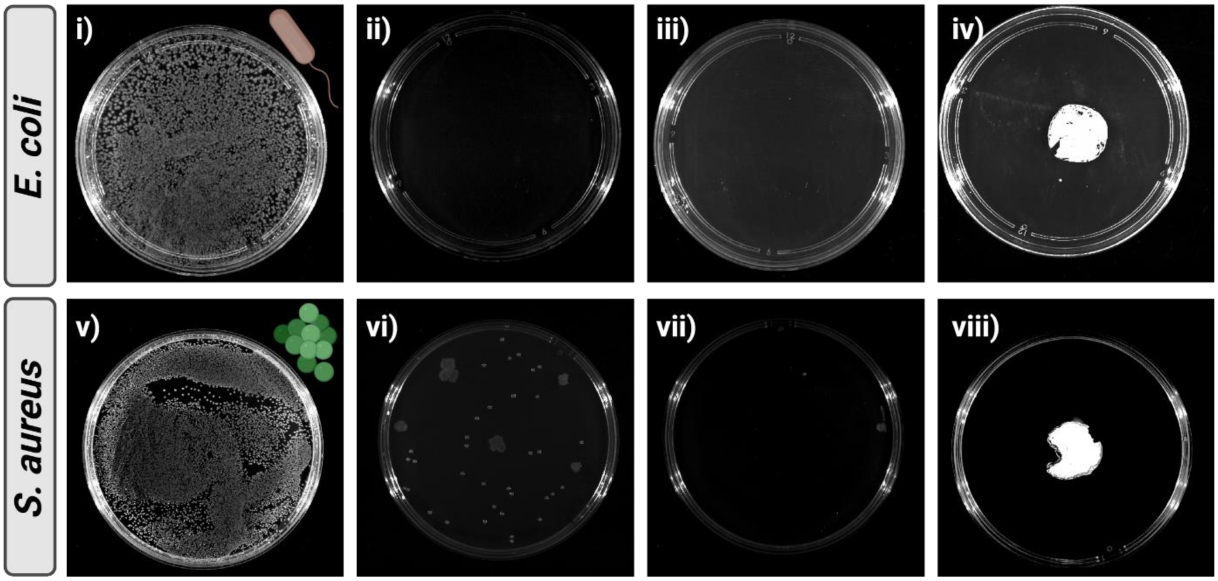

Figure S49. Anti-attachment efficacy of TTA-DFP-COF membrane against *E. coli* (top panel) and *S. aureus* (bottom panel). i) and v) Display control agar plates with bacterial growth; ii) and vi) Show no bacterial colonies from the wash solution post-membrane contact; iii) and vii) Indicate absence of bacterial colonies in the eluent from the membrane incubation; iv) and viii) Confirm no bacterial adherence on the membrane after 24 hours on agar plates.

5.4. Bacterial morphology study by SEM

To investigate the morphological changes in bacteria exposed to the TTA-DFP-COF membrane for 1 hour, we conducted SEM analysis in comparison to control. Initially, the bacteria were fixed overnight using a 2.5 % glutaraldehyde solution. Subsequently, the specimens underwent two rounds of washing with phosphate-buffered saline (PBS) at a pH of 7.4. To further prepare the samples for analysis, we subjected them to a series of ethanol washes with increasing concentrations (20 %, 40 %, 60 %, 80 %, and 100 %). Finally, a gold coating was applied to the prepared bacterial samples for SEM analysis.

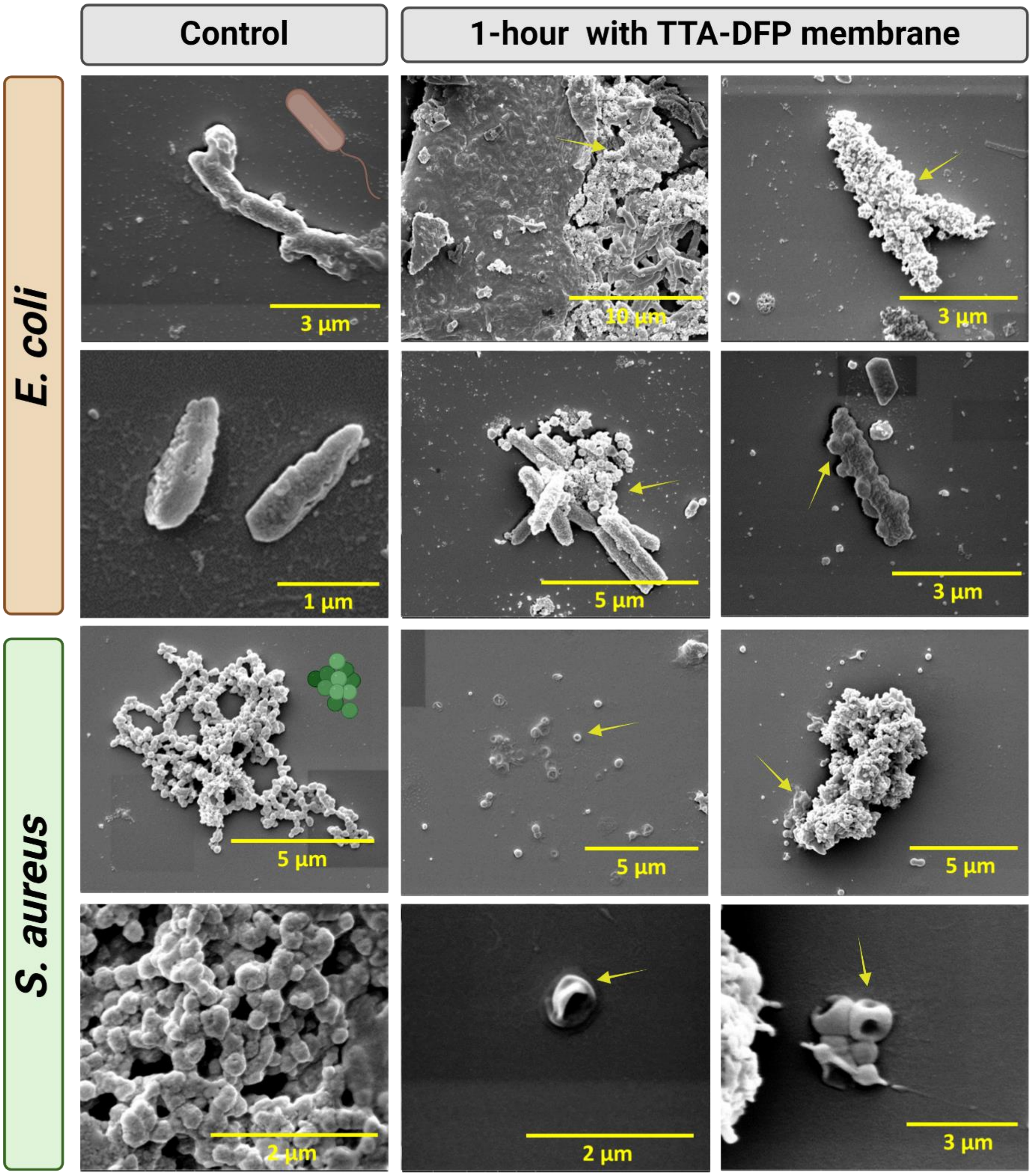

Figure S50. Comparative SEM analysis of bacterial morphology in the presence and absence of the TTA-DFP-COF membrane. Top panel: *E. coli* bacteria appear intact in control samples but show notable deformation after 1-hour exposure to the TTA-DFP-COF membrane, as indicated by the yellow arrows. Bottom panel: *S. aureus* exhibits a similar trend, with control bacteria

maintaining their characteristic shape and those treated with the TTA-DFP-COF membrane displaying significant structural disruptions, highlighted by yellow arrows.

5.5. Biocompatibility of the TTA-DFP-COF membrane

The biocompatibility of the TTA-DFP-COF membrane was rigorously evaluated *in vitro* using the Human Embryonic Kidney (HEK-293) cell line. This cell line, known for its epithelial morphology and origin from human embryonic kidney tissue, is extensively utilized in biotechnology and toxicology research for evaluating material compatibility with human biology.

HEK-293 (ATCC CRL-1573) cell lines were cultured in Dulbecco's Modified Eagle's medium (DMEM) supplemented with 10 % fetal bovine serum (FBS) and 1 % penicillin/streptomycin at 5 % CO_2 and 37 °C.

In vitro study by optical microscopy

HEK-293 cells were seeded in 6-well plates for 24 hours in complete DMEM with fragments of TTA-DFP-COF membrane. Then, the cells were washed with PBS, fixed with paraformaldehyde solution (3.7 %) for 10 minutes, and washed thrice with PBS. Samples were analysed using optical microscopy. The experiment was repeated 3 times.

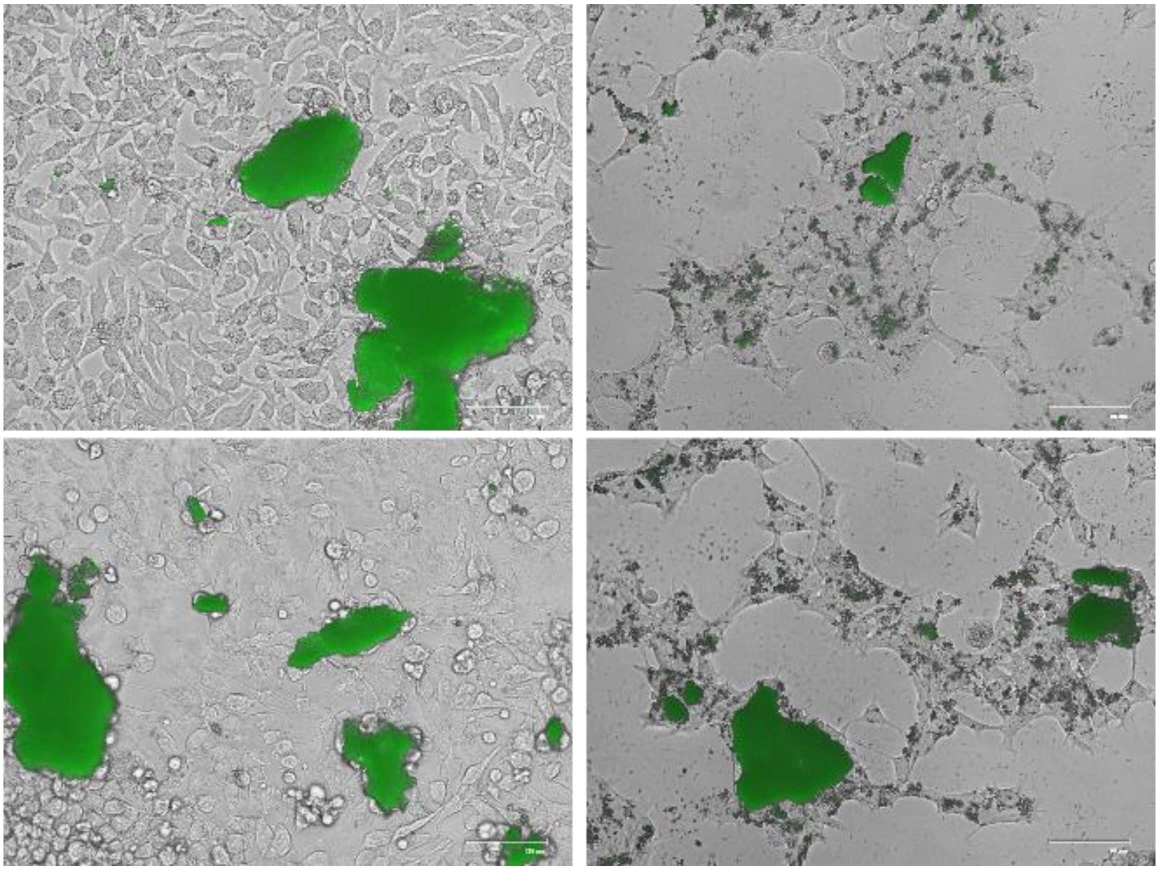

Figure S51. Images of HEK-293 cells incubated with TTA-DFP-COF membrane for 48 hours (membrane fragments are displayed in green).

References

1. Liu, J.; Han, G.; Zhao, D.; Lu, K.; Gao, J.; Chung, T.-S., Self-standing and flexible covalent organic framework (COF) membranes for molecular separation. *Science Advances* **2020,** *6* (41), eabb1110.
2. Li, B.; Wang, Z.; Gao, Z.; Suo, J.; Xue, M.; Yan, Y.; Valtchev, V.; Qiu, S.; Fang, Q., Self-Standing Covalent Organic Framework Membranes for H2/CO2 Separation. *Advanced Functional Materials* **2023,** *33* (16), 2300219.
3. Khan, N. A.; Zhang, R.; Wang, X.; Cao, L.; Azad, C. S.; Fan, C.; Yuan, J.; Long, M.; Wu, H.; Olson, M. A.; Jiang, Z., Assembling covalent organic framework membranes via phase switching for ultrafast molecular transport. *Nature Communications* **2022,** *13* (1), 3169.
4. He, J.; Yu, L.; Li, Z.; Ba, S.; Lan, F.; Wu, Y., Catalyst regulated interfacial synthesis of self-standing covalent organic framework membranes at room temperature for molecular separation. *Journal of Colloid and Interface Science* **2023,** *629*, 428-437.
5. Yang, R.; Liu, S.; Sun, Q.; Liao, Q.; Xi, K.; Su, B., Potential Difference-Modulated Synthesis of Self-Standing Covalent Organic Framework Membranes at Liquid/Liquid Interfaces. *Journal of the American Chemical Society* **2022,** *144* (26), 11778-11787.
6. Zhao, S.; Jiang, C.; Fan, J.; Hong, S.; Mei, P.; Yao, R.; Liu, Y.; Zhang, S.; Li, H.; Zhang, H.; Sun, C.; Guo, Z.; Shao, P.; Zhu, Y.; Zhang, J.; Guo, L.; Ma, Y.; Zhang, J.; Feng, X.; Wang, F.; Wu, H.; Wang, B., Hydrophilicity gradient in covalent organic frameworks for membrane distillation. *Nature Materials* **2021,** *20* (11), 1551-1558.
7. Mohammed, A. K.; Al Khoori, A. A.; Addicoat, M. A.; Varghese, S.; Othman, I.; Jaoude, M. A.; Polychronopoulou, K.; Baias, M.; Haija, M. A.; Shetty, D., Solvent-Influenced Fragmentations in Free-Standing Three-Dimensional Covalent Organic Framework Membranes for Hydrophobicity Switching. *Angewandte Chemie International Edition* **2022,** *61* (13), e202200905.
8. Qu, Y.; Zha, Y.; Du, X.; Xu, S.; Zhang, M.; Xu, L.; Jia, H., Interfacial Polymerization of Self-Standing Covalent Organic Framework Membranes at Alkane/Ionic Liquid Interfaces for Dye Separation. *ACS Applied Polymer Materials* **2022,** *4* (10), 7528-7536.
9. Fan, C.; Geng, H.; Wu, H.; Peng, Q.; Wang, X.; Shi, B.; Kong, Y.; Yin, Z.; Liu, Y.; Jiang, Z., Three-dimensional covalent organic framework membrane for efficient proton conduction. *Journal of Materials Chemistry A* **2021,** *9* (33), 17720-17723.
10. Prakasam, T.; Lusi, M.; Elhabiri, M.; Platas-Iglesias, C.; Olsen, J.-C.; Asfari, Z.; Cianférani-Sanglier, S.; Debaene, F.; Charbonnière, L. J.; Trabolsi, A., Simultaneous Self-Assembly of a [2]Catenane, a Trefoil Knot, and a Solomon Link from a Simple Pair of Ligands. *Angew. Chem. Int. Ed. Engl.* **2013,** *52* (38), 9956-9960.
11. Lu, Y.; Zhou, Z.-B.; Qi, Q.-Y.; Yao, J.; Zhao, X., Polyamide Covalent Organic Framework Membranes for Molecular Sieving. *ACS Applied Materials & Interfaces* **2022,** *14* (32), 37019-37027.
12. Turangan, N.; Xu, Y.; Spratt, H.; Rintoul, L.; Bottle, S.; MacLeod, J., Self-supporting covalent organic framework membranes synthesized through two different processes: solvothermal annealing and solvent vapor annealing. *Nanotechnology* **2021,** *32* (7), 075604.
13. Dey, K.; Bhunia, S.; Sasmal, H. S.; Reddy, C. M.; Banerjee, R., Self-Assembly-Driven Nanomechanics in Porous Covalent Organic Framework Thin Films. *Journal of the American Chemical Society* **2021,** *143* (2), 955-963.
14. Wang, H.; Zhao, J.; Li, Y.; Cao, Y.; Zhu, Z.; Wang, M.; Zhang, R.; Pan, F.; Jiang, Z., Aqueous Two-Phase Interfacial Assembly of COF Membranes for Water Desalination. *Nano-Micro Letters* **2022,** *14* (1), 216.
15. Zhao, Z.; Wang, R.; Peng, C.; Chen, W.; Wu, T.; Hu, B.; Weng, W.; Yao, Y.; Zeng, J.; Chen, Z.; Liu, P.; Liu, Y.; Li, G.; Guo, J.; Lu, H.; Guo, Z., Horizontally arranged zinc platelet electrodeposits modulated

by fluorinated covalent organic framework film for high-rate and durable aqueous zinc ion batteries. *Nature Communications* **2021,** *12* (1), 6606.
16. Wang, Z.; Yu, Q.; Huang, Y.; An, H.; Zhao, Y.; Feng, Y.; Li, X.; Shi, X.; Liang, J.; Pan, F.; Cheng, P.; Chen, Y.; Ma, S.; Zhang, Z., PolyCOFs: A New Class of Freestanding Responsive Covalent Organic Framework Membranes with High Mechanical Performance. *ACS central science* **2019,** *5* (8), 1352-1359.
17. Martín-Illán, J.; Suárez, J. A.; Gómez-Herrero, J.; Ares, P.; Gallego-Fuente, D.; Cheng, Y.; Zhao, D.; Maspoch, D.; Zamora, F., Ultralarge Free-Standing Imine-Based Covalent Organic Framework Membranes Fabricated via Compression. *Advanced science (Weinheim, Baden-Wurttemberg, Germany)* **2022,** *9* (7), e2104643.
18. Zhu, D.; Hu, Z.; Rogers, T. K.; Barnes, M.; Tseng, C.-P.; Mei, H.; Sassi, L. M.; Zhang, Z.; Rahman, M. M.; Ajayan, P. M.; Verduzco, R., Patterning, Transfer, and Tensile Testing of Covalent Organic Framework Films with Nanoscale Thickness. *Chemistry of Materials* **2021,** *33* (17), 6724-6730.
19. Duan, K.; Wang, J.; Zhang, Y.; Liu, J., Covalent organic frameworks (COFs) functionalized mixed matrix membrane for effective CO2/N2 separation. *Journal of Membrane Science* **2019,** *572*, 588-595.
20. Wang, Y.; Guo, B.; Yang, T.; Zhang, Z.-C.; Liang, L.; Ding, S.-Y.; Wang, W., Modulated Synthesis of Self-Standing Covalent Organic Framework Films. *Chemistry – A European Journal* **2022,** *28* (46), e202200961.
21. Hou, S.; Ji, W.; Chen, J.; Teng, Y.; Wen, L.; Jiang, L., Free-Standing Covalent Organic Framework Membrane for High-Efficiency Salinity Gradient Energy Conversion. *Angewandte Chemie International Edition* **2021,** *60* (18), 9925-9930.
22. Mishra, B.; Tripathi, B. P., Flexible covalent organic framework membranes with linear aliphatic amines for enhanced organic solvent nanofiltration. *Journal of Materials Chemistry A* **2023,** *11* (30), 16321-16333.
23. Bundschuh, S.; Kraft, O.; Arslan, H. K.; Gliemann, H.; Weidler, P. G.; Wöll, C., Mechanical properties of metal-organic frameworks: An indentation study on epitaxial thin films. *Applied Physics Letters* **2012,** *101* (10).
24. Zhang, Y.; Gao, Q.; Lin, Z.; Zhang, T.; Xu, J.; Tan, Y.; Tian, W.; Jiang, L., Constructing Free Standing Metal Organic Framework MIL-53 Membrane Based on Anodized Aluminum Oxide Precursor. *Scientific Reports* **2014,** *4* (1), 4947.
25. Chen, S.; Zhao, D.; Feng, Y.; Liu, H.; Li, S.; Qiu, Y.; Ren, J., The preparation and characterization of gel-mixed matrix membranes (g-MMMs) with high CO2 permeability and stability performance. *Journal of Membrane Science* **2022,** *652*, 120471.
26. Altabal, O.; Wischke, C., Analyzing the Mechanical Properties of Free-Standing PACA Thin Films Using Microindentation Technique. *Polymers* **2022,** *14* (22), 4863.
27. Xu, Y.; Tsai, Y.; Zheng, D. W.; Tu, K. N.; Wo Ong, C.; Choy, C. L.; Zhao, B.; Liu, Q.-Z.; Brongo, M., Measurement of mechanical properties for dense and porous polymer films having a low dielectric constant. *Journal of Applied Physics* **2000,** *88* (10), 5744-5750.
28. Bais, B.; Yusof, N.; Soin, N.; Yunas, J.; Majlis, B. Y., Development and Characterization of Freestanding Poly (Methyl Methacrylate)/Monolayer Graphene Membrane. *IEEE Access* **2020,** *8*, 29702-29710.
29. Kandambeth, S.; Biswal, B. P.; Chaudhari, H. D.; Rout, K. C.; Kunjattu H., S.; Mitra, S.; Karak, S.; Das, A.; Mukherjee, R.; Kharul, U. K.; Banerjee, R., Selective Molecular Sieving in Self-Standing Porous Covalent-Organic-Framework Membranes. *Advanced Materials* **2017,** *29* (2), 1603945.
30. He, Y.; Lin, X.; Chen, J.; Guo, Z.; Zhan, H., Homogeneous Polymerization of Self-standing Covalent Organic Framework Films with High Performance in Molecular Separation. *ACS Applied Materials & Interfaces* **2020,** *12* (37), 41942-41949.

31. Zhang, W.; Zhang, L.; Zhao, H.; Li, B.; Ma, H., A two-dimensional cationic covalent organic framework membrane for selective molecular sieving. *Journal of Materials Chemistry A* **2018,** *6* (27), 13331-13339.
32. Dey, K.; Pal, M.; Rout, K. C.; Kunjattu H, S.; Das, A.; Mukherjee, R.; Kharul, U. K.; Banerjee, R., Selective Molecular Separation by Interfacially Crystallized Covalent Organic Framework Thin Films. *Journal of the American Chemical Society* **2017,** *139* (37), 13083-13091.
33. Fenton, J. L.; Burke, D. W.; Qian, D.; Olvera de la Cruz, M.; Dichtel, W. R., Polycrystalline Covalent Organic Framework Films Act as Adsorbents, Not Membranes. *Journal of the American Chemical Society* **2021,** *143* (3), 1466-1473.
34. Liu, X.; Wang, J.; Shang, Y.; Yavuz, C. T.; Khashab, N. M., Ionic Covalent Organic Framework-Based Membranes for Selective and Highly Permeable Molecular Sieving. *Journal of the American Chemical Society* **2024,** *146* (4), 2313-2318.
35. Liang, Q.; Jiang, B.; Yang, N.; Zhang, L.; Sun, Y.; Zhang, L., Superhydrophilic Modification of Polyvinylidene Fluoride Membrane via a Highly Compatible Covalent Organic Framework–COOH/Dopamine-Integrated Hierarchical Assembly Strategy for Oil–Water Separation. *ACS Applied Materials & Interfaces* **2022,** *14* (40), 45880-45892.
36. Sun, Q.; Du, J.; Wang, L.; Yao, A.; Song, Z.; Liu, L.; Cao, D.; Ma, J.; Lim, W.; He, W.; Ul Hassan, S.; Zhou, C.; Liu, J., Smart superwetting COF membrane for controllable oil/water separation. *Separation and Purification Technology* **2023,** *317,* 123825.
37. Xiong, Z.; He, Z.; Mahmud, S.; Yang, Y.; Zhou, L.; Hu, C.; Zhao, S., Simple amphoteric charge strategy to reinforce superhydrophilic polyvinylidene fluoride membrane for highly efficient separation of various surfactant-stabilized oil-in-water emulsions. *ACS Applied Materials & Interfaces* **2020,** *12* (41), 47018-47028.
38. Asad, A.; Rastgar, M.; Sameoto, D.; Sadrzadeh, M., Gravity assisted super high flux microfiltration polyamide-imide membranes for oil/water emulsion separation. *Journal of Membrane Science* **2021,** *621,* 119019.
39. Zhang, H.; Shen, Y.; Li, M.; Zhu, G.; Feng, H.; Li, J., Egg shell powders-coated membrane for surfactant-stabilized crude oil-in-water emulsions efficient separation. *ACS Sustainable Chemistry & Engineering* **2019,** *7* (12), 10880-10887.
40. Deng, W.; Fan, T.; Li, Y., In situ biomineralization-constructed superhydrophilic and underwater superoleophobic PVDF-TiO2 membranes for superior antifouling separation of oil-in-water emulsions. *Journal of Membrane Science* **2021,** *622,* 119030.
41. Shakiba, M.; Nabavi, S. R.; Emadi, H.; Faraji, M., Development of a superhydrophilic nanofiber membrane for oil/water emulsion separation via modification of polyacrylonitrile/polyaniline composite. *Polymers for Advanced Technologies* **2021,** *32* (3), 1301-1316.
42. Faraji, M.; Nabavi, S. R.; Salimi-Kenari, H., Fabrication of a PAN–PA6/PANI membrane using dual spinneret electrospinning followed by in situ polymerization for separation of oil-in-water emulsions. *New Journal of Chemistry* **2020,** *44* (31), 13488-13500.
43. He, S.; Zhan, Y.; Bai, Y.; Hu, J.; Li, Y.; Zhang, G.; Zhao, S., Gravity-driven and high flux super-hydrophobic/super-oleophilic poly (arylene ether nitrile) nanofibrous composite membranes for efficient water-in-oil emulsions separation in harsh environments. *Composites Part B: Engineering* **2019,** *177,* 107439.
44. Panatdasirisuk, W.; Liao, Z.; Vongsetskul, T.; Yang, S., Separation of Oil-in-Water Emulsions Using Hydrophilic Electrospun Membranes with Anisotropic Pores. *Langmuir* **2017,** *33* (23), 5872-5878.
45. Zhang, D.; Jin, X.-Z.; Huang, T.; Zhang, N.; Qi, X.-d.; Yang, J.-h.; Zhou, Z.-w.; Wang, Y., Electrospun fibrous membranes with dual-scaled porous structure: super hydrophobicity, super lipophilicity, excellent water adhesion, and anti-icing for highly efficient oil adsorption/separation. *ACS applied materials & interfaces* **2019,** *11* (5), 5073-5083.

46. Chen, S.; Liu, Y.; Wang, Y.; Xu, K.; Zhang, X.; Zhong, W.; Luo, G.; Xing, M., Dual-functional superwettable nano-structured membrane: From ultra-effective separation of oil-water emulsion to seawater desalination. *Chemical Engineering Journal* **2021,** *411*, 128042.
47. Wang, J.; Hou, L. a.; Yan, K.; Zhang, L.; Yu, Q. J., Polydopamine nanocluster decorated electrospun nanofibrous membrane for separation of oil/water emulsions. *Journal of Membrane Science* **2018,** *547*, 156-162.
48. Wu, M.; Liu, W.; Mu, P.; Wang, Q.; Li, J., Sacrifice template strategy to the fabrication of a self-cleaning nanofibrous membrane for efficient crude oil-in-water emulsion separation with high flux. *ACS Applied Materials & Interfaces* **2020,** *12* (47), 53484-53493.
49. Li, H.; Mu, P.; Li, J.; Wang, Q., Inverse desert beetle-like ZIF-8/PAN composite nanofibrous membrane for highly efficient separation of oil-in-water emulsions. *Journal of Materials Chemistry A* **2021,** *9* (7), 4167-4175.
50. Chen, X.; Chen, D.; Li, N.; Xu, Q.; Li, H.; He, J.; Lu, J., Modified-MOF-808-loaded polyacrylonitrile membrane for highly efficient, simultaneous emulsion separation and heavy metal ion removal. *ACS applied materials & interfaces* **2020,** *12* (35), 39227-39235.
51. Jiang, Q.; Wang, Y.; Xie, Y.; Zhou, M.; Gu, Q.; Zhong, Z.; Xing, W., Silicon carbide microfiltration membranes for oil-water separation: Pore structure-dependent wettability matters. *Water Research* **2022,** *216*, 118270.
52. Bessa, L. P.; Ferreira, E. d. P.; Cardoso, V. L.; Reis, M. H. M., Air-sintered silicon (Si)-bonded silicon carbide (SiC) hollow fiber membranes for oil/water separation. *Journal of the European Ceramic Society* **2022,** *42* (2), 402-411.
53. Xu, M.; Xu, C.; Rakesh, K.; Cui, Y.; Yin, J.; Chen, C.; Wang, S.; Chen, B.; Zhu, L., Hydrophilic SiC hollow fiber membranes for low fouling separation of oil-in-water emulsions with high flux. *RSC advances* **2020,** *10* (8), 4832-4839.
54. Das, D.; Kayal, N.; Marsola, G. A.; Damasceno, L. A.; Innocentini, M. D. d. M., Permeability behavior of silicon carbide-based membrane and performance study for oily wastewater treatment. *International Journal of Applied Ceramic Technology* **2020,** *17* (3), 893-906.
